# Cancer-associated fibroblast-derived acetate promotes pancreatic cancer development by altering polyamine metabolism via the ACSS2–SP1–SAT1 axis

**DOI:** 10.1038/s41556-024-01372-4

**Published:** 2024-03-01

**Authors:** Divya Murthy, Kuldeep S. Attri, Surendra K. Shukla, Ravi Thakur, Nina V. Chaika, Chunbo He, Dezhen Wang, Kanupriya Jha, Aneesha Dasgupta, Ryan J. King, Scott E. Mulder, Joshua Souchek, Teklab Gebregiworgis, Vikant Rai, Rohit Patel, Tuo Hu, Sandeep Rana, Sai Sundeep Kollala, Camila Pacheco, Paul M. Grandgenett, Fang Yu, Vikas Kumar, Audrey J. Lazenby, Adrian R. Black, Susanna Ulahannan, Ajay Jain, Barish H. Edil, David L. Klinkebiel, Robert Powers, Amarnath Natarajan, Michael A. Hollingsworth, Kamiya Mehla, Quan Ly, Sarika Chaudhary, Rosa F. Hwang, Kathryn E. Wellen, Pankaj K. Singh

**Affiliations:** 1 Eppley Institute for Research in Cancer and Allied Diseases, University of Nebraska Medical Center, Omaha, NE USA; 2 Department of Oncology Science, University of Oklahoma Health Sciences Center, Oklahoma City, OK USA; 3 Department of Biotechnology, School of Engineering and Applied Sciences, Bennett University, Greater Noida, Uttar Pradesh India; 4 Department of Biochemistry and Molecular Biology, University of Nebraska Medical Center, Omaha, NE USA; 5 Department of Chemistry, University of Nebraska-Lincoln, Lincoln, NE USA; 6 Department of Biochemistry, Schulich School of Medicine & Dentistry, University of Western Ontario, London, Ontario, Canada; 7 Department of Biostatistics, University of Nebraska Medical Center, Omaha, NE USA; 8 Department of Cell Biology, Genetics and Anatomy, University of Nebraska Medical Center, Omaha, NE USA; 9 Department of Pathology and Microbiology, University of Nebraska Medical Center, Omaha, NE USA; 10 Department of Internal Medicine, University of Oklahoma Health Sciences Center, Oklahoma City, OK USA; 11 Department of Surgery, University of Oklahoma Health Sciences Center, Oklahoma City, OK USA; 12 Nebraska Center for Integrated Biomolecular Communication, University of Nebraska–Lincoln, Lincoln, NE USA; 13 Department of Surgical Oncology, University of Nebraska Medical Center, Omaha, NE USA; 14 Department of Surgical Oncology, Division of Surgery, The University of Texas MD Anderson Cancer Center, Houston, TX USA; 15 Department of Cancer Biology, University of Pennsylvania Perelman School of Medicine, Philadelphia, PA USA; 16 OU Health Stephenson Cancer Center, University of Oklahoma Health Sciences Center, Oklahoma City, OK USA

**Keywords:** Pancreatic cancer, Cancer microenvironment, Cancer metabolism

## Abstract

The ability of tumour cells to thrive in harsh microenvironments depends on the utilization of nutrients available in the milieu. Here we show that pancreatic cancer-associated fibroblasts (CAFs) regulate tumour cell metabolism through the secretion of acetate, which can be blocked by silencing ATP citrate lyase (ACLY) in CAFs. We further show that acetyl-CoA synthetase short-chain family member 2 (ACSS2) channels the exogenous acetate to regulate the dynamic cancer epigenome and transcriptome, thereby facilitating cancer cell survival in an acidic microenvironment. Comparative H3K27ac ChIP–seq and RNA–seq analyses revealed alterations in polyamine homeostasis through regulation of *SAT1* gene expression and enrichment of the *SP1*-responsive signature. We identified acetate/ACSS2-mediated acetylation of SP1 at the lysine 19 residue that increased SP1 protein stability and transcriptional activity. Genetic or pharmacologic inhibition of the ACSS2–SP1–SAT1 axis diminished the tumour burden in mouse models. These results reveal that the metabolic flexibility imparted by the stroma-derived acetate enabled cancer cell survival under acidosis via the ACSS2–SP1–SAT1 axis.

## Abstract (web summary)

Murthy et al. demonstrate that cancer-associated fibroblast-derived acetate regulates polyamine homeostasis via an ACSS2–SP1–SAT1 axis in pancreatic cancer cells, thus enabling cell survival and tumour development under acidosis.

## Main

Cancer-associated fibroblasts (CAFs) are a major constituent of the desmoplastic stroma in pancreatic ductal adenocarcinoma (PDAC) and can be derived from pancreatic stellate cells (PSCs)^1^. CAFs help compose the extracellular matrix and supply key nutrients to tumour cells^1^. Although abolishing CAF-activating signals or the fibrotic stroma as a whole has resulted in poor mouse survival^2,3^ and adverse or no effects in a clinical trial^4^, CAFs provide nutritional support for tumour-cell survival in harsh conditions and contribute to their aggressiveness^5,6^. Thus, it is probably more advantageous to target specific features of CAFs that support tumour growth in the harsh milieu, without disrupting the stroma as a whole. Previous studies have demonstrated metabolic alterations in PDAC^7–17^, differential expression of metabolic genes in tumour and stromal components^18^, and a potential role of certain stellate cell-derived metabolites^6^. However, the full repertoire and the signalling and nutritional potential of the stromal cell-secreted metabolic components remain largely unknown.

Acetate, a multifaceted metabolite, is a major carbon source for macromolecule biosynthesis and energy production. Acetate is converted to acetyl-CoA by the acetyl-CoA synthetase (ACSS) family of enzymes^19^. Besides playing a pivotal role in cellular metabolism, acetyl-CoA is associated with regulatory functions mediated through protein acetylation resulting in altered signalling, epigenetic modifications, gene expression, DNA replication and DNA damage repair^20,21^. Correspondingly, [^11^C]-acetate positron emission tomography (PET) studies have indicated an increased uptake of acetate in various cancers and, pertinent to this study, immunohistochemical (IHC) analyses of pancreatic tumour tissues have shown enhanced histone acetylation^22–24^. Changes in histone acetylation and downstream epigenetic/metabolic reprogramming confer adaptations to the harsh tumour milieu^24,25^.

Acidosis is a hallmark of exacerbated tumour growth that facilitates survival and growth in an acidic environment^26–28^. The hyperactive glycolytic metabolism in tumour cells directly accounts for the acidification of the tumour microenvironment. Therefore, it is imperative to investigate the mechanisms by which acetate reprograms histone acetylation and metabolic pathways to support tumour cell survival in the acidic tumour microenvironment. In this Article, by utilizing an integrated epigenomic, transcriptomic, metabolomic and proteomic approach, we elucidate a paracrine pathway regulating tumour–stromal metabolic crosstalk during PDAC progression and survival during acidosis. Targeting these metabolic nodes may offer therapeutic opportunities.

## Results

### PSC-secreted acetate regulates pancreatic cancer cell growth

**Fig. 1:**
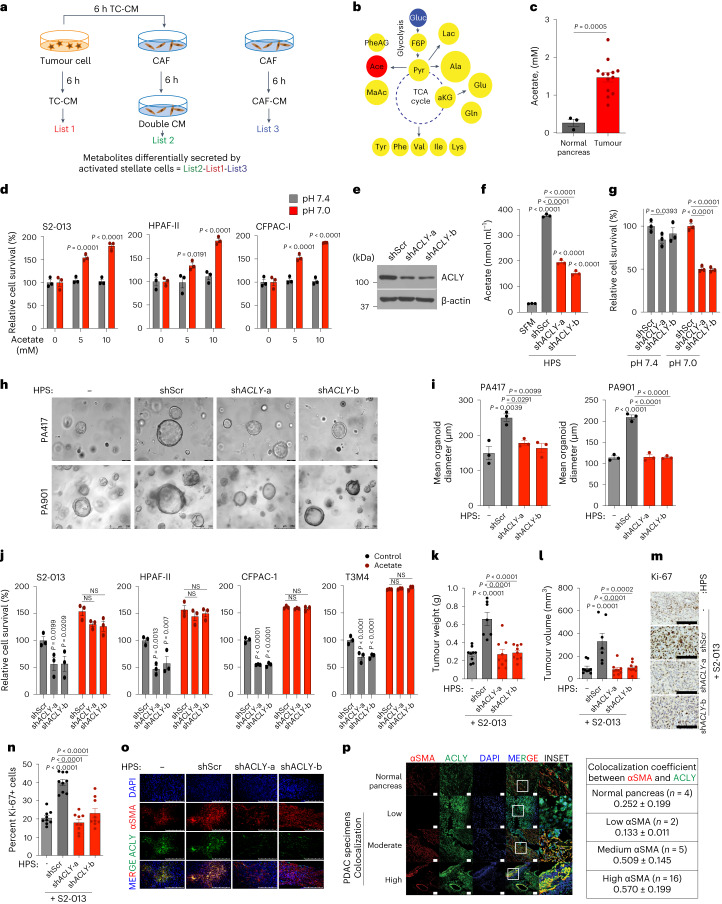
PSCs support cancer cell growth by the secretion of acetate under acidosis. **a**, Schematic of NMR-based metabolomics to identify PSC-derived metabolites. **b**, Metabolites identified in CAF-0911 CM: yellow, increased; blue, decreased; white, unchanged; acetate, red). **c**, Acetate levels in the interstitial fluid of human PDAC tumours (*n* = 13) and normal pancreas (*n* = 3). **d**, Relative PDAC cell survival with the indicated treatments (*n* = 3 in each group, from independent biological replicates). **e**, Immunoblot of ACLY in HPS cells. Representative image of two independent experiments. **f**, Acetate levels in shScr and sh*ACLY* HPS cell–CM compared to serum-free medium (SFM; *n* = 3 in each group, from independent biological replicates). **g**, Relative survival of S2-013 cells treated with CM derived from shScr and sh*ACLY* HPS cells in normal and low-pH conditions (*n* = 3 in each group, from independent biological replicates). **h**,**i**, Mean organoid diameters (**i**) and representative images (**h**) of PA417 and PA901 organoids cultured alone or in combination with shScr or sh*ACLY* HPS cells. Scale bars, 100 µm (PA417), 250 µm (PA901) (*n* = 3 in each group from independent biological replicates). **j**, Relative survival of PDAC cells treated with CM derived from shScr and sh*ACLY* HPS cells under low pH (pH 7.0) with 5 mM acetate (*n* = 3 in each group, from independent biological replicates). **k**–**o**, Tumour weights (**k**) and volumes (**l**), Ki-67 IHC staining images (**m**), Ki-67 quantification in three different fields from three tumours of each (**n**), and αSMA and ACLY co-expression by IHC (**o**) upon necropsy from mice implanted with S2-013 cells alone (*n* = 9) or co-implanted with shScr (*n* = 7) and sh*ACLY* (*n* = 9) HPS cells. Scale bars, 100 µm (**m**), 250 µm (**o**). Images are representative of three tumours of each group. **p**, Representative images and quantification of αSMA and ACLY colocalization coefficient in normal human pancreas and PDAC tumours. Scale bars, 50 µm. Image are representative of at least two different fields from *n* = 4 normal human pancreas and *n* = 23 tumours of each group. Unpaired two-tailed *t*-test, mean ± s.e.m. (**c**); two-way analysis of variance (ANOVA) with Šídák’s post-hoc test, mean ± s.e.m. (**d**); two-way ANOVA with Tukey’s post-hoc test, mean ± s.e.m. (**g**,**j**); one-way ANOVA with Bonferroni’s post-hoc test; mean ± s.e.m. (**f**,**i**,**k**,**l**,**n**). Source data

**Extended Data Fig. 1:**
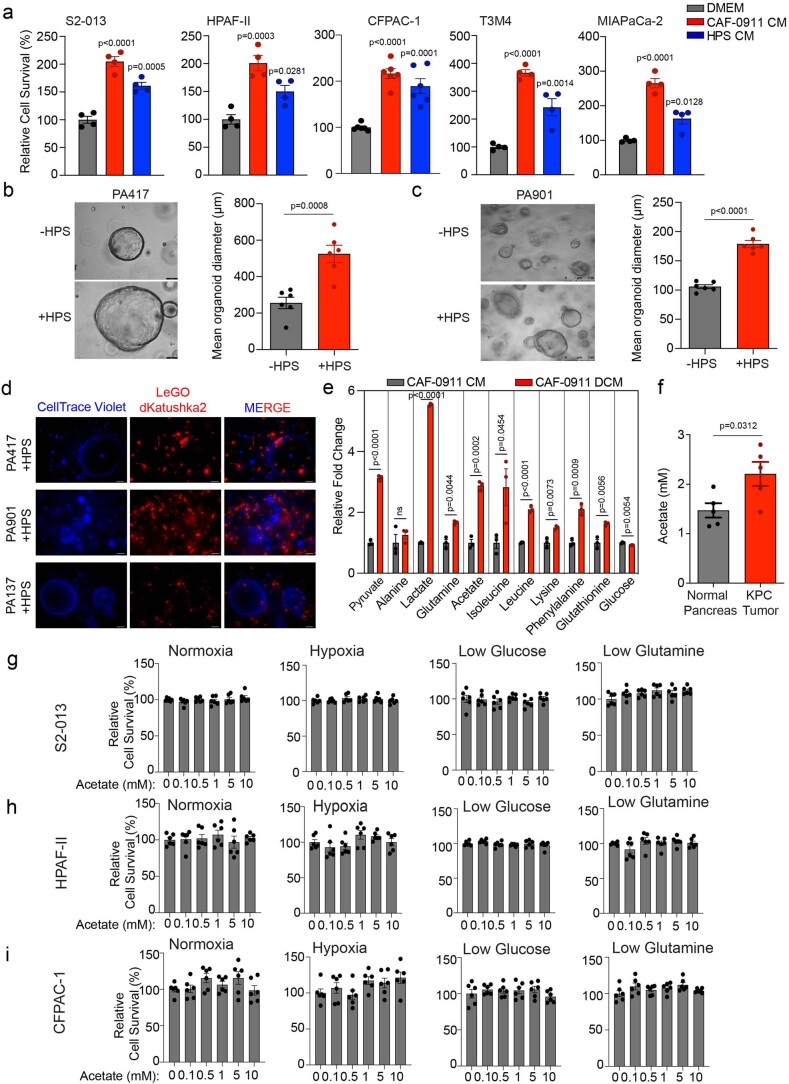
Additional characterization of pancreatic stellate cell-mediated support of cancer cell growth by secretion of acetate. **a**, Relative cell survival of pancreatic cancer cells (S2-013, HPAF-II, CFPAC-1, T3M4, and MIAPaCa-2) upon treatment with stellate cell (CAF-0911 and HPS)-derived conditioned medium (CM) (n = 4 for S2-013, n = 4 for HPAF-II, n = 6 for CFPAC-1, n = 4 for T3M4, n = 4 for MIAPaCa-2 in each group from independent biological replicates). **b,c**, Representative images and quantitation of diameter of PA417 (b) and PA901 (c) organoids cultured in the presence or absence of HPS cells. Scale bar = 100 µm (PA417), 250 µm (PA901) (n = 6 in each group from independent biological replicates). **d**, Immunofluorescent images showing the distribution of HPS cells stably expressing LeGO-dKatushka2 plasmid when co-cultured with multiple pancreatic cancer organoids (PA417, PA901, and PA137) labeled with CellTrace Violet dye for 5 days. Scale bar = 100 µm. Representative image of two independent experiments. **e**, Relative fold change in labeled metabolite levels in CM from CAF-0911 stellate cells (CM) or double conditioned medium (DCM) derived from CAF-0911 cells pre-conditioned with S2-013 tumor cell-conditioned media (n = 3 in each group from independent biological replicates). **f**, Levels of acetate in the interstitial fluid of pancreatic cancer tissues of KPC mice (female mice at 20-22 weeks of age) and control C57BL/6 J mice (n = 5 in each group from independent biological replicates). **g-i**, Relative cell survival of pancreatic cancer cells, S2-013 (g), HPAF-II (h), and CFPAC-1 (i) treated with increasing doses of acetate (0.1–10 mM) for 72 hrs cultured in normoxic, hypoxic, low glucose, and low glutamine conditions. The cell survival is normalized to the respective untreated controls (n = 6 in each group from independent biological replicates). One-way ANOVA with Tukey’s post-hoc test; mean ± s.e.m. (a,g,h,i); unpaired, two-tailed t-test; mean ± s.e.m. (b,c,e,f). Source data

To delineate the stromal cell-derived metabolites required for cancer cell survival and proliferation, we performed a conditioned-media (CM) transplant assay on cancer cells. The cancer cells showed increased growth upon treatment with CM from human CAF–CAF-0911 and human pancreatic stellate (HPS) cells (Extended Data Fig. 1a). Furthermore, pancreatic tumour-derived organoid lines PA417 and PA901 formed significantly larger organoids when co-cultured with HPS cells (Extended Data Fig. 1b,c). Additionally, we observed that the enhanced growth of the tumour organoids was not due to the incorporation of HPS cells within the organoid structure (Extended Data Fig. 1d). To identify the CAF-derived metabolites regulating cancer cell growth, we performed NMR-based metabolomics to investigate potential metabolites exchanged between tumour cells and CAFs. By performing ^13^C-glucose labelling, we observed that when stimulated with tumour-cell CM (TC-CM), CAFs secreted increased levels of lactate, amino acids such as alanine, lysine and glutamine, and other fatty-acid intermediates (Fig. 1a,b and Extended Data Fig. 1e). Of note, we also detected high levels of acetate (Extended Data Fig. 1e), a key metabolite routed to the central carbon metabolism under nutrient-depleted conditions. We also observed increased levels of acetate in the tumour-derived interstitial fluid of the *Kras*^*LSL.G12D*/+^; *p53*^*R172H*/+^; *Pdx1-Cre*^*tg*/+^ (KPC) mouse model of PDAC, which demonstrates a substantial amount of desmoplasia^29^ as compared to the pancreas from age-matched littermate controls (Extended Data Fig. 1f). Furthermore, the levels of acetate were also elevated in the tumour-derived interstitial fluid samples from PDAC patients in comparison to the healthy pancreas from organ donors (Fig. 1c). These data collectively demonstrate that CAFs play a critical role in the maintenance of acetate in the metabolic pool of the PDAC microenvironment. Notably, acetate treatment consistently increased the growth of pancreatic cancer cells (S2-013, HPAF-II and CFPAC-1) under low-pH conditions but not other physiological conditions (Fig. 1d and Extended Data Fig. 1g–i). In summary, our data suggest that stellate cell-secreted acetate regulates the growth of pancreatic cancer cells.

### *ACLY* loss in CAF abrogates acetate-induced PDAC tumour growth

**Extended Data Fig. 2:**
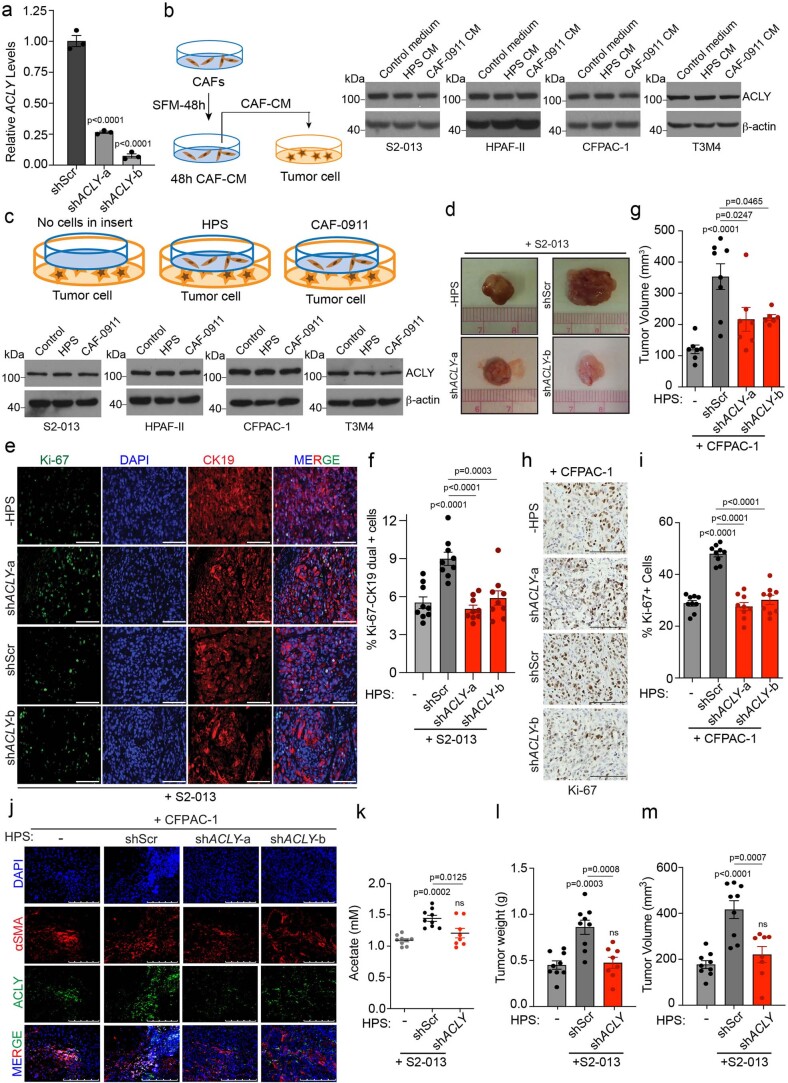
Pancreatic stellate cell ACLY contributes to acetate secretion and tumor burden. **a**, Relative mRNA levels of *ACLY* in human pancreatic stellate (HPS) cells expressing scrambled control (shScr) and sh*ACLY* plasmids (n = 3 in each group from independent biological replicates). **b**, Experiment scheme and immunoblots showing the expression of ACLY protein in PDAC cells upon treatment with CM-derived from HPS and CAF-0911 cells. Representative image of two independent experiments. **c**, Experiment scheme and immunoblots showing the expression of ACLY protein in S2-013, HPAF-II, CFPAC-1, and T3M4 cells upon co-culture with HPS and CAF-0911 cells for 24 hrs. Representative image of two independent experiments. **d**, Representative images of tumors excised from athymic-nude mice implanted with S2-013 cells alone (n = 9) or co-implanted with control (shScr; n = 7) and *ACLY* knockdown (sh*ACLY*; target a, n = 9; target b, n = 9) HPS cells. **e,f**, Representative IHC images for Ki-67 staining, nuclear staining (DAPI), cytokeratin 19 (epithelial/tumor cells), and merge in tumor sections from athymic-nude mice implanted with S2-013 cells alone or co-implanted with shScr or sh*ACLY* HPS cells (along with the quantitation of percent Ki-67 and CK19 dual positive cells (f). Scale bar = 100 µm. Ki-67 and CK19 positive cells were counted manually in three different fields from 3 tumor sections of each group (n = 9 in each group). **g**, Tumor volumes, upon necropsy of athymic-nude mice implanted with CFPAC-1 cells alone (n = 7) or co-implanted with shScr (n = 8) and sh*ACLY* (sh*ACLY*-a, n = 7; sh*ACLY*-b, n = 6) HPS cells. **h,i**, Representative IHC images for Ki-67 staining (h) in tumor sections from athymic-nude mice implanted with CFPAC-1 cells alone or co-implanted with shScr or sh*ACLY* HPS cells along with the quantitation of percent positive cells (i). Scale bar = 100 µm. Ki-67 positive cells were counted manually in three different fields from 3 tumor sections of each group (n = 9 in each group). **j**, Representative immunofluorescent images showing co-expression of αSMA and ACLY proteins in tumor sections from athymic-nude mice implanted with CFPAC-1 cells alone or co-implanted with shScr or sh*ACLY* HPS cells. Scale bar = 250 µm. **k**, Levels of acetate in the interstitial fluid of tumor tissues from athymic-nude mice implanted with S2-013 cells alone (-) or co-implanted with shScr or sh*ACLY* HPS cells. **l,m**, Tumor weights (l) and tumor volumes (m), upon necropsy from athymic-nude mice implanted with S2-013 cells alone or co-implanted with shScr or sh*ACLY* HPS cells. For k,l,m, n = 9 (S2-013 alone), 9 (co-implanted with shScr HPS), 8 (co-implanted with sh*ACLY* HPS) mice. One-way ANOVA with Bonferroni’s post-hoc test; mean ± s.e.m. (a,f,g,i,k,l,m). Source data

Next, we examined whether targeting CAF-mediated acetate production can abolish stellate cell support of tumour-cell survival at low pH. A recent study has reported that ACLY deficiency confers a glucose-to-acetate switch, in which ACLY-deficient mouse embryonic fibroblasts (MEFs) upregulate ACSS2 and increase cellular reliance on acetate for viability, histone acetylation and lipid synthesis^30^. We thus hypothesized that *ACLY*-deficient fibroblasts would not secrete acetate and will instead take up and consume acetate to supply their own acetyl-CoA pools and result in decreased secretion of acetate. To test this hypothesis, we knocked down *ACLY*, a cytoplasmic enzyme critical for converting citrate into the acetate-precursor acetyl CoA^31^, in stellate cells (Extended Data Fig. 2a and Fig. 1e). We observed that targeting ACLY significantly diminishes acetate secretion into the media (Fig. 1f). The knockdown of *ACLY* in PSCs led to a significant reduction in the survival of cancer cells under low pH upon CM transplantation (Fig. 1g). We observed similar reductions in the average organoid size in PA417 and PA901 organoids when co-cultured with *ACLY* knockdown HPS cells, as compared to the scrambled controls (Fig. 1h,i). Of note, because of the CM from growth factor-expressing cells, our organoid medium pH was close to 7.0. Treatment of cancer cells with CAF-derived CM or co-culture with CAFs, separated by cell-culture inserts, did not alter the ACLY expression in cancer cells (Extended Data Fig. 2b,c). To address the specificity of the change in acetate being the main driver of the observed phenotype, we performed rescue experiments wherein adding acetate to CM from *ACLY* knockdown CAFs enhanced the survival of the pancreatic cancer cells in the in vitro tumour cell survival assay (Fig. 1j). However, *ACLY* knockdown in HPS cells abolished the stellate cell-mediated increase in tumour burden and proliferative tumour cell population from co-implanted S2-013 cells (Fig. 1k–o and Extended Data Fig. 2d–f). These findings were further replicated in another in vivo co-implantation model of WT or *ACLY* knockdown HPS cells with CFPAC-1 PDAC cells (Extended Data Fig. 2g–j). Notably, we observed a significant increase in the levels of acetate in the interstitial fluid upon co-implantation with stellate cells that was abrogated by *ACLY* knockdown in stellate cells (Extended Data Fig. 2k–m). Furthermore, we observed increased colocalization of ACLY protein and the CAF marker, smooth muscle actin (αSMA), in primary pancreatic tumours compared to the healthy pancreas (Fig. 1p). In conclusion, ACLY-mediated secretion of acetate is critical for the growth of pancreatic tumours.

### Diversity in CAF subtypes does not impact acetate secretion

**Extended Data Fig. 3:**
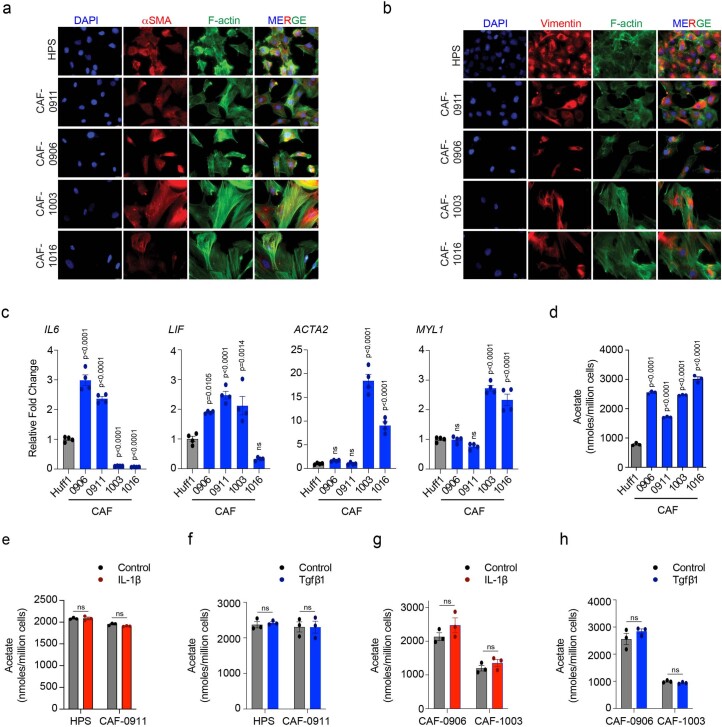
Characterizing acetate secretion in different CAF subtypes. **a,b** Representative images showing expression of αSMA (a) or Vimentin (b) by immunofluorescence staining in HPS and CAF-0911 cell lines and primary CAFs (CAF-0906, CAF-1003, and CAF-1016), as imaged by staining cells with fluorescently-tagged phalloidin for F-actin. Nuclei were stained with DAPI. Scale bar = 20 µm. **c**, Relative mRNA expression of iCAF markers (*IL6, LIF*) and myCAF markers (*ACTA2, MYL1*) in Huff1 (human foreskin fibroblast cell line; used as a control), CAF-0911, CAF-0906, CAF-1003, and CAF-1016 cells. The gene expression is normalized to Huff1 cells (n = 4 in each group from independent biological replicates). **d**, Acetate levels in Huff1 control cells and CAF-0911, CAF-0906, CAF-1003, and CAF-1016 cancer-associated fibroblast cells (n = 3 in each group from independent biological replicates). **e,f**, Acetate levels in HPS and CAF-0911 cells upon treatment with IL-1β (e) or Tgfβ1 (f) for 48 hrs (n = 3 in each group from independent biological replicates). **g,h**, Acetate estimation in CAF-0906 and CAF-1003 cells upon treatment with IL-1β (g) or Tgfβ1 (h) for 48 hrs. (n = 3 in each group from independent biological replicates). One-way ANOVA with Bonferroni’s post-hoc test; mean ± s.e.m. (c,d); unpaired, two-tailed t-test; mean ± s.e.m. (e,f,g,h). Source data

To identify which CAF subtype contributes to acetate production, we collected and characterized CAFs from different patients and categorized them as myofibroblastic (myCAFs; high αSMA; induced by transforming growth factor-β1 (TGF-β1)) or inflammatory (iCAFs; low αSMA but higher cytokines and chemokines; induced by interleukin-1β (IL-1β)) (Extended Data Fig. 3a–c). Estimation of acetate secreted by CAF-0911 and the primary CAFs (CAF-0906, CAF-1003 and CAF-1016) indicated that the ability to secrete acetate was independent of CAF subtype, at least in the culture conditions (Extended Data Fig. 3d). Furthermore, IL-1β and TGF-β1 treatments failed to show any differences in the levels of acetate secreted by myCAF or iCAF subtypes of CAFs (Extended Data Fig. 3e–h). Taken together, our studies indicate that the secretion of acetate by CAFs is subtype independent.

### Acetate alters histone acetylation under acidosis via ACSS2

**Fig. 2:**
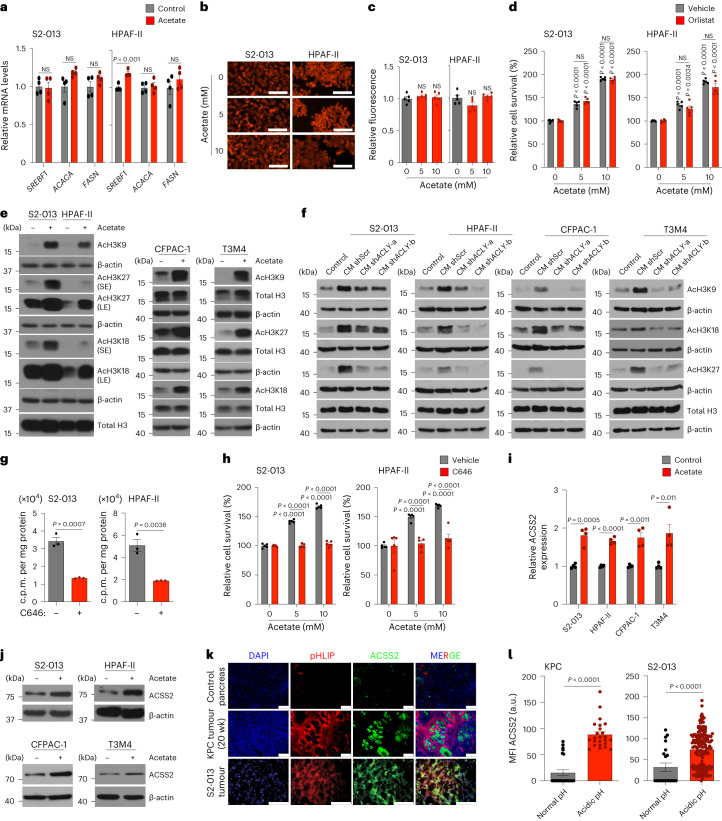
Acetate-mediated histone acetylation and ACSS2 activation control pancreatic cancer growth under acidosis. **a**, Relative expression of *SREBF1*, *ACACA* and *FASN* genes in PDAC cells treated with 5 mM acetate under acidosis (*n* = 4 in each group, from independent biological replicates). **b**,**c**, Representative fluorescent images (**b**) and quantification (**c**) of Nile red staining in PDAC cells treated with 5 and 10 mM acetate. Scale bars, 100 µm (*n* = 5 in each group, from independent biological replicates). **d**, Relative survival of PDAC cells treated with vehicle or 100 µM orlistat, without or with acetate (*n* = 5 in each group, from independent biological replicates). **e**, Immunoblots of acetylated histones in PDAC cells treated with acetate (SE, short exposure; LE, long exposure). Images are representative of two independent experiments. **f**, Immunoblots of acetylated histones in PDAC cells treated with CM from shScr, sh*ACLY*-a and sh*ACLY*-b HPS cells. Images are representative of two independent experiments. **g**, ^14^C-acetate incorporation (counts per minute (c.p.m.)) in total histones extracted from S2-013 and HPAF-II cells treated with acetate in the absence and presence of 10 µM C646 for 6 h under acidosis (*n* = 3 in each group, from independent biological replicates). **h**, Relative survival of PDAC cells treated with 10 µM C646 inhibitor, without or with acetate (*n* = 5 in each group, from independent biological replicates). **i**, Relative mRNA expression of *ACSS2* in PDAC cells treated with acetate under acidosis (*n* = 4 in each group, from independent biological replicates). **j**, Immunoblot of ACSS2 in PDAC cells upon treatment with acetate for 24 h under acidosis. Representative of two independent experiments. **k**,**l**, Representative immunofluorescent images (**k**) and quantitation (**l**) showing expression of ACSS2 protein in acidic tumour regions, as imaged by staining with pHLIP in tumour sections from 20-week-old KPC mice, and AT-nude mice orthotopically implanted with S2-013 cancer cells. Scale bars, 100 µm (KPC, normal (*n* = 24) and acidic (*n* = 24) pH; S2-013, normal (*n* = 21) and acidic (*n* = 168) pH). Unpaired two-tailed *t*-test, mean ± s.e.m. (**a**,**g**,**i**,**l**); one-way ANOVA with Bonferroni’s post-hoc test, mean ± s.e.m. (**c**); two-way ANOVA with Tukey’s post-hoc test, mean ± s.e.m. (**d**,**h**). Source data

**Extended Data Fig. 4:**
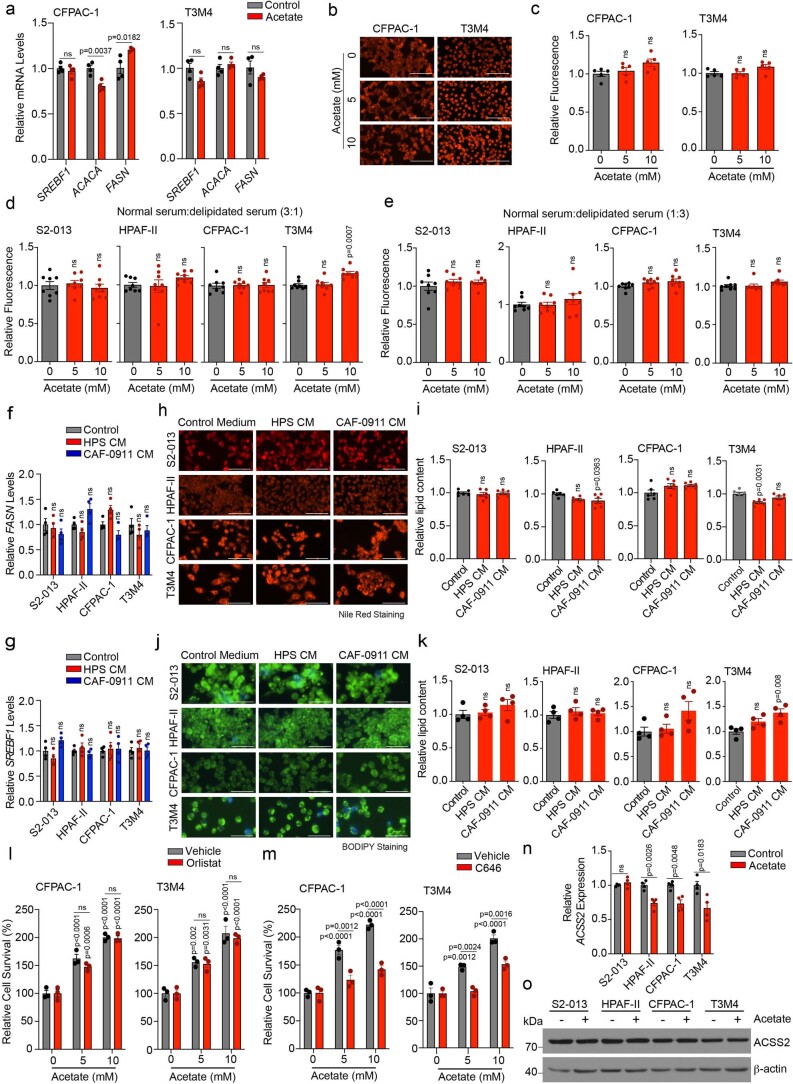
Effect of acetate treatment on lipid biosynthesis, histone acetylation, and ACSS2 expression in pancreatic cancer cells under acidosis. **a**, Relative mRNA expression of *SREBF1*, *ACACA* and *FASN* genes in CFPAC-1 and T3M4 cells upon treatment with 5 mM acetate for 12 hrs (n = 4 in each group from independent biological replicates). **b,c**, Representative fluorescent images (b) showing Nile red staining in CFPAC-1 and T3M4 cells upon acetate treatment for 96 hrs. The bar charts show relative fluorescence levels of Nile red staining (c). Total lipid fluorescence is normalized to the cell count. Scale bar = 100 µm. (n = 5 in each group from independent biological replicates). **d,e**, Relative quantification of Nile red fluorescence in PDAC cells treated with acetate for 96 hrs. The cells were cultured in a DMEM medium containing normal and delipidated FBS in a 3:1 (d) or 1:3 (e) ratio. The total Nile red fluorescence was normalized to the cell count (n = 8 in each group from independent biological replicates). **f,g**, Relative mRNA expression of *FASN* (f) and *SREBF1* (g) genes in PDAC cell lines upon treatment with CM from HPS and CAF-0911 cells for 12 hrs (n = 4 in each group from independent biological replicates). **h-k**, Representative fluorescent images showing the Nile red (h) or BODIPY (j) staining in PDAC cell lines upon treatment with CM from HPS and CAF-0911 cells for 96 hrs. Scale bar = 100 µm. The bar charts show relative fluorescence levels of Nile red (i) or BODIPY (k) staining. Total lipid fluorescence is normalized to the cell count (n = 6 in each group for ‘i’ and n = 4 in each group for ‘k’ from independent biological replicates). **l**, Relative survival of CFPAC-1 and T3M4 cells upon treatment with control or 100 µM orlistat, without or with acetate (n = 3 in each group from independent biological replicates). **m**, Relative survival of CFPAC-1 and T3M4 cells upon treatment with 10 µM C646 inhibitor, without or with acetate (n = 3 in each group from independent biological replicates). **n**, Relative mRNA expression of *ACSS2* in S2-013, HPAF-II, CFPAC-1, and T3M4 cells upon treatment with 5 mM acetate for 12 hrs under normal pH conditions (n = 4 in each group from independent biological replicates). **o**, Representative immunoblots showing expression of ACSS2 in S2-013, HPAF-II, CFPAC-1, and T3M4 cells upon treatment with 5 mM acetate under normal pH conditions. Representative image of two independent experiments. Unpaired, two-tailed t-test; mean ± s.e.m. (a,n); one-way ANOVA with Bonferroni’s post-hoc test; mean ± s.e.m. (c,d,e,f,g,i,k,); two-way ANOVA with Tukey’s multiple comparisons test; mean ± s.e.m.(l,m). Source data

Previous studies have demonstrated the critical role of acetate in lipid biomass production under hypoxia, a condition extensively found in the tumour microenvironment^25^. However, analysis of the lipid biosynthetic pathway genes *SREBF1*, *FASN* and *ACACA* revealed no significant difference in multiple PDAC cell lines upon acetate treatment (Fig. 2a and Extended Data Fig. 4a). Similarly, staining and quantitative analysis of total lipid content in the pancreatic cancer cells did not show any increase in intracellular lipid content upon acetate treatment of cells cultured at low pH (Fig. 2b,c and Extended Data Fig. 4b,c). Furthermore, acetate treatment failed to alter the cellular lipid content at low pH, even under conditions of decreased lipid levels in the culture medium (Extended Data Fig. 4d,e). Similar to the acetate treatment, CM treatment did not modulate either the expression of lipid biosynthetic genes or total lipid content under acidosis (Extended Data Fig. 4f–k). Accordingly, pharmacological inhibition of lipid biosynthesis with orlistat, an inhibitor of the thioesterase domain of fatty-acid synthase^32^, could not abolish acetate-induced survival of cancer cells under acidosis (Fig. 2d and Extended Data Fig. 4l). A recent study also noted that ACSS2 is highly expressed and prominently nuclear in murine pancreatic tumours^33^. To comprehensively define acetate-dependent regulation of chromatin modifications, we analysed histone modifications upon acetate supplementation of pancreatic cancer cells cultured at low-pH conditions. Acetate treatment enhanced the acetylation of several histone marks, including H3K9, H3K18 and H3K27, in multiple PDAC cell lines (Fig. 2e). Furthermore, we observed an increase in the acetylation of H3K9, H3K18 and H3K27 marks upon treatment with wild-type stellate cell-derived conditioned media; however, conditioned media from sh*ACLY* stellate cells failed to show any increase in tumour cell histone acetylation (Fig. 2f). Treatment of cancer cells with a histone acetyl transferase (HAT) inhibitor, C646, decreased the flux of ^14^C-acetate into the total histone acetylation (Fig. 2g). Furthermore, treatment with C646 abrogated acetate-induced survival of cancer cells at low pH, demonstrating that acetate regulates cancer cell survival via remodelling of histone modifications (Fig. 2h and Extended Data Fig. 4m). We also observed an increase in messenger RNA (mRNA) expression and the protein levels of acetate-synthesizing enzyme acetyl-CoA synthetase short-chain family member 2 (ACSS2, a nucleocytosolic enzyme) in acetate-treated cancer cells under acidosis but not under normal pH (Fig. 2i,j and Extended Data Fig. 4n–o). Next, we investigated whether ACSS2 localizes to the low-pH regions of tumours. We utilized pH low insertion peptides (pHLIP), which are water-soluble, inserting them into the plasma membrane of cells in acidic tumour regions, to image low-pH regions^34^. We observed an increase in the expression of ACSS2 in acidic regions of the tumour compared to tumour areas with normal pH (Fig. 2k,l). Taken together, our data suggest that acetyl-CoA metabolism in pancreatic cancer cells regulates histone acetylation levels and is functionally critical for the survival of pancreatic cancer cells under low pH.

### ACSS2 induction regulates PDAC cell survival under acidosis

**Fig. 3:**
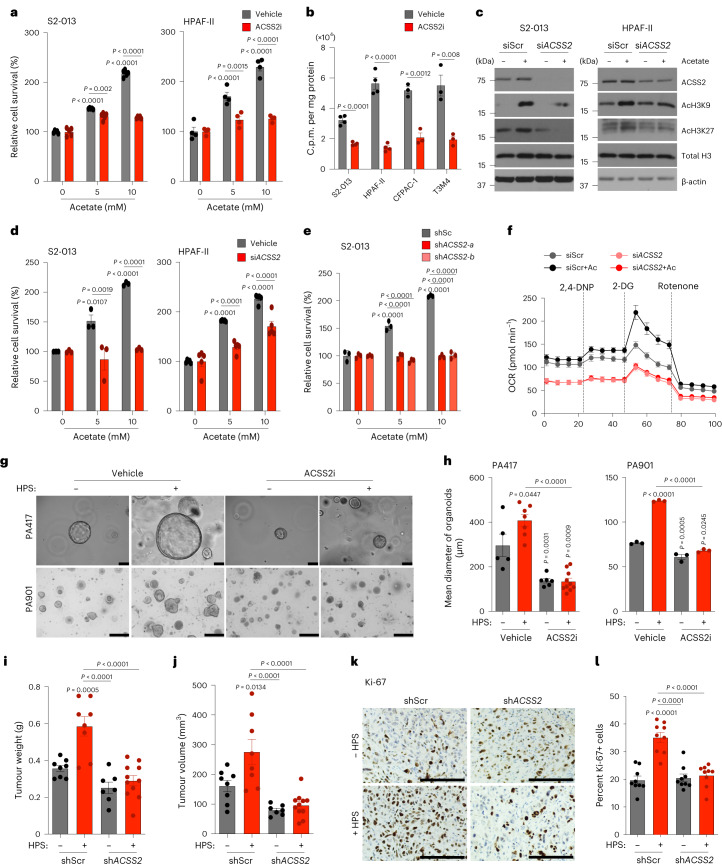
ACSS2 expression in PDAC cells regulates acetate-mediated growth of pancreatic cancer cells in vitro and in vivo under acidosis. **a**, Relative survival of PDAC cells upon treatment with 20 µM ACSS2 inhibitor, without or with acetate (*n* = 5 (S2-013), *n* = 4 (HPAF-II) in each group, from independent biological replicates). **b**, ^14^C-acetate incorporation in total histones extracted from PDAC cells treated with 5 mM acetate in the presence of vehicle control or 20 µM ACSS2 inhibitor for 6 h (*n* = 4 in each group (S2-013, HPAF-II), *n* = 3 (CFPAC-1, T3M4), from independent biological replicates). **c**, Representative immunoblots showing the levels of ACSS2, acetylated histones and total H3 protein in scrambled control (siScr) and *ACSS2* knockdown (siACSS2) S2-013 and HPAF-II cells upon treatment with 5 mM acetate. Images are representative of two independent experiments. **d**, Relative survival of siScr and si*ACSS2* PDAC cells cultured upon acetate treatment (*n* = 3 for S2-013, *n* = 5 for HPAF-II in each group, from independent biological replicates). **e**, Relative survival of control (shScr) and *ACSS2* knockdown (shACSS2) S2-013 cells cultured without or with acetate (*n* = 3 in each group, from independent biological replicates). **f**, Relative oxygen consumption rate (OCR) in siScr and si*ACSS2* S2-013 cells upon acetate treatment (*n* = 8 in each group, from independent biological replicates). 2,4-DNP, 2,4-dinitrophenylhydrazine; 2-DG, 2-deoxyglucose. **g**,**h**, Representative images (**g**) and quantification of mean organoid diameter (**h**) of PA417 and PA901 organoids cultured −/+ HPS cells upon treatment with 50 µM ACSS2 inhibitor. Scale bars, 100 µm. PA417, *n* = 5, 7, 6, 10); PA901, *n* = 3 in each group, from independent biological replicates. **i**,**j**, Tumour weights (**i**) and volumes (**j**), upon necropsy for mice implanted with shScr or sh*ACSS2* S2-013 cells alone or co-implanted with HPS cells (shScr − HPS (*n* = 8), shScr + HPS (*n* = 8), sh*ACSS2* − HPS (*n* = 7), sh*ACSS2* + HPS (*n* = 11)). **k**,**l**, Representative IHC images for Ki-67 staining (**k**) in tumour sections from mice implanted with shScr or sh*ACSS2* S2-013 cells alone or co-implanted with HPS cells, along with quantitation (**l**) counted in three different fields from three tumour sections of each group (*n* = 9 in each group). Scale bars, 100 µm. Unpaired, two-tailed *t*-test, mean ± s.e.m. (**b**); two-way ANOVA with Tukey’s post-hoc test, mean ± s.e.m. (**a**,**d**,**e**); one-way ANOVA with Bonferroni’s post-hoc test, mean ± s.e.m. (**h**–**j**,**l**). Source data

**Extended Data Fig. 5:**
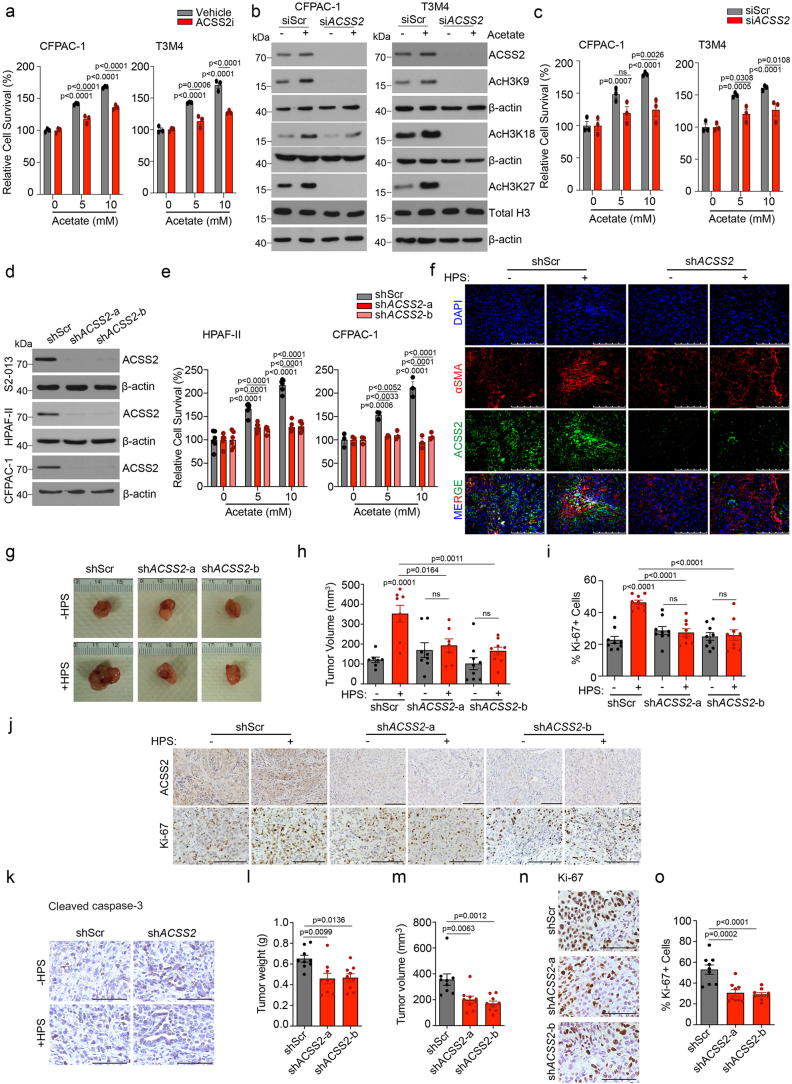
Role of ACSS2 in acetate-mediated epigenetic reprogramming, survival under acidosis, and tumor burden. **a**, Relative survival of CFPAC-1 and T3M4 cells upon treatment with 20 µM ACSS2 inhibitor, without or with acetate (n = 3 in each group from independent biological replicates). **b**, Representative immunoblots (of two independent experiments) showing the levels of ACSS2, acetylated histones, and total H3 protein in scrambled control (siScr) and ACSS2 knockdown (siACSS2) CFPAC-1 and T3M4 cells upon treatment with 5 mM acetate. **c**, Relative survival of siScr and siACSS2 CFPAC-1 and T3M4 cells in the presence and absence of acetate (n = 3 in each group from independent biological replicates). **d**, Immunoblots (representative of two independent experiments) showing the levels of ACSS2 protein in control (shScr) and ACSS2 knockdown (shACSS2- a and shACSS2-b) S2-013, HPAF-II, and CFPAC-1 cells under acidosis. **e**, Relative survival of control and ACSS2 knockdown HPAF-II and CFPAC-1 cells in the presence, and absence of acetate (n = 6 for HPAF-II and n = 3 for CFPAC-1 in each group from independent biological replicates). **f**, Representative immunofluorescent images showing co-expression of stellate cell marker (αSMA) and ACSS2 in tumor sections from athymic-nude mice implanted with shScr or shACSS2 S2-013 cells alone or co-implanted with HPS cells. Scale bar = 250 µm. **g**, Representative images of tumors excised from athymic-nude mice implanted with shScr or shACSS2 CFPAC-1 cells alone or co-implanted with HPS cells. **h**, Tumor volumes, upon necropsy, of athymic-nude mice implanted with control and ACSS2 knockdown CFPAC-1 cells alone or co-implanted with HPS cells (n = 7, 8, 8, 7, 9, 9 mice in indicated groups). **i,j**, Representative IHC images for ACSS2 and Ki-67 staining (j) in tumor sections from athymic-nude implanted with shScr or shACSS2 CFPAC-1 cells alone or co-implanted with HPS cells along with the quantitation of percent positive Ki-67 cells (i). Scale bar = 100 µm. Ki-67 positive cells were counted manually in three different fields from 3 tumor sections of each group (n = 9 in each group). **k**, Representative IHC staining for cleaved caspase-3 protein in tumor sections from athymic-nude mice implanted with shScr or shACSS2 S2-013 cells alone or co-implanted with HPS cells. Scale bar = 100 µm. **l,m**, Tumor weights (l) and tumor volumes (m), upon necropsy for athymic-nude mice implanted with shScr or shACSS2 (shACSS2-a and shACSS2-b) S2-013 cells. All groups were compared to shScr cohort. (n = 9 mice in each group). **n,o**, Representative IHC images for Ki-67 staining (n) in tumor sections from athymic-nude mice implanted with shScr or shACSS2 S2-013 cells along with the quantitation of percent Ki-67 positive cells (o). Scale bar = 100 µm. Ki-67 positive cells were counted manually in three different fields from 3 tumor sections of each group (n = 9 in each group). Two-way ANOVA with Tukey’s post-hoc test; mean ± s.e.m. (a,c,e); one-way ANOVA with Bonferroni’s post-hoc test; mean ± s.e.m. (h,i,l,m,o). Source data

**Extended Data Fig. 6:**
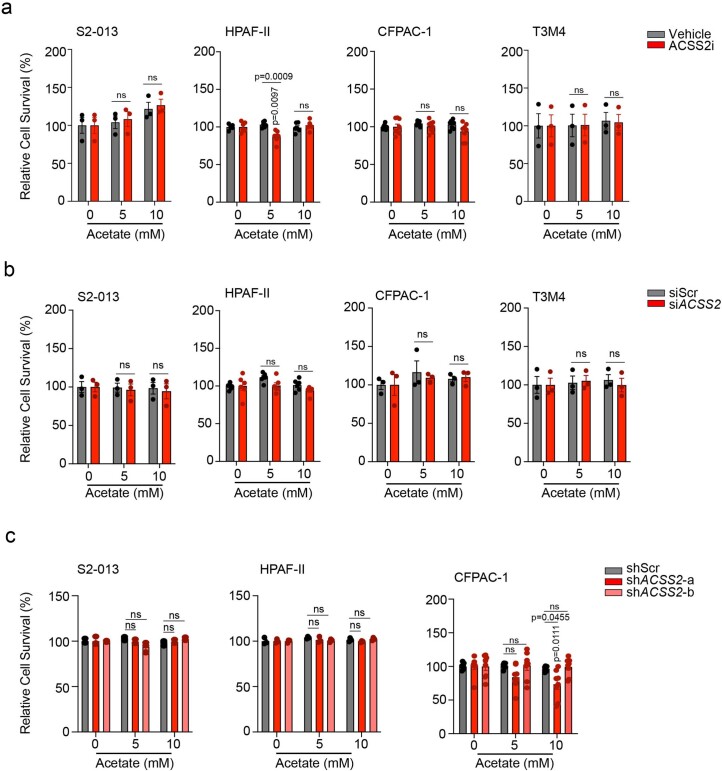
Additional characterization of the role of ACSS2 in tumor cell survival under normal pH. **a**, Relative survival of S2-013, HPAF-II, CFPAC-1, and T3M4 cells upon treatment with 20 µM ACSS2 inhibitor, without and with acetate, for 72 hrs under normal pH conditions. The cell survival is normalized to the respective untreated controls. (n = 3 for S2-013, n = 6 for HPAF-II, n = 8 for CFPAC-1, n = 3 for T3M4 in each group from independent biological replicates). **b**, The relative survival of scrambled control (siScr) and *ACSS2* knockdown (si*ACSS2*) S2-013, HPAF-II, CFPAC-1, and T3M4 cells cultured in the presence and absence of acetate for 72 hrs under normal pH conditions. The cell survival is normalized to the respective untreated controls. (n = 3 for S2-013, n = 6 for HPAF-II, n = 3 for CFPAC-1, n = 3 for T3M4 in each group from independent biological replicates). **c**, The relative survival of scrambled control (shScr) and *ACSS2* knockdowns (sh*ACSS2*-a and sh*ACSS2*-b) S2-013, HPAF-II, and CFPAC-1 cells cultured in the presence and absence of acetate for 72 hrs under normal pH conditions. The cell survival is normalized to the respective untreated controls. (n = 3 for S2-013, n = 3 for HPAF-II, n = 8 for CFPAC-1 in each group from independent biological replicates). Two-way ANOVA with Tukey’s post-hoc test; mean ± s.e.m. (a,b,c). Source data

We next tested whether ACSS2 is critical for acetate-induced cancer cell survival under low-pH conditions. As expected, ACSS2 inhibition abrogated acetate-mediated survival of cancer cells under acidosis (Fig. 3a and Extended Data Fig. 5a). Furthermore, the treatment of cancer cells with ACSS2 inhibitor under acidosis reduced the flux of ^14^C-acetate into the total histone proteins in multiple PDAC cell lines (Fig. 3b). The depletion of ACSS2 using small interfering RNA (siRNA) led to a reduction in acetate-induced acetylation of H3K9 and H3K27 proteins (Fig. 3c and Extended Data Fig. 5b) and abrogation of acetate-induced survival of PDAC cells under low-pH conditions (Fig. 3d,e and Extended Data Fig. 5c–e). Furthermore, *ACSS2* depletion abrogated the acetate-mediated increase in oxygen consumption rates in S2-013 cells (Fig. 3f) and HPS-induced growth of PA417 and PA901 organoids in co-cultures (Fig. 3g,h). Similarly, *ACSS2* knockdown in tumour cells abrogated the stellate cell-induced increase in tumour burden (Fig. 3i,j and Extended Data Fig. 5f–h) and tumour cell proliferation rates (Fig. 3k,l and Extended Data Fig. 5i,j), without impacting apoptosis (Extended Data Fig. 5k), in athymic (AT) nude mice orthotopically co-implanted with S2-013/CFPAC-1 tumour cells and HPS cells. To validate the critical role of ACSS2 in PDAC baseline tumours without CAFs, we assessed the growth of orthotopic tumours depleted for ACSS2 in the in vivo model without CAFs and let the tumours grow longer until the mice reached the euthanasia criteria (Extended Data Fig. 5l–o). We observed a significant reduction in the growth of tumours without CAF co-implantation when *ACSS2* was knocked down in cancer cells, compared to the scrambled control tumours. These results support the baseline conclusion that ACSS2 is important for PDAC tumour growth and is induced further by co-implantation with CAFs. The observed effects of depletion of *ACSS2* on acetate-mediated effects on pancreatic cancer survival were specific to acidic conditions in vitro (Extended Data Fig. 6a–c). In conclusion, our data demonstrate the critical role of the *ACSS2* gene in acetate-induced tumour cell survival under low pH and stellate cell-induced tumour burden in vivo.

### Acetate regulates SAT1 by enhancing chromatin accessibility

**Fig. 4:**
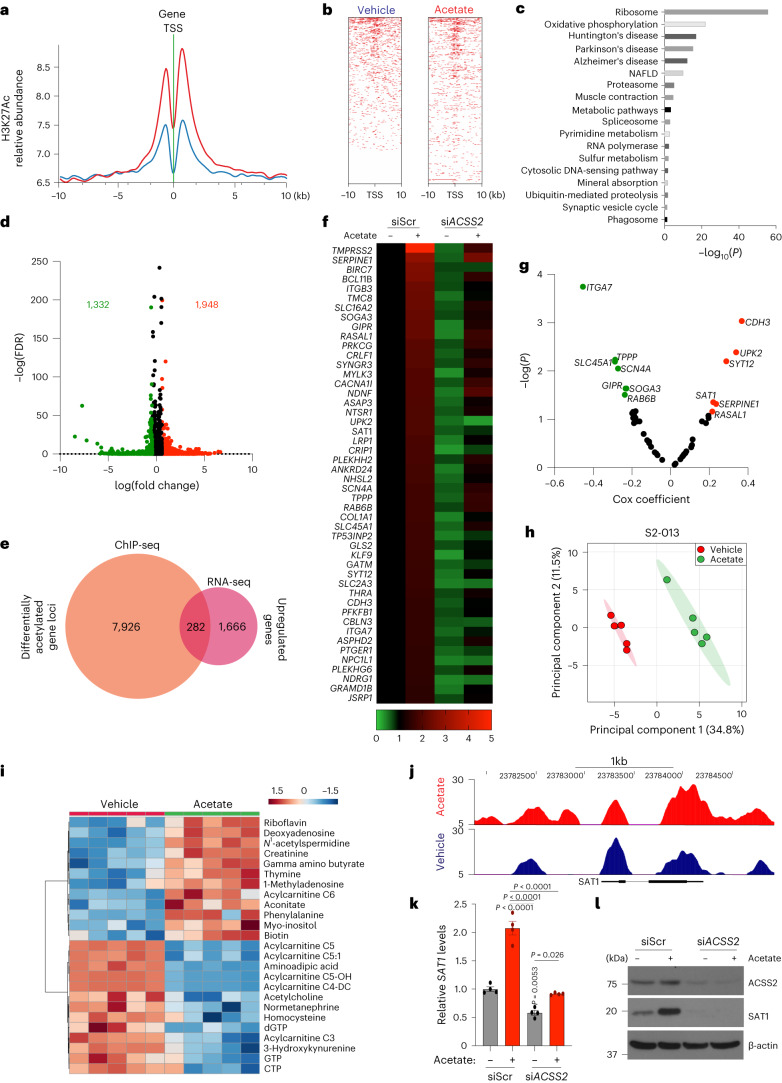
H3K27Ac ChIP–seq and RNA–seq analyses identify *SAT1* as a critical regulator of acetate-mediated effects. **a**, Distribution of H3K27 acetylation (H3K27Ac) ChIP–seq reads from vehicle-treated (blue) and acetate- treated (red) S2-013 cells within ±10 kb of the TSS. **b**, Density of ChIP–seq reads for H3K27ac ±10 kb from the TSS in vehicle- and acetate-treated S2-013 cells. **c**, DAVID-based pathway enrichment analysis of differentially H3K27-acetylated gene promoters in vehicle and acetate-treated S2-013 cell line (statistical analysis using Fisher’s exact test). **d**, Volcano plot depicting differentially regulated genes (1.5-fold change cutoff) in S2-013 cells upon acetate treatment. **e**, Venn diagram of ChIP–seq and RNA–seq data showing 282 genes that are upregulated in RNA–seq data with differentially acetylated H3K27 in their gene promoters. **f**, Heatmap showing 48 differentially regulated genes upregulated by acetate in an ACSS2-dependent manner. The colour bar shows the ratio of fragments per kilobase of transcript per million mapped reads (FPKM) values of siScr, siScr + acetate, si*ACSS2*, or si*ACSS2* + acetate to that of siScr. **g**, Volcano plot showing the Cox coefficient and −log(*P* value) of the 48 regulated genes extracted from the OncoLnc database for survival of patients with PDAC. Prospective oncogenes and tumour suppressors are shown in red and green, respectively. **h**, Principal component analysis (PCA) plot of S2-013 cells treated with acetate under acidosis relative to untreated cells as determined by LC–MS/MS-based metabolomics (*n* = 5 biological replicates per group). **i**, Heatmap of top 25 altered metabolites from S2-013 cells treated with acetate under acidosis and displayed with row *Z*-score normalization, as determined by LC–MS/MS-based metabolomics (*n* = 5 biological replicates per group). **j**, H3K27ac at putative enhancer regions proximal to the *SAT1* gene. The *y* axis shows reads per bin per million. **k**, Relative mRNA levels of the *SAT1* gene in siScr and si*ACSS2* S2-013 cells in the presence or absence of acetate for 24 h under acidosis (*n* = 4 in each group, from independent biological replicates; one-way ANOVA with Bonferroni’s post-hoc test, mean ± s.e.m.). **l**, Relative levels of SAT1 and ACSS2 proteins in scrambled control (siScr) and *ACSS2* knockdown (si*ACSS2*) S2-013 cells in the presence and absence of 5 mM acetate for 24 h under acidosis. Immunoblots are representative of two independent experiments. Source data

**Extended Data Fig. 7:**
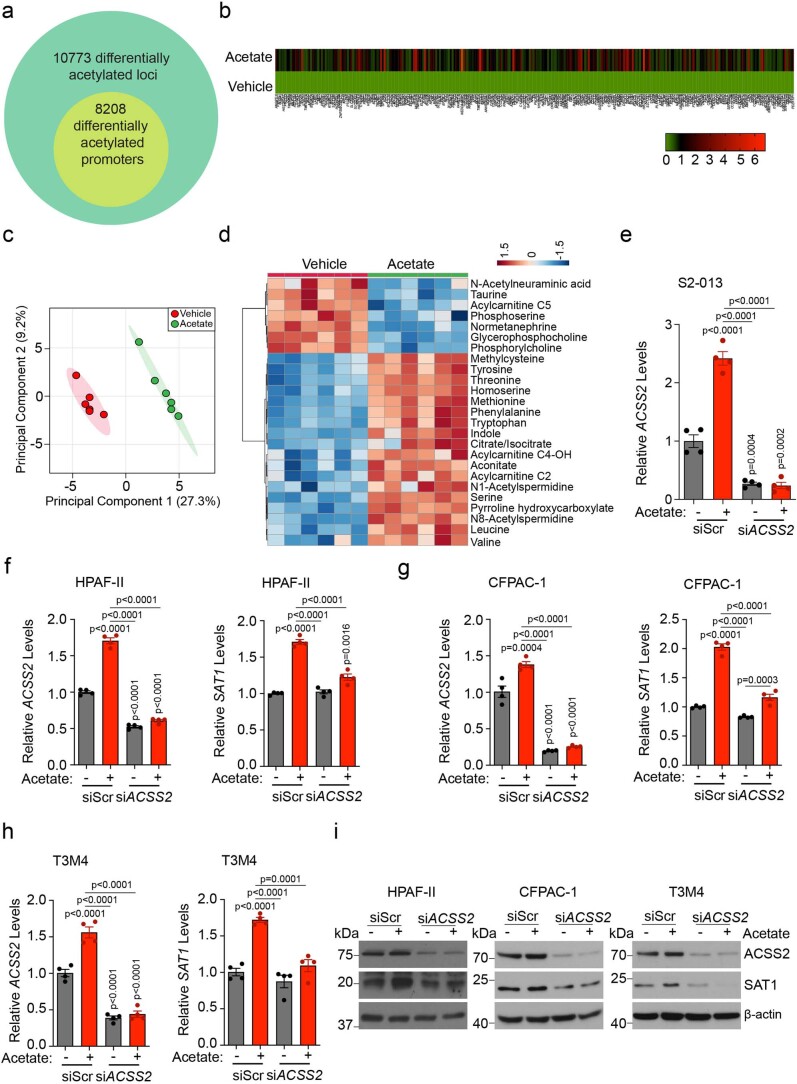
Acetate-mediated regulation of chromatin accessibility, gene expression, metabolic reprogramming, and SAT1 expression via ACSS2. **a**, Analysis of total acetylated H3K27 genomic loci in acetate-treated cells. The inner circle includes the differentially acetylated gene promoters upon acetate treatment. **b**, Heatmap shows the expression of 282 commonly upregulated genes in vehicle control- and acetate-treated S2-013 cells identified through ChIP-Seq and RNA-Seq. The scale bar represents log2 (fold change) values (n = 1 sample per group). **c**, PCA plot of HPAF-II cells treated with acetate under acidosis relative to vehicle control-treated cells as determined by LC–MS/MS-based metabolomics (n = 6 in both groups from independent biological replicates). **d**, Heatmap of top 25 regulated metabolites of HPAF-II cells treated with acetate under acidosis, presented as normalized row Z-score, as determined by LC–MS/MS-based metabolomics (n = 6 in each group from independent biological replicates). **e**, Relative mRNA levels of *ACSS2* gene in scrambled control (siScr) and *ACSS2* knockdown (si*ACSS2*) S2-013 cells in the presence and absence of acetate for 24 hrs under acidosis (n = 4 in each group from independent biological replicates). **f**, Relative mRNA levels of *ACSS2* and *SAT1* genes in scrambled siScr and si*ACSS2* HPAF-II cells upon acetate treatment for 24 hrs under acidosis (n = 4 in each group from independent biological replicates). **g**, Relative mRNA levels of *ACSS2* and *SAT1* genes in scrambled siScr and si*ACSS2* CFPAC-1 cells upon acetate treatment for 24 hrs under acidosis (n = 4 in each group from independent biological replicate). **h**, Relative mRNA levels of *ACSS2* and *SAT1* genes in scrambled siScr and si*ACSS2* T3M4 cells upon acetate treatment for 24 hrs under acidosis (n = 4 in each group from independent biological replicates). **i**, The relative levels of SAT1 and ACSS2 proteins in siScr and si*ACSS2* HPAF-II, CFPAC-1, and T3M4 cells upon acetate treatment for 24 hrs under acidosis. Immunoblots are representative of 2 independent experiments. One-way ANOVA with Bonferroni’s post-hoc test; mean ± s.e.m. (e,f,g,h). Source data

To explore the relationship between histone H3 acetylation and chromatin signatures at regulatory elements, we performed chromatin immunoprecipitation followed by sequencing (ChIP–seq) for H3K27 acetylation marks in acetate-treated S2-013 cells under low-pH conditions. We observed an increased abundance of H3K27ac near the transcription start site (TSS) and differential enrichment of acetylation signals upon acetate treatment in S2-013 cells (Fig. 4a,b). Analysis of the genes in the proximity of differentially enriched peaks revealed 10,773 differentially acetylated gene loci, which included 8,208 gene-specific promoter acetylations (Extended Data Fig. 7a). Pathway enrichment analysis of these genes identified ribosome biogenesis, oxidative phosphorylation and metabolic pathways to be significantly modulated (Fig. 4c). RNA sequencing (RNA–seq) analysis of acetate and vehicle-treated S2-013 cells under acidosis identified 1,948 differentially upregulated and 1,332 downregulated genes (fold change ≥1.5 or ≤0.75) upon acetate treatment (Fig. 4d). Furthermore, a comparative analysis of differentially upregulated genes in the RNA–seq data with genes having differential enrichment of H3K27 acetylation peaks in the promoter regions from ChIP–seq data identified a transcriptional signature consisting of 282 genes (Fig. 4e and Extended Data Fig. 7b). A total of 48 genes were regulated by acetate in the ACSS2-dependent manner in RNA–seq analysis (Fig. 4f). Next, we correlated these regulated genes with their clinical significance based upon the *P* value and regression coefficients from Cox proportional hazards regression analyses for each gene derived from the OncoLnc database for pancreatic cancer patients. Our analysis identified seven tumour suppressor genes (*ITGA7*, *SLC45A1*, *TPPP*, *SCN4A*, *GIPR*, *SOGA3* and *RAB6B*) and six oncogenes (*SAT1*, *SERPINE1*, *UPK2*, *SYT12*, *RASAL1* and *CDH3*) with significant false discovery rate (FDR)-adjusted *P* values (*q*-value) of less than 0.05 (Fig. 4g). Metabolomic analysis of acetate-treated S2-013 cells identified increased levels of polyamine biosynthetic pathway metabolites, including *N*^1^-acetylspermidine, which was upregulated by acetate treatment in both S2-013 and HPAF-II cells (Fig. 4h,i and Extended Data Fig. 7c,d). Based on our high-throughput screens, we prioritized the *N*^1^-spermidine/spermine acetyltransferase *SAT1* gene for further analysis. The promoter region of the *SAT1* gene showed enrichment for H3K27 acetylation under low-pH conditions upon acetate treatment (Fig. 4j). Correspondingly, SAT1 expression was upregulated by acetate at the transcript and protein levels in an ACSS2-dependent manner under low-pH conditions in multiple PDAC cell lines (Fig. 4k,l and Extended Data Fig. 7e–i). Thus, our integrated analysis identified that *SAT1* expression is regulated by acetate via ACSS2 under low-pH conditions.

### SAT1 mediates acetate-induced pancreatic cancer cell growth

**Fig. 5:**
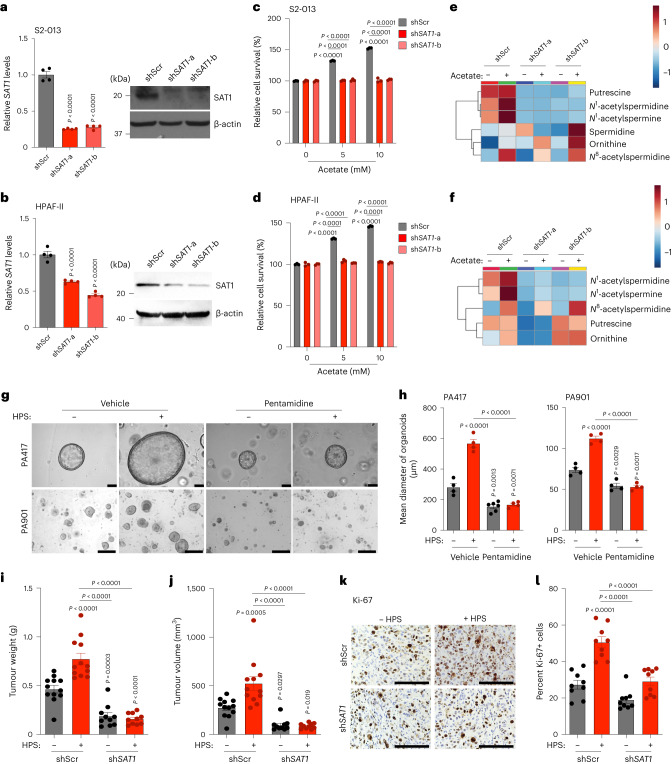
*SAT1* expression in PDAC cells is critical for acetate-mediated growth of pancreatic cancer cells in vitro and in vivo. **a**,**b**, Relative levels of *SAT1* mRNA and protein in control (shScr) and *SAT1* knockdown (sh*SAT1*-a and sh*SAT1*-b) S2-013 (**a**) and HPAF-II (**b**) cells under acidosis (*n* = 4 in each group, from independent biological replicates). Images are representative of two independent experiments. **c**,**d**, Relative survival of shScr and *SAT1* knockdown S2-013 (**c**) and HPAF-II (**d**) cells in the presence and absence of acetate under acidosis (*n* = 3 in each group, from independent biological replicates). **e**,**f**, Relative levels of polyamine biosynthetic pathway metabolites in shScr and sh*SAT1* S2-013 (**e**) and HPAF-II (**f**) cells in the presence and absence of 5 mM acetate under acidosis. Metabolites are presented as normalized row *Z*-scores from *n* = 6 (S2-013) and *n* = 4 (HPAF-II) biological replicates per group. **g**,**h**, Representative images (**g**) of PA417 and PA901 organoids cultured alone or in combination with HPS cells upon treatment with 25 µM pentamidine, along with mean organoid diameters (**h**). Scale bars, 100 µm. PA417, *n* = 4 (vehicle), *n* = 4 (+HPS), *n* = 6 (pentamidine), *n* = 4 (HPS + pentamidine), and PA901, *n* = 4 in each group from independent biological replicates. **i**,**j**, Tumour weights (**i**) and volumes (**j**) upon necropsy of mice implanted with control and *SAT1* knockdown S2-013 cells alone or co-implanted with HPS cells (*n* = 12 (sh*Scr*), *n* = 12 (sh*Scr* + HPS), *n* = 10 (sh*SAT1*), *n* = 10 (sh*SAT1* + HPS) mice). **k**,**l**, Representative IHC images for Ki-67 staining (**k**) in tumour sections from mice implanted with shScr or sh*SAT1* S2-013 cells alone or co-implanted with HPS cells along with the quantitation of percent positive cells (**l**). Scale bars, 100 µm. Ki-67 positive cells were counted in three different fields from three tumour sections of each group (*n* = 9 in each group). One-way ANOVA with Bonferroni’s post-hoc test, mean ± s.e.m. (**a**,**b**,**h**–**j**,**l**); two-way ANOVA with Tukey’s post-hoc test, mean ± s.e.m. (**c**,**d**). Source data

**Extended Data Fig. 8:**
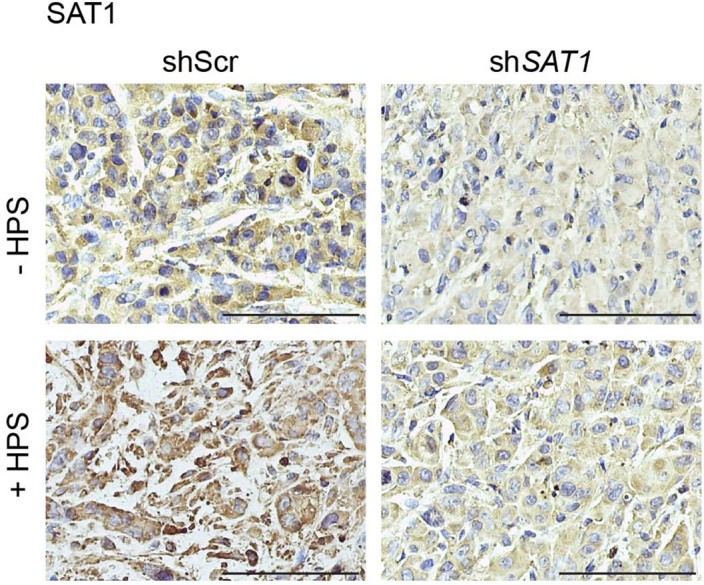
Validation of SAT1 knockdown in orthotopically implanted tumors. Representative IHC staining of SAT1 protein in the formalin-fixed tumor sections from athymic-nude mice implanted with control (shScr) and SAT1 knockdown (shSAT1) S2-013 cells alone or co-implanted with HPS cells. Scale bar = 100 µm. Representative image of images taken from three different fields from 3 tumor sections of each group.

Given the upregulation of SAT1 upon acetate treatment, we knocked down SAT1 in pancreatic cancer cells, which led to the abrogation of acetate-induced survival of cancer cells under acidosis (Fig. 5a–d). Acetate treatment significantly induced the levels of SAT1 products *N*^1^-acetylspermidine, *N*^1^-acetylspermine and *N*^8^-acetylspermidine, along with putrescine. However, *SAT1* knockdown decreased the levels of these metabolites, confirming the role of SAT1 in maintaining polyamine pools in the cell (Fig. 5e,f). Of note, inhibition of SAT1 by pentamidine, a SAT1 inhibitor^35^, significantly decreased the acetate-induced growth of PA417 and PA901 organoids in the organoid–HPS co-culture system (Fig. 5g,h). The knockdown of *SAT1* in S2-013 cells led to a significant reduction in the tumour burden in the orthotopic co-implantation model (Fig. 5i,j and Extended Data Fig. 8). *SAT1* knockdown in S2-013 cells also led to a reduction in cancer cell proliferation, based on Ki-67 staining in tumour sections (Fig. 5k,l). Hence, our data identify a critical role of SAT1 in mediating oncogenic crosstalk between tumour cells and stellate cells to regulate pancreatic tumour burden.

### Lysine 19 acetylation stabilizes SP1 and induces SAT1 levels

**Fig. 6:**
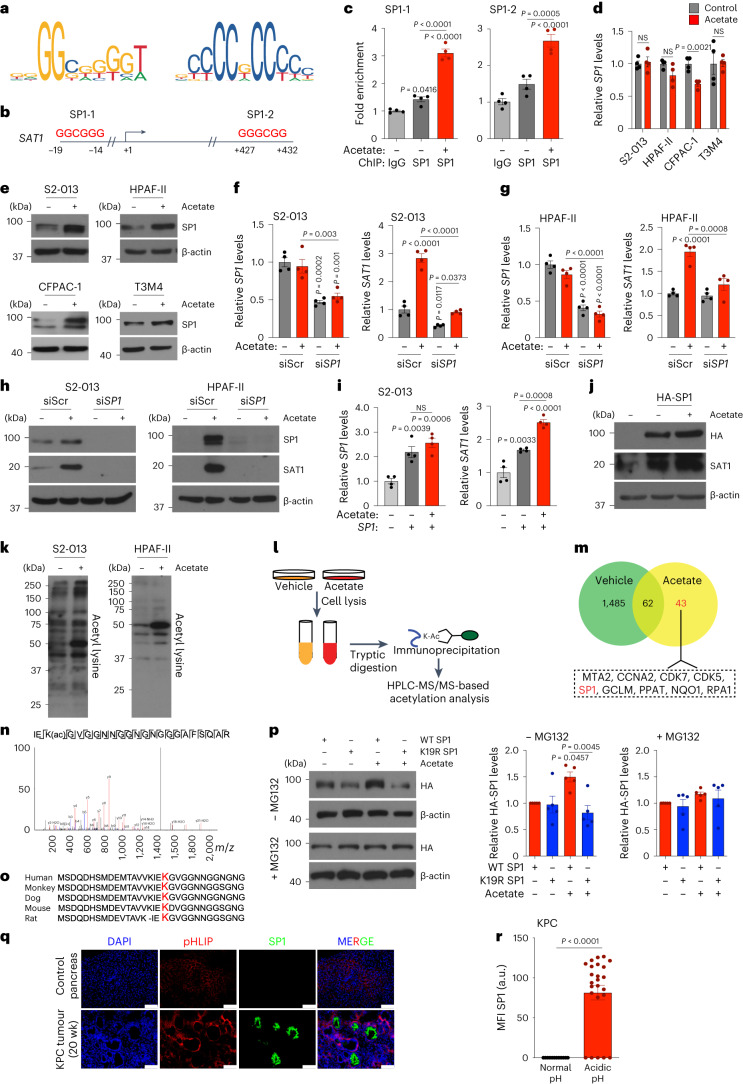
SP1-mediated *SAT1* regulation is critical for tumour cell survival under acidosis. **a**, Consensus sequence of the *SP1* binding motif. **b**, Putative binding sites of SP1 in the promoter region of the *SAT1* gene. **c**, Fold enrichment of SP1 binding in *SAT1* gene promoter in S2-013 cells upon acetate treatment normalized to IgG control (*n* = 4 from independent biological replicates). **d**,**e**, Relative levels of *SP1* mRNA (**d**) and protein (**e**) in PDAC cells upon acetate treatment (*n* = 4 from independent biological replicates; representative immunoblot images of two independent experiments). **f**,**g**, Relative *SP1* and *SAT1* mRNA levels in scrambled control (siScr) and *SP1* knockdown (si*SP1*) S2-013 (**f**) and HPAF-II (**g**) cells upon acetate treatment (*n* = 4 from independent biological replicates). **h**, Immunoblots of SP1 and SAT1 in siScr and si*SP1* S2-013 and HPAF-II cells, upon treatment with acetate. Images are representative of two independent experiments. **i**, Relative *SAT1* and *SP1* mRNA levels in *SP1-*overexpressing S2-013 cells upon acetate treatment (*n* = 4 from independent biological replicates). **j**, SAT1 and SP1 immunoblots in *SP1*-overexpressing S2-013 cells upon acetate treatment. Images are representative of two independent experiments. **k**, Immunoblot of lysine-acetylated proteins in S2-013 and HPAF-II cells treated with 0 and 5 mM acetate for 6 h. Images are representative of two independent experiments. **l**, Schematic representation of acetyl-lysine-modified proteome analysis of S2-013 cells treated with 5 mM acetate for 6 h. **m**, Venn diagram showing differentially acetylated proteins in S2-013 cells upon acetate treatment. **n**, LC–MS/MS analysis of acetylated peptide corresponding to SP1 (*n* = 2 from independent biological replicates). **o**, Peptide sequence alignment of SP1 (amino acids 1–31) in various species, highlighting the prospective acetylation site K19. **p**, Detection and quantitation (*n* = 5) of HA-tagged SP1 wild-type (WT) or K19R mutant in HEK293T cells treated with acetate, without or with MG132. Images are representative of five independent experiments. **q**,**r**, Representative immunofluorescent images (**q**) and quantitation (**r**) showing expression of SP1 in acidic tumour regions, as imaged by staining with pHLIP in tumour sections from 20-week-old KPC mice. Scale bars, 100 μm (*n* = 12 for normal pH, *n* = 24 for acidic pH). Unpaired, two-tailed *t*-test, mean ± s.e.m. (**d**,**r**); one-way ANOVA with Bonferroni’s post-hoc test, mean ± s.e.m. (**c**,**f**,**g**,**i**,**p**). Source data

**Extended Data Fig. 9:**
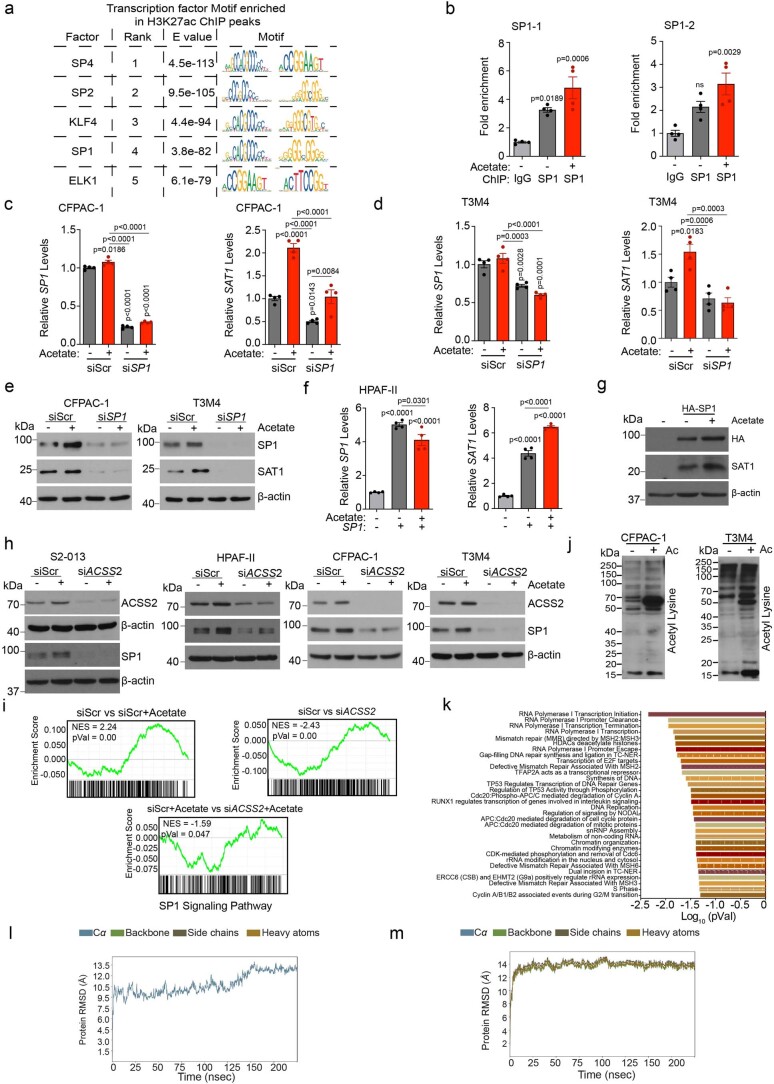
SP1 mediates acetate-induced regulation of SAT1 in an ACSS2-dependent manner. **a**, Transcription factor motif search for H3K27ac-bound peaks. **b**, Fold enrichment of SP1 binding on the promoter regions of *SAT1* gene in HPAF-II cells upon acetate treatment. The fold change values are normalized to IgG control (n = 4 in each group from independent biological replicates). **c**, Relative mRNA levels of *SP1* and *SAT1* genes in scrambled control (siScr) and *SP1* knockdown (si*SP1*) CFPAC-1 cells upon acetate treatment for 24 hrs (n = 4 in each group from independent biological replicates). **d**, Relative mRNA levels of *SP1* and *SAT1* genes in siScr and si*SP1* T3M4 cells upon acetate treatment (n = 4 in each group from independent biological replicates). **e**, Immunoblots showing the levels of SP1 and SAT1 proteins in siScr and si*SP1* CFPAC-1 and T3M4 cells, upon treatment with 5 mM acetate. Representative images of two independent experiments. **f, g** Relative mRNA (f) and protein (g) levels of SAT1 and SP1 in HPAF-II cells overexpressing *SP1* in the absence and presence of 5 mM acetate for 24 hrs under acidosis (f: n = 4 in each group from independent biological replicates; g: Representative images of two independent experiments). **h**, Immunoblots showing the levels of ACSS2 and SP1 proteins in siScr and si*ACSS2* PDAC cell lines upon treatment with 5 mM acetate for 24 hrs in acidosis. Representative image of two independent experiments. **i**, GSEA enrichment plot of SP1 signaling pathway in S2-013 cells between control cells (siScr) in presence and absence of acetate, siScr vs si*ACSS2* cells, and siScr vs siACSS2 both in the presence of acetate (statistics calculated using Permutation analysis through GSEA). **j**, Relative levels of lysine-acetylated proteins in CFPAC-1 and T3M4 cells upon treatment with 5 mM acetate for 6 hrs under acidosis. Representative images of two independent experiments. **k**, Pathway enrichment analysis of differentially regulated lysine-acetylated proteins in vehicle control- and acetate-treated S2-013 cells (statistics calculated using the Binomial test). **l,m**, Plot of RMSD for wild type SP1 (l) and SP1 acetylated at K19 position (m) for 250 nsec of simulations. One-way ANOVA with Bonferroni’s post-hoc test; mean ± s.e.m. (b,c,d,f). Source data

Histone modifications are tightly intertwined with gene expression through the regulation of binding specificities of transcription factors for promoters. Transcription factor binding analysis of the *SAT1* promoter using PROMO-ALGGEN identified SP1 binding sequences in the promoter of the *SAT1* gene (Fig. 6a,b)^36,37^. This finding is consistent with the identification of the SP1 consensus sequence as one of the most enriched transcription factors associated with the H3K27ac peaks spanning the gene promoters (Extended Data Fig. 9a). The ChIP analysis identified increased enrichment of SP1 at the indicated binding sites in the *SAT1* gene promoter, which was further increased upon acetate treatment (Fig. 6c and Extended Data Fig. 9b). Although *SP1* was not consistently upregulated at the mRNA level, it was robustly increased in multiple PDAC cell lines at the protein level upon acetate treatment under low-pH conditions (Fig. 6d,e). Knocking down *SP1* abrogated the acetate-induced increase in SAT1 at both transcript and protein levels under low-pH conditions (Fig. 6f–h and Extended Data Fig. 9c–e). Furthermore, overexpression of SP1 in pancreatic cancer cells resulted in increased expression of *SAT1*, which was further enhanced upon acetate treatment under low-pH conditions (Fig. 6i,j and Extended Data Fig. 9f,g). Remarkably, we observed that the acetate-mediated increase in SP1 protein levels under low-pH conditions was abrogated by *ACSS2* knockdown (Extended Data Fig. 9h). Furthermore, gene set enrichment analysis (GSEA) of RNA–seq data identified enrichment of SP1-regulated genes upon acetate treatment in an ACSS2-dependent manner (Extended Data Fig. 9i).

A previous study demonstrated that differential acetylation of SP1 protein regulates the expression of the target genes^38^. This led us to investigate the effect of acetate treatment on global acetylome changes in tumour cells under acidosis. An increase in levels of acetylated proteins was observed in multiple pancreatic cancer cell lines upon acetate treatment under low-pH conditions (Fig. 6k and Extended Data Fig. 9j). To further identify and quantify the global differences in acetylation patterns between vehicle and acetate-treated cells, we assessed proteome acetylation using acetyl-lysine affinity enrichment followed by quantitative mass spectrometry analysis (Fig. 6l). An unbiased quantification of the acetyl-lysine modified sites revealed 43 acetylated lysine sites from non-redundant proteins, exclusively acetylated in the acetate-treated cells (Fig. 6m). A Reactome analysis of the acetylated proteins revealed enrichment of biological pathways related to transcription, RNA processing, chromatin organization and DNA repair (Extended Data Fig. 9k). Interestingly, SP1 protein was differentially acetylated in acetate-treated cells (Fig. 6m). Furthermore, MS/MS spectra of SP1 protein cleavage identified the acetylation modification at the lysine 19 residue of the protein (Fig. 6n). The lysine 19 residue of the SP1 protein was conserved across multiple species, suggesting acetylation of this site may be an evolutionarily conserved mechanism regulating SP1 transcriptional activity (Fig. 6o). To investigate whether lysine 19 acetylation is needed for acetate-mediated stabilization of SP1, we performed site-directed mutagenesis to generate an SP1 mutant that could not be acetylated at lysine 19 (lysine 19 residue to arginine; K19R), and overexpressed it in cells in the presence or absence of the proteasome inhibitor MG132 under low-pH conditions. Although the treatment of cancer cells with acetate led to an induction of wild-type SP1 protein expression, K19R mutant SP1 failed to stabilize in the presence of acetate. However, MG132 treatment abolished any differences in the protein levels (Fig. 6p). Furthermore, molecular dynamics (MD) simulations for SP1 protein showed a gradual increase in the root-mean-square deviation (r.m.s.d.) value with fluctuations, stabilizing at an average of 13.5 Å, while the acetylated model showed a sudden increase in r.m.s.d. value with smaller fluctuations, stabilizing at an average of 14 Å. Thus, the acetylated model is highly stable compared to the unacetylated SP1 (Extended Data Fig. 9l,m). Taken together, these data identify an acetylation site in SP1 protein at the lysine 19 residue that is required for acetate-mediated stabilization.

### SP1 depletion diminishes CAF-induced tumour burden

**Fig. 7:**
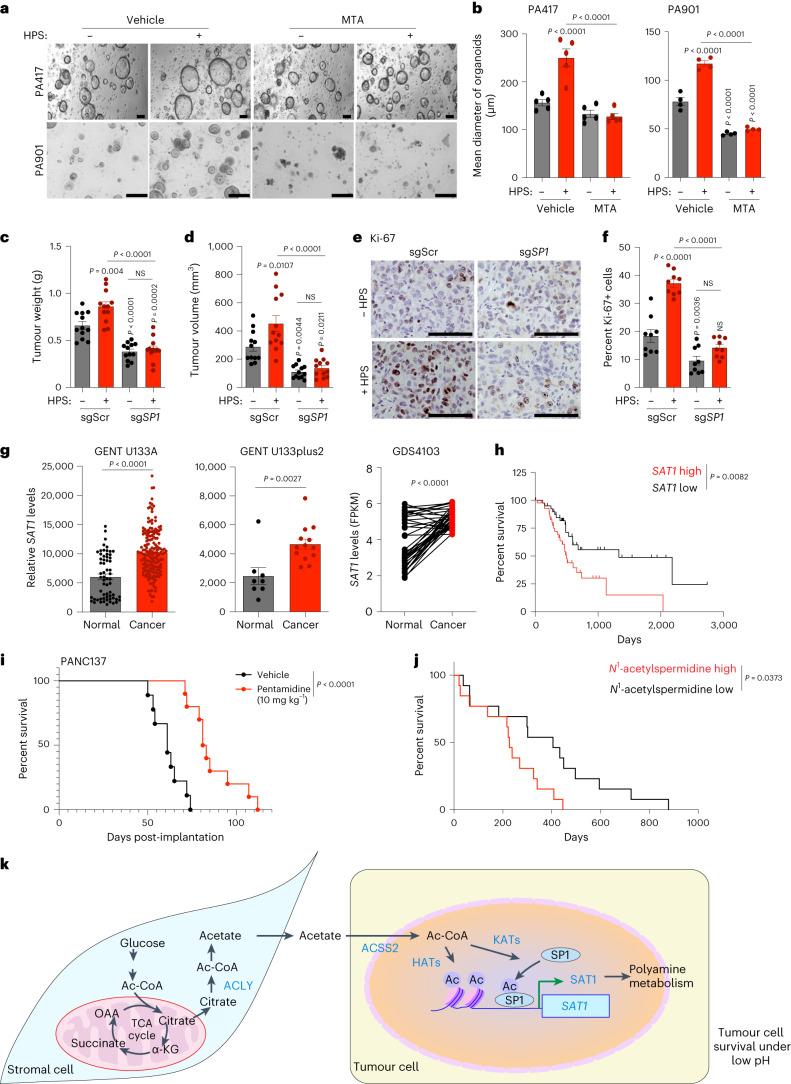
The ACSS2–SP1–SAT1 metabolic axis is critical for pancreatic cancer progression. **a**,**b**, Representative images (**a**) of PA417 and PA901 organoids cultured alone or in combination with HPS cells and treated with 100 and 25 µM mithramycin (MTA), respectively, and mean organoid diameters, represented as bar charts (**b**). Scale bars, 100 µm. PA417, *n* = 5 and PA901, *n* = 4 in each group from independent biological replicates. **c**,**d**, Tumour weights (**c**) and volumes (**d**) following necropsy of mice implanted with control (sgScr) or *SP1* knockout (sg*SP1*) S2-013 cells alone or co-implanted with HPS cells (*n* = 12 mice in each group). **e**,**f**, Representative IHC images for Ki-67 (**e**) in tumour sections from mice implanted with sgScr or sg*SP1* S2-013 cells alone or co-implanted with HPS cells, along with the quantitation of percent positive cells (**f**). Scale bars, 100 µm. Ki-67^+^ cells were counted in three different fields from three tumour sections of each group (*n* = 9 in each group). **g**, Meta-analysis of gene expression of *SAT1* in normal pancreas and pancreatic tumour samples (GENT U133A-normal (*n* = 62), cancer (*n* = 174); GENT U133plus2-normal (*n* = 8), cancer (*n* = 13); GDS4103-normal (*n* = 39), cancer (*n* = 39). **h**, Comparison of Kaplan–Meier survival curves for human patients with PDAC, with all high and low *SAT1* expression in the The Cancer Genome Atlas (TCGA) cohort stratified by highest quartile (*n* = 43) and lowest quartile (*n* = 43) of *SAT1* levels. **i**, Kaplan–Meier survival plot showing the relative survival of PANC137 PDX tumour-bearing mice upon treatment with vehicle (*n* = 9) or 10 mg kg^−1^ pentamidine (*n* = 10) (median survival: vehicle, 61 days; pentamidine, 82 days). **j**, Kaplan–Meier survival curves for stage IV human patients with PDAC, comparing patients (male, 16; female, 10; median age, 66 years; age range, 36–92 years) with above-median (high; *n* = 13) and below-median (low; *n* = 13) plasma levels of *N*^1^-acetylspermidine. **k**, Schematic illustration of the overall findings of the study. OAA, oxaloacetate; HAT, histone acetyl transferase; KAT, lysine acetyl transferase. Two-way ANOVA with Tukey’s post-hoc test, mean ± s.e.m. (**b**); one-way ANOVA with Bonferroni’s post-hoc test, mean ± s.e.m. (**c**,**d**,**f**); unpaired, two-tailed *t*-test, mean ± s.e.m. (**g**); paired two-tailed *t*-test, mean ± s.e.m. (**g**, GDS4103); Mantel–Cox log-rank test (**h**–**j**). Source data

**Extended Data Fig. 10:**
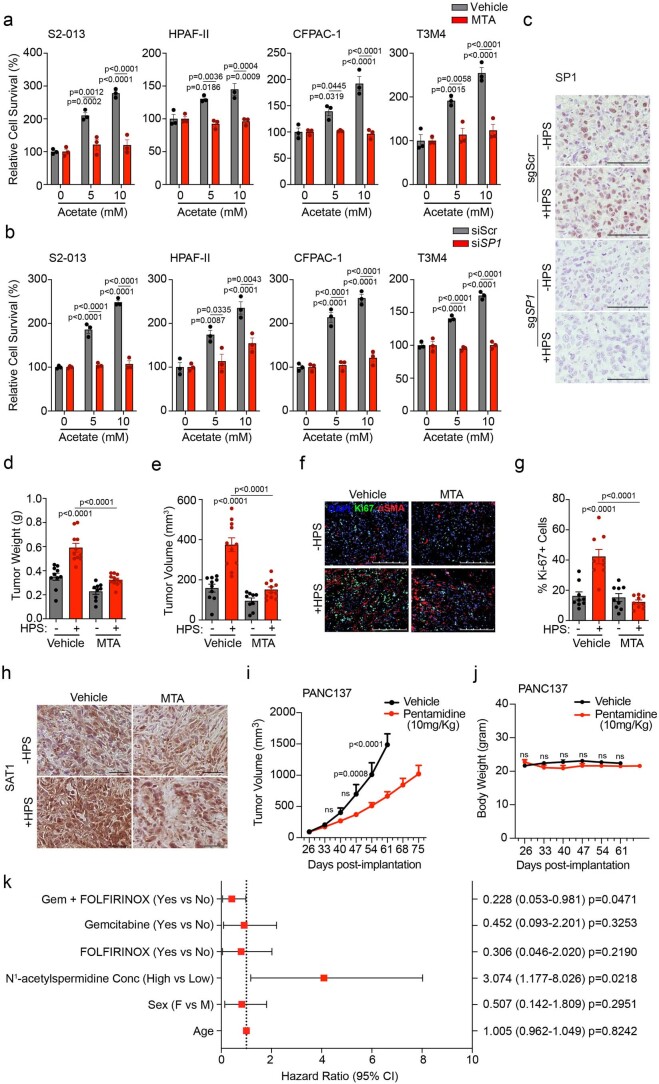
SP1/SAT1 inhibition or knockdown diminishes acetate-mediate survival advantage under acidosis and CAF-induced tumor burden in vivo. **a**, Relative survival of pancreatic cancer cells upon treatment with SP1 inhibitor mithramycin (MTA) in vehicle control- and acetate-treated cells for 72 hrs under acidosis. S2-013, HPAF-II, CFPAC-1, and T3M4 cells treated with 50 nM (S2-013, CFPAC-1, T3M4) and 100 nM (HPAF-II) mithramycin (n = 3 in each group from independent biological replicates). **b**, Relative survival of siScr and si*SP1* PDAC cell lines upon acetate treatment (n = 3 in each group from independent biological replicates). **c**, Representative IHC images for SP1 staining in tumor sections from athymic-nude mice implanted with sgScr or sgSP1 S2-013 cells alone or co-implanted with HPS cells. Scale bar = 100 µm. Images were captured from three different fields from 3 tumor sections of each group. **d,e**, Post necropsy tumor weights (d) and tumor volumes (e) from athymic-nude mice implanted with S2-013 cells alone (n = 10) or co-implanted with HPS cells upon treatment with vehicle control (n = 11) and MTA (tumor cells alone: n = 9, HPS co-implanted: n = 11). All groups were compared to S2-013 cells alone-implanted group treated with vehicle control or as indicated. **f,g**, Representative immunofluorescent images showing the expression of Ki-67 and αSMA proteins (f) in tumor sections from athymic-nude mice implanted with S2-013 cells, alone or co- implanted with HPS cells, upon treatment with vehicle control and MTA. Scale bar = 250 µm. Ki- 67 positive cells (g) were counted manually in three different fields from 3 tumor sections of each group (n = 9 in each group). **h**, Representative IHC staining of SAT1 in tumor sections from athymic-nude mice implanted with S2-013 cells alone or co-implanted with HPS cells upon MTA treatment from three tumor sections of each group. Scale bar = 100 µm. Representative image of images taken from three different fields from 3 tumor sections of each group. **i,j**, Kinetics of tumor growth (i) and body weight changes (j) in athymic-nude mice orthotopically implanted with PANC137 human PDX tissue upon treatment with vehicle control or 10 mg/kg pentamidine (n = 9 (vehicle), 10 (pentamidine-treated) mice in group). **k**, Forest plot indicating Cox-proportional hazard regression analysis for stage IV PDAC patients for above and below median plasma N1-acetylspermidine levels, age, gender, and chemotherapy. Hazard ratios and 95% confidence intervals are presented and indicated on the right side. Cox-proportional hazard regression analysis. Two-way ANOVA with Tukey’s post-hoc test; mean ± s.e.m. (a,b); one-way ANOVA with Bonferroni’s post-hoc test; mean ± s.e.m. (d,e,g); two-way ANOVA with Bonferroni’s post-hoc test; mean ± s.e.m. (i,j). Source data

Next we investigated whether SP1 expression in PDAC tumours is dependent on the in vivo pH gradient. We observed a significant increase in SP1 in acidic regions in tumour tissue compared to tumour regions at physiological pH (Fig. 6q,r). Treatment of pancreatic cancer cells with mithramycin, an inhibitor of SP1^39^, significantly diminished acetate-mediated induction of tumour-cell survival at low pH (Extended Data Fig. 10a). Furthermore, knockdown of SP1 in S2-013 and HPAF-II cells by siRNA abrogated the acetate-induced increased survival of cancer cells in low-pH conditions (Extended Data Fig. 10b). Interestingly, the stellate cell-induced increase in the size of PA417 and PA901 organoids was also abrogated upon treatment with mithramycin in organoid–stellate cell co-culture models (Fig. 7a,b). We also investigated whether depletion of SP1 could abrogate CAF-induced tumour burden in vivo in tumour cell–CAF orthotopic co-implantation models. SP1 knockout in S2-013 cells abrogated the HPS-induced increase in tumour burden, as reflected in tumour weight and volume measurements upon necropsy (Fig. 7c,d and Extended Data Fig. 10c). Consistent with the tumour burden data, a significant decrease in proliferative Ki-67-positive cancer cells was observed in tumour tissue sections from nude mice implanted with sgScr or sgSP1 S2-013 cells, alone or in combination with HPS cells (Fig. 7e,f). Similarly, inhibition of SP1 with mithramycin decreased the tumour burden, which could not be compensated by co-implantation with the CAFs (Extended Data Fig. 10d–g). Furthermore, tumours co-implanted with HPS cells showed increased SAT1 expression, which was abolished by mithramycin (Extended Data Fig. 10h). *SAT1* expression was significantly upregulated in pancreatic cancer patient tissue RNA–seq data analysed from multiple publicly available databases (Fig. 7g)^40,41^. Furthermore, stratification of survival data of patients with pancreatic cancer based on *SAT1* expression showed a significantly lower overall survival in patients with high *SAT1* expression (median survival, 498 days) as compared to patients with low *SAT1* expression (median survival, 1,332 days) (Fig. 7h). Treatment of an orthotopic patient-derived xenograft model with the SAT1 inhibitor pentamidine led to a significant decrease in the tumour burden and an increase in the survival of tumour-bearing mice (Fig. 7i and Extended Data Fig. 10i,j). Notably, patients with stage IV pancreatic cancer with high plasma *N*^1^-acetylspermidine levels (median survival, 227 days) showed overall poor survival compared to patients with low plasma *N*^1^-acetylspermidine levels (median survival, 405 days) (Fig. 7j). After accounting for the confounding effects of age, gender and chemotherapy, patients with high plasma *N*^1^-acetylspermidine levels had 3.07 times (95% confidence interval (CI) = 1.18–8.03) higher risk of death than patients with low plasma *N*^1^-acetylspermidine levels (Extended Data Fig. 10k). These results suggest that SAT1-mediated polyamine metabolism contributes to aggressiveness in PDAC, and targeting SAT1 via pharmacological inhibition of SP1 can diminish tumour cell growth and tumour burden in PDAC.

## Discussion

Pancreatic tumours exhibit strong desmoplasia, and the extent of this correlates with poor patient survival. Increasing evidence demonstrates that tumour cells often rely on the establishment of a fibrotic stroma to acquire certain nutrients, which leads to aberrant interactions between cancer cells and stromal compartments^42^. We thus sought to identify candidate metabolic nodes/pathways critical for tumour–stromal interactions during pancreatic tumour development.

In this Article we have evaluated metabolites derived from tumour cell-educated stellate cells that could alter the survival and proliferation of pancreatic cancer cells. Utilizing the NMR-based unbiased metabolomics approach, we show that metabolic support from CAFs, in the form of acetate, supports the proliferation and growth of cancer cells during cancer progression. The cancer cells in the tumour microenvironment are subjected to a variety of stresses, including low nutrients, hypoxia and acidosis^28,43^. Our present study shows that CAF-secreted acetate remarkably impacts the survival of cancer cells in an acidic microenvironment, highlighting its context-specific function, and facilitates tumour growth. PDAC tumours are predominantly acidic due to extensive activation/stabilization of hypoxia-inducible factors and the resultant glycolytic phenotype. Furthermore, the *ACLY* knockdown in PSCs significantly reduced the secretion of acetate in the microenvironment. The *ACLY*-knockdown cells may require acetate as an acetyl-CoA source, and therefore do not relinquish it as a waste product, causing reduced acetate secretion. Alternatively, and consistent with previous observations in brain cells, we speculate that the acetyl-CoA synthesized by ACLY condenses with aspartate to produce *N*-acetyl aspartate (NAA), which is further catalysed by aspartoacylase (ASPA) to liberate the acetate moiety for metabolic functions^44^. The observed decrease in secreted acetate upon loss of stromal ACLY may potentially be due to diminished acetyl-CoA being available for catalysis by NAA synthetase and ASPA enzymes.

Acetyl-CoA-mediated histone modifications have been known to play an important role in the transcriptional regulation of cancer cells^45^. Owing to the short half-life of acetylation modifications, acetate released by the deacetylation process is actively re-captured by ACSS2 and channelized into downstream metabolic and epigenetic functions to fuel growth and survival^24,46^^,^. Our studies provide evidence for a role of ACSS2 and acetate in regulating polyamine levels in cancer cells via transcriptional reprogramming.

Polyamines are polycations that exhibit high binding affinities towards nucleic acids and ensure optimal control over cellular processes such as DNA replication, transcription, translation and cell-cycle progression^47–49^. The polyamine metabolite *N*^1^-acetylspermidine acts as a substrate for histone modification reactions^50^. In our PDAC models, acetate-derived acetyl-CoA directs chromatin modifications by histone acetylation under acidosis. Although the function of SAT1 is predominantly associated with polyamine excretion, the product of SAT1, *N*^1^-acetylspermine/spermidine, can be re-routed into polyamine biosynthesis by the activity of polyamine oxidase enzyme^51^. The exported *N*^1^-acetylspermidine can also be acted upon by transglutaminase 2 to form the crosslinked extracellular matrix in the desmoplastic lesions, augmenting tumorigenesis. Although the exact mechanism for SAT1-mediated pancreatic cancer progression has not been addressed in this Article, we postulate that SAT1-mediated polyamine homeostasis is critical for cancer cell growth.

Apart from the acetylation of histone proteins, acetylation of non-histone proteins alters their cellular functions related to tumorigenesis, cell proliferation and immune response^52^. Acetylation of the transcription factor SP1 has been previously reported to alter its DNA-binding ability and downstream transcriptional regulation^38^. We have identified an acetylation modification at the evolutionarily conserved lysine 19 residue of the SP1 protein that augments its protein stability to regulate *SAT1* transcription. Our study unravels an acetate-mediated mechanism to epigenetically regulate polyamine homeostasis in cancer cells, and acetylation of SP1 protein, impacting its stability and DNA-binding properties.

In conclusion, our work unravels a previously unexplored metabolic crosstalk between pancreatic cancer and stromal cells that facilitates pancreatic cancer survival in the acidic tumour microenvironment. We identify a mechanism of ACLY-dependent acetate secretion by stellate cells that orchestrates metabolic and epigenetic reprogramming in pancreatic cancer cells in an ACSS2-dependent manner. Furthermore, ACSS2 serves a dual function during acidosis by facilitating histone acetylation in cancer cells and enhancing the stability of the transcription factor SP1, which synergize to commence metabolic reprogramming under acidosis through the regulation of SAT1. Depletion of SAT1 or pharmacological inhibition of the transcriptional regulator SP1 using mithramycin in orthotopic tumour mouse models diminishes stromal cell-induced metabolic reprogramming and tumour burden (Fig. 7k). Although stromal ablation therapy for PDAC remains controversial, our study identifies a therapeutic metabolic vulnerability that targets tumour growth in the nutrient-deprived microenvironment while maintaining the stromal reaction.

## Methods

The research in this manuscript complies with relevant ethical regulations.

### Cell culture

CFPAC-1, HPAF-II and MIAPaCa2 pancreatic cancer cells were obtained from the American Type Culture Collection. S2-013 is a cloned subline of a human pancreatic tumour cell line, SUIT-2 (derived from liver metastasis). S2-013 and T3M4 cell lines were provided by M. A. Hollingsworth (Eppley Institute, The University of Nebraska Medical Center). The pancreatic cancer cell lines S2-013, HPAF-II, CFPAC-1, T3M4, MIAPaCa2 and KPC1245, as well as the stellate cell lines CAF-0911, primary CAF-0906, CAF-1003 and CAF-1016, human pancreatic stellate cells (HPSs) and HEK293T cells were cultured in Dulbecco’s modified Eagle’s medium (DMEM) supplemented with 10% fetal bovine serum (FBS) and 1:100 antibiotic–antimycotic (Gibco). The low pH of the media was adjusted in the range ~6.9–7.1 by adding 1 g l^−1^ NaHCO_3,_ and the control pH was set by using 3.7 g l^−1^ NaHCO_3_. The cell lines were routinely tested for mycoplasma contamination and confirmed by short tandem repeat (STR) profiling at the University of Arizona Genetics Core (UAGC) every six months or sooner.

### Origin, isolation and establishment of CAF lines

The isolation, establishment and characterization of the CAF cell lines was performed in accordance with ref. ^53^. Briefly, fresh tissue was obtained from residual pancreatic adenocarcinoma specimens from patients undergoing primary surgical resection at The University of Nebraska Medical Center. All human samples were obtained in accordance with the policies and practices of the Institutional Review Board of The University of Nebraska Medical Center. Samples of tissues after surgery were placed in DMEM supplemented with 10% FBS and penicillin–streptomycin. The tissue was minced and placed in 15-ml tubes and centrifuged for 2 min at 1,200*g*. The medium was aspirated and washed twice with phosphate buffered saline (PBS) supplemented with penicillin–streptomycin. The tissue was digested using digestion solution (10 mg ml^−1^ collagenase IV, 0.25% trypsin no EDTA, 100 mM CaCl_2_ in 0.9% of NaCl) at 37 °C for 1 h. The cells were washed in PBS and placed in DMEM supplemented with medium containing 15% FBS/DMEM, l-glutamine (2 mmol l^−1^), penicillin/streptomycin and amphotericin. After ~5 days, cells were able to grow out from the tissue clumps. The medium was changed every three days. All cells were maintained at 37 °C in a humidified atmosphere of 5% CO_2_. When the cancer-derived fibroblasts grew to confluence, cells were trypsinized and passaged 1:3. Cell purity was determined by IHC for αSMA and vimentin, and the morphology (spindle-shaped cells with cytoplasmic extensions) was assessed. The antibodies used were anti-human αSMA (clone 1A4) and anti-vimentin (clone V9).

### Organoid culture and assays

The organoid lines were generated as described in ref. ^54^. Briefly, PDX tissues were minced and incubated in digestion medium (containing 1 mg ml^−1^ collagenase XI, 10 μg ml^−1^ DNAse I, 10.5 μmol l^−1^ Y-27632 in human complete medium) at 37 °C with mild agitation for up to 1 h. The dissociated cells were collected and plated with Matrigel and grown in human complete feeding medium: advanced DMEM/F12, HEPES 10 mmol l^−1^, Glutamax 1×, A83-01 500 nmol l^−1^, hEGF 50 ng ml^−1^, mNoggin 100 ng ml^−1^, hFGF10 100 ng ml^−1^, hGastrin I 0.01 μmol l^−1^, *N*-acetylcysteine 1.25 mmol l^−1^, nicotinamide 10 mmol l^−1^, PGE2 1 μmol l^−1^, B27 supplement 1× final, R-spondin1 conditioned medium 10% final and Wnt3A-conditioned medium 50% final. For fibroblast co-culture experiments, 2 × 10^3^ HPS cells per well were suspended in Matrigel and plated with 1 × 10^3^ organoid cells. After 72 h in culture, the number and size of the organoids were evaluated by microscopy.

### Conditioned medium preparation from stellate cells

Stellate cells were cultured in 10-cm dishes with DMEM and 10% FBS. At 80% confluence, cells were cultured in 10 ml of serum-free DMEM for 48 h, and the secreted factors were collected. The medium was collected and centrifuged at 135*g* for 3 min to remove cell debris. The conditioned medium, thus collected, was then utilized to treat pancreatic cancer cell lines or stored at −80 °C until use.

### Cell viability assays

Cell viability was determined by 3-[4,5-dimethylthiazol-2-yl]-2,5-diphenyltetrazolium bromide (MTT) assays, as described previously^17^. Briefly, 2,000 cells per well were seeded in a 96-well plate, 12 h before the indicated treatment. The medium was aspirated, then the cells were washed with 1× PBS and treated with the indicated chemical agents for 72 h in low-pH medium. At the end of the treatment, 50% vol/vol of a solution of MTT agent (1 mg ml^−1^) was added for 2 h. The medium containing MTT was aspirated, and the MTT crystals within the cells were dissolved in 100 µl DMSO. Relative cytotoxicity was determined by measuring the absorbance at 570 nm using a Cytation 3 plate reader (BioTek Instruments).

### NMR-based metabolomics

S2-013 PDAC cells and CAF 09-11 human PSCs were cultured in complete DMEM supplemented with 10% FBS and 25 mM U-^13^C_6_ glucose (Cambridge Isotope Laboratories). U-^13^C_6_ glucose-labelled CAF 09-11 cells were cultured with and without unlabelled tumour cell-conditioned medium. ^13^C_6_-labelled polar metabolites secreted into the medium were extracted from the medium using 80% methanol extraction. All NMR experiments were conducted at 298 K using a Bruker AVANCE III-HD 700-MHz spectrometer equipped with a 5-mm quadruple resonance QCI-P cryoprobe (^1^H, ^13^C, ^15^N and ^31^P) with *z*-axis gradients. A SampleJet automated sample changer system with Bruker ICON-NMR software was used to automate the NMR data collection. Two-dimensional (2D) ^1^H-^13^C heteronuclear single quantum coherence (HSQC) spectra were collected and analysed as described previously^55^.

### LC–MS/MS-based polar metabolomics

Relative quantification of the polar metabolites was performed using a selected reaction monitoring (SRM)-based mass spectrometry method^56^. The cells were washed with cold LC–MS-grade water, and the polar metabolites were extracted using 80% LC–MS-grade methanol. An ultra-performance liquid chromatography (UPLC)-based BEH amide column (150 mm × 2.1 mm, Waters) was used to separate the metabolites with a gradient buffer composition consisting of buffer A (100% acetonitrile) and buffer B (20 mM ammonium acetate, pH 9.0).

### Acetate estimation

The level of acetate was quantified in plasma, tumour-derived interstitial fluids or PSC-derived conditioned medium using an acetate colorimetric assay kit according to the manufacturer’s instructions. Briefly, samples or acetate standards were mixed with reaction mixtures, incubated at room temperature for 40 min, and measured at an optical density of 450 nm in the Cytation 3 reader.

### Determination of polyamines by UPLC

Polyamines were analysed by reverse-phase, ion-paired UPLC and were normalized to protein content. UPLC was performed with an ACQUITY UPLC system (Waters), using a Waters BEH amide column (1.7 µm, 2.1 mm × 150 mm). Mobile phase A was acetonitrile, and mobile phase B was water with 20 mM ammonium formate, pH 4.0. The flow rate was maintained at 0.3 ml min^−1^.

### Chemicals

Sodium acetate (Sigma-Aldrich, S2889), C646 (Sigma-Aldrich, SML0002), orlistat (Cayman, 10005426), ACSS2 inhibitor (Millipore, 533756), mithramycin A (Cayman, 11434), pentamidine (Cayman, 20679), ^14^C_2_-acetic acid, (Perkin Elmer, NEC553050UC), 2-deoxyglucose (Sigma-Aldrich, D8375), 2,4-dinitrophenol (Sigma-Aldrich, D198501), rotenone (Sigma-Aldrich, cat. no. R8875), DAPI (Invitrogen, P36935), Turbofect transfection reagent (Thermo Fisher, R0532), DharmaFECT 1 transfection reagent (Horizon Discovery, T-2001), Nile red (Thermo Fisher, N1142), protein G magnetic beads (Thermo Fisher, 10004D), dispase II (Sigma-Aldrich, D4693), delipidated serum (Biowest, S181L), TRIzol (Thermo Fisher, 15596026), MG132 (EMD Millipore, 474790), PowerUP SYBR Green Mastermix (Applied Biosystems, A25742), MTT (Sigma-Aldrich, M2128), HA-magnetic beads (Thermo Fisher, 88836).

### shRNA knockdown

Short hairpins (sh) targeting human *ACLY*, *ACSS2* and *SAT1* and scrambled control (shScr) were used in this study. A pLKO.1 lentiviral expression vector containing the puromycin resistance gene was used to co-express individual shRNAs. Two hairpin sequences of each gene were selected that reduced protein levels by >70%. Recombinant lentiviral particles were produced by transient transfection of plasmids into HEK293T cells. In brief, 2.5 µg of the shRNA plasmid, 1 µg of the psPAX2 plasmid and 2 µg of the pMD2.G plasmid were transfected using Turbofect in HEK293T cells plated in a 100-mm cell-culture dish. Viral supernatant was collected 48 h and 72 h post-transfection and filtered using 0.45-µm filters before target cell infection. Cells were infected twice in 48 h with viral supernatant containing 10 mg ml^−1^ polybrene and were selected with 10 μg ml^−1^ puromycin. Expression of *ACLY*, *ACSS2* and *SAT1* was determined by reverse transcription-quantitative polymerase chain reaction (RT-qPCR) and western blot.

### siRNA construction and transfection

ON-TARGETplus SMARTpool siRNAs were purchased from Dharmacon (Horizon Discovery). The details of ACSS2, SP1 and the control siRNAs are listed in Supplementary Table 1. For siRNA transfections into cells, pancreatic cancer cells were grown to 30–50% confluence and then transfected with ACSS2/SP1 siRNA or the control siRNA using DharmaFECT 1 transfection reagent according to the manufacturer’s instructions. The levels of gene knockdown were evaluated by RT–qPCR and western blotting.

### Retroviral transduction

Phoenix-ECO cells were transfected with plasmid DNA using TurboFECT (Invitrogen) in antibiotic-free medium. At 24 h post-transfection, the medium was replaced with fresh medium. After 24 h, the retroviral medium was collected and filtered through a 0.45-μm filter. The retroviral particles were either directly used for target cell infection or frozen on dry ice and stored at −80 °C. The Phoenix-ECO cells were cultured with fresh medium to collect viral particles. S2-013 and HPAF-II cells were infected on two consecutive days using a mixture of retroviral medium and growth medium (1:1 vol/vol), supplemented with polybrene at 10 μg ml^−1^. After the second infection, the medium was replaced with fresh growth medium supplemented with G418 (Thermo Fisher) at 200 μg ml^−1^. The transduced cells were maintained in antibiotic selection medium for four days.

### RNA isolation and real-time PCR analysis

Total RNA was isolated from cells using TRIzol reagent (Invitrogen) following the manufacturer’s instructions. Total RNA was quantified, and 2,000 ng of RNA was reverse-transcribed to complementary DNA (cDNA) using an ABI High Capacity cDNA RT kit (Invitrogen). The cDNA was analysed by RT–qPCR to evaluate the expression of targeted genes using the SYBR Green PCR master mix (Applied Biosystems) in an ABI QuantStudio5 Thermocycler using QuantStudio Design & Analysis v1 software. The expression of each gene was normalized to the human 18S rRNA or mouse *Actb* gene. The primer pairs used in the study are listed in Supplementary Table 2. The data were analysed using the ∆∆Ct method, as described in ref. ^57^.

### Protein extraction and immunoblotting

Total cellular protein was extracted using RIPA buffer (50 mM Tris-HCl pH 8.0 containing 1% NP-40, 150 mM NaCl, 5 mM EDTA and 1 mM phenylmethylsulfonyl fluoride) containing protease and phosphatase inhibitors. The total protein was quantified using Bradford reagent. Equal amounts of protein lysates were separated on sodium dodecyl sulfate polyacrylamide gel electrophoresis (SDS–PAGE) and transferred to a polyvinylidene difluoride membrane (Millipore). The membranes were blocked with 5% skimmed milk for 2 h at room temperature. The blots were incubated with different primary antibodies overnight at 4 °C, followed by the appropriate horseradish peroxidase-conjugated secondary antibody (1:5,000) for protein visualization with ECL reagents (Millipore). We utilized the following primary antibodies: anti-ACLY (Cell Signaling, 4332), anti-ACeCS1 (Cell Signaling, 3658), anti-SAT1 (Cell Signaling, 61586), anti-SP1 (Cell Signaling, 9389), anti-acetyl-lysine (Cell Signaling, 9441), anti-β-actin (DHSB, JLA20), anti-acetyl-histone H3 (Lys9) (Cell Signaling, 9649), anti-acetyl-histone H3 (Lys27) (Cell Signaling, 4353), anti-acetyl-histone H3 (Lys18) (Cell Signaling, 9675), anti-histone H3 (Abcam, ab1791), anti-ACLY (Abcam, ab40793), anti-H3K27 (Active Motif, 39133) and HA antibody (Covance, MMS-101R). Protein expression levels were normalized to the respective loading control, β-actin.

### Lipid estimation by Nile red staining

Cancer cells were seeded to 25% confluence in a 24-well plate in DMEM containing 10% FBS. After the cells adhered to the plate, the medium was replaced with DMEM containing a combination of normal FBS and delipidated FBS (3:1, 1:1 and 1:3) at a final concentration of 5% with or without sodium acetate. At 96 h post-treatment, the cells were washed with PBS and stained with a 1 µg ml^−1^ solution of Nile red prepared in acetone for 15 min. The cells were washed three times with PBS, and the intracellular lipids were extracted using isopropanol. The fluorescence intensity of the extracted lipids was estimated at 552/636 nm using the Cytation 3 plate reader (BioTek Instruments).

### Analysis of ^14^C acetate incorporation into histones

The incorporation of radiolabelled acetate into histones was performed as in ref. ^58^. Briefly, pancreatic cancer cells were grown in 12-well plates to 70–80% confluence. Cells were treated with 1 μCi ml^−1^ [^14^C] acetate for 6 h in the presence or absence of the inhibitors. The cells were washed twice with ice-cold PBS, and lysed in hypotonic buffer (10 mM Tris-Cl (pH 8.0), 1 mM KCl, 1.5 mM MgCl_2_, 1 mM dithiothreitol) containing protease inhibitors. The lysates were subjected to one freeze–thaw cycle at −20 °C and centrifuged at 4 °C, 10,000*g* for 10 min. The supernatants were discarded, and the pellets were resuspended in 400 μl 0.4 N H_2_SO_4_ and vortexed until the pellets were dissolved. The lysates were rotated at 4 °C overnight and then centrifuged at 4 °C, 16,000*g* for 10 min. The supernatants were collected and counted for radioactivity using a scintillation counter.

### Oxygen consumption rate analysis

The oxygen consumption rate (OCR) was analysed with the Seahorse XF Cell Mito Stress Test in a Seahorse XFe96 Analyzer (Agilent Technologies). A total of 2,000 cells per well were plated in a 96-well Seahorse plate in DMEM medium containing 10% FBS. At 24 h post-seeding, the cells were treated as indicated. One day before the Seahorse assay, the Agilent Seahorse XFe96 Sensor Cartridge was hydrated with 200 ml per well of XF calibrant solution overnight in a non-CO_2_ incubator at 37 °C. On the day of the experiment, 100 ml of bicarbonate-free Seahorse assay medium containing 1 mM pyruvate, 2 mM glutamine and 25 mM glucose was prepared. The pH of the medium was adjusted to 7.4 with 1 N NaOH. Cells were washed twice with 200 µl of the Seahorse medium and incubated with 180 µl of the Seahorse medium per well in a non-CO_2_ incubator at 37 °C for 1 h. Meanwhile, the Seahorse sensor cartridge ports were loaded with 20 µl of 1 M 2,4-dinitrophenol (port A, 100 µM final concentration), 22 µl of 1 M 2-deoxyglucose (port B, 100 mM final concentration) and 25 µl of 10 µM rotenone (port C, 1 µM final concentration). The experimental design was set up using WAVE software, and measurements were performed in the Seahorse XFe96 Analyzer. Post measurement, supernatant from the cells was removed, and the cells were washed with PBS. Subsequently, the cells were lysed using a lysis buffer, and the estimated protein contents were used for normalization of the Seahorse data.

### ChIP–seq

Chromatins from S2-013 cells without or with acetate treatment were crosslinked using 1% formalin, and the reaction was quenched using 0.125 M glycine. Cells were lysed, and the chromatin was fragmented using a BiorupterPico sonicator (30 s on and 30 s off, 45 cycles). For each sample (S2-013 untreated, S2-013 (untreated) input, S2-013 acetate-treated, S2-013 (acetate-treated) input), 8 ng of purified ChIP DNA (detected by Qubit, Invitrogen) was acquired using the ChIP specific antibody H3K27Ac (Active Motif, #39133) and the Zymo-Spin ChIP kit according to the manufacturer’s instructions and recommendations. The captured and purified DNA was prepared for high-throughput sequencing using the New England Biolabs NEBNext Ultra II DNA Library Prep Kit for Illumina. The resulting indexed libraries were sequenced by the UNMC Sequencing Core Facility using an Illumina NextSeq 500 Genome Analyzer. Initial raw sequence files were processed based on the following steps. Adaptor sequences and low-quality (Phred score < 20) ends were trimmed from sequences using Trim Galore software package (http://www.bioinformatics.babraham.ac.uk/projects/trim_galore/). The resulting fastq files were aligned to the human genome (GRCh38/hg38) using the sequence aligner Bowtie2 (version 2.2.3)^59^. The software package Picard routine MarkDuplicates (http://broadinstitute.github.io/picard/) was used to remove sequence duplications. A sequencing count of 49.7 million was acquired using SAMtools (http://samtools.sourceforge.net/) for each sample. For peak calling of ChIP-enriched regions, the MACS2 peak-caller software (version 2.1.1) of each ChIP to the corresponding input DNA sample was used to determine binding regions based on an FDR-adjusted *P* value (*q*-value) of <0.25 (ref. ^60^). BigWig files were generated using the deepTools bamCoverage routine (https://deeptools.readthedocs.io/en/develop/). The alignment of significant peaks to gene-specific regions was accomplished using the BEDTools routine intersect (https://bedtools.readthedocs.io/en/latest/). For motif analysis, gene promoters (*n* = 8,990) −1,500 to +500 bp relative to the TSSs that contained an H3K27 increased enrichment region when comparing control to 6 h of acetate treatment were analysed using the software Centrimo local option and the Jaspar non-redundant vertebrate database, which contains 579 transcription factor binding profiles^61,62^. The ChIP–seq data were submitted to the Gene Expression Omnibus (GEO accession no. GSE160365).

### ChIP–PCR

The ChIP assay was carried out using the Pierce agarose ChIP kit according to the manufacturer’s instructions. In brief, cells were left untreated or treated with acetate for 6 h in low-pH conditions. Fragmented chromatin lysate was subjected to immunoprecipitation with an anti-SP1 antibody (9389S), followed by qPCR with primer sets specific to various regions of the SAT1 promoter. The qPCR data were analysed using relative quantification normalized against the untreated cells immunoprecipitated with immunoglobulin G (IgG). The delta Ct (∆Ct) for the difference between Ct values for the antibody of interest (SP1) and the negative antibody (IgG) was calculated. The fold enrichment was determined using the formula 2^–(∆Ct)^.

### RNA–seq analysis

RNA–seq analysis was performed on S2-013 cells treated with acetate. To define the differentially expressed genes, we utilized a fold change of ≥1.5 for upregulation or ≤0.75 for downregulation. The log-ranked file generated for differential expression between different groups was processed using GSEA2 v2.2.3 with 1,000 permutations in the classic scoring scheme using the c2.cp.kegg.v6.2.symbols.gmt and c2.tft.v6.1.symbols.gmt geneset database from the Broad Institute.

### Public dataset analysis

A meta-analysis of gene expression of *SAT1* in normal pancreas and pancreatic tumour samples was performed using the GENT2 database^41^. Study 1 included a gene expression profile across cancer experiments on the GPL96 platform (HG-U133A). Study 2 included a gene expression profile across various cancers experiments on the GPL570 platform (HG- U133_Plus_2). Study 3 included a gene expression profile across PDAC tumours and matched normal pancreatic tissue from patients with pancreatic cancer (International Classification of Functioning, Disability and Health (ICF)) on the GPL570 platform (HG- U133_Plus_2)^63^.

### Acetylation proteomics screen

The global acetylome was analysed using the PTM Scan Acetyl Motif Kit, according to the manufacturer’s instructions. Briefly, cells were lysed in urea denaturing buffer, and proteins were digested by trypsin. Vehicle- and acetate-treated cells were lysed in urea lysis buffer, sonicated at 15-W output with three bursts of 15 s each, and centrifuged for 15 min at 20,000*g* to remove insoluble material. The resultant supernatant was reduced using dithiothreitol and carboxamidomethylated by iodoacetamide. The protein extracts were digested overnight with trypsin (Worthington Biochemical Products). Peptides were further digested using lysopeptidase C before separation from non-peptide material with Sep-Pak C18 cartridges (Waters). The acetylated peptides were immunoprecipitated using the acetylated-lysine antibody provided in the kit. Peptides were cleared using Pierce PepClean C18 Spin columns and eluted into a total volume of 100 μl in 0.15% trifluoroacetic acid (TFA). The eluted peptides were concentrated using a speed vac.

Extracted peptides were resuspended in 2% acetonitrile (ACN) and 0.1% formic acid (FA) and loaded onto a trap column (Acclaim PepMap 100 75 µm × 2 cm C18 LC column, Thermo Scientific) at a flow rate of 4 µl min^−1^, then separated with a Thermo RSLC Ultimate 3000 system (Thermo Scientific) on a Thermo Easy-Spray PepMap RSLC C18 75 µm × 50 cm C18 2-μm column (Thermo Scientific) with a step gradient of 4–25% solvent B (0.1% FA in 80% ACN) from 10 to 90 min and 25–45% solvent B for 90 to 110 min at 300 nl min^−1^ and 50 °C with a 135-min total run time. Eluted peptides were analysed by a Thermo Orbitrap Fusion Lumos Tribrid (Thermo Scientific) mass spectrometer in a data-dependent acquisition mode. A survey full-scan MS (from *m*/*z* 350–1,800) was acquired in the Orbitrap with a resolution of 120,000. The automatic gain control (AGC) target for MS1 was set as 4 × 10^5^ and the ion filling time 100 ms. The most intense ions with charge states 2–6 were isolated in 3-s cycles and fragmented using higher-energy collisional dissociation fragmentation with 40% normalized collision energy and detected at a mass resolution of 30,000 at 200 *m*/*z*. The AGC target for MS/MS was set as 5 × 10^4^ and the ion filling time with a 60-ms dynamic exclusion set for 30 s with a 10-ppm mass window. Protein identification was performed by searching MS/MS data against the SwissProt human protein database downloaded on 20 April 2019. The search was set up for full tryptic peptides with a maximum of two missed cleavage sites. Acetylation of protein N termini and lysines, and oxidized methionine were included as variable modifications, and carbamidomethylation of cysteine was set as a fixed modification. The precursor mass-tolerance threshold was set at 10 ppm and the maximum fragment mass error was 0.02 Da. The analysis was performed using Proteome Discoverer 2.3 software. The significance threshold of the ion score was calculated based on an FDR of ≤1%.

### pHLIP injection, detection and colocalization analysis

Tumours were established either in the KPC spontaneous tumour progression model or in female athymic nude mice by orthotopic injection of pancreatic cancer cells into the mouse pancreas. When the tumours reached 5–6 mm in diameter, 100 μl of Alexa Fluor 546-pHLIPs (80 μM) were injected by tail-vein injections^34^. The animals were euthanized at 24 h post-injection, and necropsy was performed. Tumour tissues were collected and embedded in OCT, and 5-μm sections were mounted on slides. The sections were fixed with 4% paraformaldehyde and stained for ACSS2 or SP1 and pHLIP followed by staining with Alexa-488 anti-rabbit polyclonal antibodies. Sections were counterstained with DAPI. Immunofluorescence images were captured using a Leica DMI6000B microscope at ×200 magnification. Colocalization analysis was performed on the immunostained tumour sections using ZEN software from Zeiss. For quantitation of the mean fluorescent intensity (MFI) of ACSS2 or SP1 protein in normal and acidic regions from tumours of KPC mice and athymic nude mice implanted with S2-013 cells, an MFI of pHLIP > 20 units was considered a low-pH region, and MFI < 20 was considered a normal pH region.

### Site-directed mutagenesis

An *SP1* expression vector (pLXSN-SP1) was expanded in DH5α competent cells and used as a template to create the *SP1* mutants using the QuikChange II site-directed mutagenesis kit (Agilent Technologies), according to the manufacturer’s instructions. Briefly, to generate the K19R mutation, mutagenic primers (Eurofin) were designed using the Quick-Change Primer Design Program (Agilent Technologies). The first step of the site-directed mutagenesis involved mutant-strand synthesis by PCR using the mutagenic primers, following which the amplified products were treated with *DpnI* at 37 °C for 1 h to digest the parental methylated and hemimethylated DNA template. The newly synthesized mutated DNA was transformed into XL1-blue super competent cells (Agilent Technologies) for selection of the mutated *SP1* clones.

### IHC and immunofluorescence

Tumour tissues were fixed in 10% neutral buffered formalin shortly after euthanasia. After 24–48 h, fixed tissues were transferred to 70% ethanol. The tissues were embedded in paraffin, and 5-μm sections were mounted on slides. For IHC analysis of Ki-67, ACSS2, ACLY, SAT1 and SP1, antigen retrieval was performed by boiling in citrate buffer (pH 6.0). Slides were incubated with primary antibodies overnight at 4 °C at 1:200 (Ki67), 1:50 (ACeCS1), 1:20 (ACLY), 1:200 (SAT1) and 1:200 (SP1), and cleaved caspase-3 (1:200). We utilized the following primary antibodies: anti-Ki-67 (Cell Signaling, 12202), anti-αSMA (Thermo Fisher Scientific, MA5-11547), SAT1 (Invitrogen, PA1-16992), anti-ACeCS1 (Cell Signaling, 3658), anti-SP1 (Cell Signaling, 9389), anti-SP1 (Cell Signaling, 9389), anti-cleaved caspase-3 (Cell Signaling, 9664S), F-actin (Invitrogen, A12379) and vimentin (Neomarkers, MS-129-P). The stained sections were imaged and captured at ×200 magnification using a Leica DMI6000B inverted microscope or scanned by Roche Ventana iScan HT (Tissue Science Facility, UNMC). Cells with Ki-67 positive nuclear staining were counted from three random fields from a section, and a minimum of three tumour sections per group were analysed. The human patient tumour tissue sections were reviewed and scored as reported previously^17^. Immunofluorescence and colocalization analysis of ACLY with αSMA in PDAC patient tumour microarray (TMA) were performed as described previously^55^. Briefly, the TMA was first stained with anti-ACLY and anti-αSMA antibodies, followed by staining with Cy3-conjugated anti-mouse monoclonal antibody along with Alexa-488 anti-rabbit polyclonal antibodies. The sections were counterstained with DAPI. Immunofluorescence images were captured using a Leica DMI6000B microscope at ×200 magnification.

### MD simulation analysis for protein stability

SP1 protein structure was predicted using I-TASSER and the model with the best metric from I-TASSER was selected^64^. Post-translational modification (PTM) at the K19 position was confirmed using the online predictor for protein acetylation sites prediction using prediction of acetylation on internal lysine (PAIL)^65^. Acetylation at the K19 position was incorporated via Vienna-PTM 2.0. The modelled SP1 structure before and after PTM was utilized as an input structure to run MD simulations. Both structures were compared to determine the protein stability before and after PTM incorporation. A 250-ns-long MD simulation was performed using the Desmond module on Schrödinger’s Maestro platform. Solvation of the protein was performed using the TIP3P water model using the system builder tool of Desmond, and an orthorhombic simulation box with a buffer distance of 10 Å between the box edge and atoms of the complex was generated. The system was neutralized by adding a suitable number of counter-ions, and the isosmotic condition was maintained by adding 0.15 M NaCl to the simulation box. The MD simulation was performed at 300 K at atmospheric pressure (1.013 bar). A total of 1,000 frames were recorded and saved to the trajectory during the 250-ns simulation. A simulation interaction diagram was utilized for analysing the trajectory obtained for the MD simulation.

### Mouse strains

Congenitally athymic nude mice (NCr-nu/nu) and mice NOD *scid* gamma (NSG^TM^; NOD.Cg-*Prkdc*^*scid*^ *Il2rg*^*tm1Wjl*^/SzJ, (6–8-weeks old) were bred in-house or purchased from Jackson Laboratories (Strain# 005557). C57BL/6J mice (6–8-weeks old) were bred in-house. Mice were housed under pathogen-free conditions in a 12-h dark/12-h night (6:00 to 18:00) cycle at 70 °F with a humidity of ~50%. All animal experiments were performed with the approval of The University of Nebraska Medical Center Institutional Animal Care and Use Committee (IACUC).

Tumour tissues from 20–22-week-old female mice from the *Kras*^*LSL.G12D*/+^; *p53*^*R172H*/+^; *Pdx1-Cre*^*tg*/+^ (KPC) mouse spontaneous progression model of pancreatic cancer on a C57BL/6 background and littermate controls were collected by euthanizing the mice at the respective ages.

### Tumour growth studies and chemotherapy

Female athymic nude mice (NCr-nu/nu) were anaesthetized by intraperitoneal injection of ketamine/xylazine at a dosage of 0.1 mg per 10 g body weight. The mice were orthotopically implanted with 0.1 × 10^6^ pancreatic cancer cells along with 0.1 × 10^6^ shScrambled or sh*ACLY* stellate cells. Mice were monitored for 21–30 days post-implantation, and tumour volumes were measured manually using Vernier calipers. The maximal tumour size permitted by The University of Nebraska Medical Center Institutional Animal Care and Use Committee (IACUC) was 10 mm. All mice were euthanized when the tumour size reached 10 mm in any cohort, and tumour weight and volumes were measured upon necropsy. The maximal tumour size was not exceeded. The tumour volumes were calculated by the formula *V* = 1/2(length × width^2^). For assessing the effect of mithramycin A, we orthotopically implanted 0.1 × 10^5^ cancer cells alone or co-implanted with 0.1 × 10^5^ HPS cells in the pancreas of athymic nude mice. Ten days post-implantation, the mice were treated with mithramycin A (1 mg kg^−1^, twice a week) or saline as vehicle control. Mice were monitored for 27 days and were euthanized when the tumour size reached 10 mm in any cohort.

### Patient-derived xenograft studies

Six- to eight-week-old male NOD *scid* gamma (NSG^TM^; NOD.Cg-*Prkdc*^*scid*^ *Il2rg*^*tm1Wjl*^/SzJ) were used to implant PA137 PDX tumours. Tumour samples were cut into 3–4-mm pieces and immediately placed in F-12 HAM (Sigma-Aldrich) medium supplemented with 50% fetal calf serum and 50 units ml^−1^ penicillin and 50 μg ml^−1^ streptomycin. Tumour pieces were embedded into Matrigel before orthotopic implantation into mice. After implantation of tumour tissue, xenografts were allowed to grow for four weeks. When the tumour size reached 150 mm^3^, mice were randomly divided into two groups (vehicle control and pentamidine (10 mg kg^−1^, every day, through intraperitoneal injection)) and assessed for survival. Tumour volume and body weight were recorded regularly during the treatment. The mice were euthanized when their tumours reached a size of 15 mm in diameter.

### Human studies

Human PDAC patient plasma specimens were obtained from the UNMC Tissue Bank Rapid Autopsy Program (RAP) from deceased patients. No compensation was provided for participation in these studies. All studies with human subjects were conducted in compliance with approved UNMC IRB protocols and an informed consent waiver was approved by the UNMC IRB committee for all subjects.

### Statistics and reproducibility

All the experimental assays were performed at least in triplicate, or as otherwise indicated in figure legends, and the continuous values are represented as averages. The exact number of replicates is indicated in the figure legends. All data analyses were conducted using GraphPad Prism software (version 5 and version 8). Error bars indicate the standard error of the mean (s.e.m.). Statistical significance was estimated by unpaired/paired two-tailed Student’s *t*-test or one-way ANOVA with Bonferroni’s/Tukey’s multiple comparisons test. The distribution of survival data is described using Kaplan–Meier curves and compared between groups using a log-rank test. The sample size for animal studies was calculated using a two-sided Mann–Whitney test. No data were excluded from the analyses. The experiments were not randomized except for mice experiments, where the block randomization method was utilized. Data collection and analysis were not performed blind to the conditions of the experiments. However, in the case of human PDAC tissue studies, the investigator performing the metabolomics analysis did not have access to the survival data, so the metabolomics data were collected in a blinded manner. Male and female mice were utilized as indicated for each experiment. ChIP and sequencing were performed without replicates. Mass-spectrometry-based proteomic analysis was performed in duplicate. The data distribution was assumed to be normal, but this was not formally tested.

### Reporting summary

Further information on research design is available in the Nature Portfolio Reporting Summary linked to this Article.

## Online content

Any methods, additional references, Nature Portfolio reporting summaries, source data, extended data, supplementary information, acknowledgements, peer review information; details of author contributions and competing interests; and statements of data and code availability are available at 10.1038/s41556-024-01372-4.

## Supplementary information

Reporting Summary

Supplementary Table 1 List of siRNAs used in the Study

Supplementary Table 2 List of primers used in the Study

## Source data

Source Data Fig. 1 Statistical source data

Source Data Fig. 1 Unprocessed western blots

Source Data Fig. 2 Statistical source data

Source Data Fig. 2 Unprocessed western blots

Source Data Fig. 3 Statistical source data

Source Data Fig. 3 Unprocessed western blots

Source Data Fig. 4 Statistical source data

Source Data Fig. 4 Unprocessed western blots

Source Data Fig. 5 Statistical source data

Source Data Fig. 5 Unprocessed western blots

Source Data Fig. 6 Statistical source data

Source Data Fig. 6 Unprocessed western blots

Source Data Fig. 7 Statistical source data

Source Data Extended Data Fig. 1 Statistical source data

Source Data Extended Data Fig. 2 Statistical source data

Source Data Extended Data Fig. 2 Unprocessed western blots

Source Data Extended Data Fig. 3 Statistical source data

Source Data Extended Data Fig. 4 Statistical source data

Source Data Extended Data Fig. 4 Unprocessed western blots

Source Data Extended Data Fig. 5 Statistical source data

Source Data Extended Data Fig. 5 Unprocessed western blots

Source Data Extended Data Fig. 6 Statistical source data

Source Data Extended Data Fig. 7 Statistical source data

Source Data Extended Data Fig. 7 Unprocessed western blots

Source Data Extended Data Fig. 9 Statistical source data

Source Data Extended Data Fig. 9 Unprocessed western blots

Source Data Extended Data Fig. 10 Statistical source data

## Data Availability

ChIP–seq data that support the findings of this study have been deposited in the Gene Expression Omnibus (GEO) under accession code GSE160365. The data were made publicly available on 31 October 2023. Mass spectrometry data have been deposited in ProteomeXchange with the primary accession code PXD046270. The human PDAC data were derived from the TCGA Research Network (http://cancergenome.nih.gov/). The dataset derived from this resource that supports the findings of this study is available at Human Protein Atlas (https://www.proteinatlas.org/ENSG00000130066-SAT1/pathology/pancreatic+cancer). The RNA–seq data have been deposited to SRA, NCBI under accession code PRJNA1030046. The mRNA levels of SAT1 in pancreatic cancer tissues and the paired adjacent normal tissues were derived from the GEO database (GDS4103; https://www.ncbi.nlm.nih.gov/geo/tools/profileGraph.cgi?ID=GDS4103:213988_s_at). All other data supporting the findings of this study are available from the corresponding author upon reasonable request. Source data are provided with this paper.

## References

[1] Bachem, M. G. et al. Pancreatic carcinoma cells induce fibrosis by stimulating proliferation and matrix synthesis of stellate cells. *Gastroenterology* **128**, 907–921 (2005). 15825074 10.1053/j.gastro.2004.12.036

[2] Rhim, A. D. et al. Stromal elements act to restrain, rather than support, pancreatic ductal adenocarcinoma. *Cancer Cell* **25**, 735–747 (2014). 24856585 10.1016/j.ccr.2014.04.021 PMC4096698

[3] Lee, J. J. et al. Stromal response to Hedgehog signaling restrains pancreatic cancer progression. *Proc. Natl Acad. Sci. USA* **111**, E3091–E3100 (2014). 25024225 10.1073/pnas.1411679111 PMC4121834

[4] Kim, E. J. et al. Pilot clinical trial of hedgehog pathway inhibitor GDC-0449 (vismodegib) in combination with gemcitabine in patients with metastatic pancreatic adenocarcinoma. *Clin. Cancer Res.* **20**, 5937–5945 (2014). 25278454 10.1158/1078-0432.CCR-14-1269 PMC4254161

[5] Sherman, M. H. et al. Stromal cues regulate the pancreatic cancer epigenome and metabolome. *Proc. Natl Acad. Sci. USA* **114**, 1129–1134 (2017). 28096419 10.1073/pnas.1620164114 PMC5293019

[6] Sousa, C. M. et al. Pancreatic stellate cells support tumour metabolism through autophagic alanine secretion. *Nature* **536**, 479–483 (2016). 27509858 10.1038/nature19084 PMC5228623

[7] Gunda, V. et al. MUC1-mediated metabolic alterations regulate response to radiotherapy in pancreatic cancer. *Clin. Cancer Res.* **23**, 5881–5891 (2017). 28720669 10.1158/1078-0432.CCR-17-1151 PMC5626603

[8] Hu, T. et al. Metabolic rewiring by loss of Sirt5 promotes Kras-induced pancreatic cancer progression. *Gastroenterology* **161**, 1584–1600 (2021). 34245764 10.1053/j.gastro.2021.06.045 PMC8546779

[9] Islam, M. M., Goertzen, A., Singh, P. K. & Saha, R. Exploring the metabolic landscape of pancreatic ductal adenocarcinoma cells using genome-scale metabolic modeling. *iScience* **25**, 104483 (2022). 35712079 10.1016/j.isci.2022.104483 PMC9194136

[10] King, R. J. et al. CD73 induces GM-CSF/MDSC-mediated suppression of T cells to accelerate pancreatic cancer pathogenesis. *Oncogene* **41**, 971–982 (2022). 35001076 10.1038/s41388-021-02132-6 PMC8840971

[11] Mehla, K. & Singh, P. K. Metabolic subtyping for novel personalized therapies against pancreatic cancer. *Clin. Cancer Res.* **26**, 6–8 (2020). 31628144 10.1158/1078-0432.CCR-19-2926 PMC6942627

[12] Mullen, N. J. & Singh, P. K. Nucleotide metabolism: a pan-cancer metabolic dependency. *Nat. Rev. Cancer* **23**, 275–294 (2023). 36973407 10.1038/s41568-023-00557-7 PMC10041518

[13] Mullen, N. J. et al. ENT1 blockade by CNX-774 overcomes resistance to DHODH inhibition in pancreatic cancer. *Cancer Lett.* **552**, 215981 (2023). 36341997 10.1016/j.canlet.2022.215981 PMC10305837

[14] Olou, A. A., King, R. J., Yu, F. & Singh, P. K. MUC1 oncoprotein mitigates ER stress via CDA-mediated reprogramming of pyrimidine metabolism. *Oncogene* **39**, 3381–3395 (2020). 32103170 10.1038/s41388-020-1225-4 PMC7165067

[15] Tadros, S. et al. De novo lipid synthesis facilitates gemcitabine resistance through endoplasmic reticulum stress in pancreatic cancer. *Cancer Res.* **77**, 5503–5517 (2017). 28811332 10.1158/0008-5472.CAN-16-3062 PMC5645242

[16] Vernucci, E. et al. Metabolic alterations in pancreatic cancer progression. *Cancers (Basel)* **12**, 2 (2019). 31861288 10.3390/cancers12010002 PMC7016676

[17] Shukla, S. K. et al. MUC1 and HIF-1alpha signaling crosstalk induces anabolic glucose metabolism to impart gemcitabine resistance to pancreatic cancer. *Cancer Cell* **32**, 71–87 (2017). 28697344 10.1016/j.ccell.2017.06.004 PMC5533091

[18] Chaika, N. V. et al. Differential expression of metabolic genes in tumor and stromal components of primary and metastatic loci in pancreatic adenocarcinoma. *PLoS ONE* **7**, e32996 (2012). 22412968 10.1371/journal.pone.0032996 PMC3296773

[19] Pietrocola, F., Galluzzi, L., Bravo-San Pedro, J. M., Madeo, F. & Kroemer, G. Acetyl coenzyme A: a central metabolite and second messenger. *Cell Metab.* **21**, 805–821 (2015). 26039447 10.1016/j.cmet.2015.05.014

[20] Sivanand, S. et al. Nuclear acetyl-CoA production by ACLY promotes homologous recombination. *Mol. Cell* **67**, 252–265 (2017). 28689661 10.1016/j.molcel.2017.06.008 PMC5580398

[21] Campbell, S. L. & Wellen, K. E. Metabolic signaling to the nucleus in cancer. *Mol. Cell* **71**, 398–408 (2018). 30075141 10.1016/j.molcel.2018.07.015

[22] Park, J. W. et al. A prospective evaluation of 18F-FDG and 11C-acetate PET/CT for detection of primary and metastatic hepatocellular carcinoma. *J. Nucl. Med.* **49**, 1912–1921 (2008). 18997056 10.2967/jnumed.108.055087

[23] Tsuchida, T., Takeuchi, H., Okazawa, H., Tsujikawa, T. & Fujibayashi, Y. Grading of brain glioma with 1-11C-acetate PET: comparison with 18F-FDG PET. *Nucl. Med. Biol.* **35**, 171–176 (2008). 18312826 10.1016/j.nucmedbio.2007.11.004

[24] Lee, J. V. et al. Akt-dependent metabolic reprogramming regulates tumor cell histone acetylation. *Cell Metab.* **20**, 306–319 (2014). 24998913 10.1016/j.cmet.2014.06.004 PMC4151270

[25] Gao, X. et al. Acetate functions as an epigenetic metabolite to promote lipid synthesis under hypoxia. *Nat. Commun.* **7**, 11960 (2016). 27357947 10.1038/ncomms11960 PMC4931325

[26] Abrego, J. et al. GOT1-mediated anaplerotic glutamine metabolism regulates chronic acidosis stress in pancreatic cancer cells. *Cancer Lett.* **400**, 37–46 (2017). 28455244 10.1016/j.canlet.2017.04.029 PMC5488721

[27] Kondo, A. et al. Extracellular acidic pH activates the sterol regulatory element-binding protein 2 to promote tumor progression. *Cell Rep.* **18**, 2228–2242 (2017). 28249167 10.1016/j.celrep.2017.02.006

[28] Corbet, C. & Feron, O. Tumour acidosis: from the passenger to the driver’s seat. *Nat. Rev. Cancer* **17**, 577–593 (2017). 28912578 10.1038/nrc.2017.77

[29] Hingorani, S. R. et al. Trp53R172H and KrasG12D cooperate to promote chromosomal instability and widely metastatic pancreatic ductal adenocarcinoma in mice. *Cancer Cell* **7**, 469–483 (2005). 15894267 10.1016/j.ccr.2005.04.023

[30] Zhao, S. et al. ATP-citrate lyase controls a glucose-to-acetate metabolic switch. *Cell Rep.* **17**, 1037–1052 (2016). 27760311 10.1016/j.celrep.2016.09.069 PMC5175409

[31] Moffett, J. R., Arun, P., Ariyannur, P. S. & Namboodiri, A. M. *N*-Acetylaspartate reductions in brain injury: impact on post-injury neuroenergetics, lipid synthesis and protein acetylation. *Front. Neuroenergetics* **5**, 11 (2013). 24421768 10.3389/fnene.2013.00011 PMC3872778

[32] Kridel, S. J., Axelrod, F., Rozenkrantz, N. & Smith, J. W. Orlistat is a novel inhibitor of fatty acid synthase with antitumor activity. *Cancer Res.* **64**, 2070–2075 (2004). 15026345 10.1158/0008-5472.can-03-3645

[33] Carrer, A. et al. Acetyl-CoA metabolism supports multistep pancreatic tumorigenesis. *Cancer Discov.* **9**, 416–435 (2019). 30626590 10.1158/2159-8290.CD-18-0567 PMC6643997

[34] Weerakkody, D. et al. Family of pH (low) insertion peptides for tumor targeting. *Proc. Natl Acad. Sci. USA* **110**, 5834–5839 (2013). 23530249 10.1073/pnas.1303708110 PMC3625278

[35] Libby, P. R. & Porter, C. W. Inhibition of enzymes of polyamine back-conversion by pentamidine and berenil. *Biochem. Pharmacol.* **44**, 830–832 (1992). 1510731 10.1016/0006-2952(92)90424-h

[36] Farre, D. et al. Identification of patterns in biological sequences at the ALGGEN server: PROMO and MALGEN. *Nucleic Acids Res.* **31**, 3651–3653 (2003). 12824386 10.1093/nar/gkg605 PMC169011

[37] Messeguer, X. et al. PROMO: detection of known transcription regulatory elements using species-tailored searches. *Bioinformatics* **18**, 333–334 (2002). 11847087 10.1093/bioinformatics/18.2.333

[38] Hung, J. J., Wang, Y. T. & Chang, W. C. Sp1 deacetylation induced by phorbol ester recruits p300 to activate 12(*S*)-lipoxygenase gene transcription. *Mol. Cell. Biol.* **26**, 1770–1785 (2006). 16478997 10.1128/MCB.26.5.1770-1785.2006 PMC1430254

[39] Jia, Z. et al. Combined treatment of pancreatic cancer with mithramycin A and tolfenamic acid promotes Sp1 degradation and synergistic antitumor activity. *Cancer Res.* **70**, 1111–1119 (2010). 20086170 10.1158/0008-5472.CAN-09-3282 PMC2840649

[40] Shin, G. et al. GENT: gene expression database of normal and tumor tissues. *Cancer Inf.* **10**, 149–157 (2011). 10.4137/CIN.S7226 PMC3118449 21695066

[41] Park, S. J., Yoon, B. H., Kim, S. K. & Kim, S. Y. GENT2: an updated gene expression database for normal and tumor tissues. *BMC Med. Genomics* **12**, 101 (2019). 31296229 10.1186/s12920-019-0514-7 PMC6624177

[42] Vennin, C. et al. Reshaping the tumor stroma for treatment of pancreatic cancer. *Gastroenterology* **154**, 820–838 (2018). 29287624 10.1053/j.gastro.2017.11.280

[43] Zhu, J. & Thompson, C. B. Metabolic regulation of cell growth and proliferation. *Nat. Rev. Mol. Cell Biol.* **20**, 436–450 (2019). 30976106 10.1038/s41580-019-0123-5 PMC6592760

[44] Madhavarao, C. N. et al. Defective *N*-acetylaspartate catabolism reduces brain acetate levels and myelin lipid synthesis in Canavan’s disease. *Proc. Natl Acad. Sci. USA* **102**, 5221–5226 (2005). 15784740 10.1073/pnas.0409184102 PMC555036

[45] Suzuki, A. et al. Aberrant transcriptional regulations in cancers: genome, transcriptome and epigenome analysis of lung adenocarcinoma cell lines. *Nucleic Acids Res.* **42**, 13557–13572 (2014). 25378332 10.1093/nar/gku885 PMC4267666

[46] Bulusu, V. et al. Acetate recapturing by nuclear acetyl-CoA synthetase 2 prevents loss of histone acetylation during oxygen and serum limitation. *Cell Rep.* **18**, 647–658 (2017). 28099844 10.1016/j.celrep.2016.12.055 PMC5276806

[47] Casero, R. A. Jr. & Marton, L. J. Targeting polyamine metabolism and function in cancer and other hyperproliferative diseases. *Nat. Rev. Drug Discov.* **6**, 373–390 (2007). 17464296 10.1038/nrd2243

[48] Frugier, M., Florentz, C., Hosseini, M. W., Lehn, J. M. & Giege, R. Synthetic polyamines stimulate in vitro transcription by T7 RNA polymerase. *Nucleic Acids Res.* **22**, 2784–2790 (1994). 8052534 10.1093/nar/22.14.2784 PMC308248

[49] Yamashita, T. et al. Role of polyamines at the G1/S boundary and G2/M phase of the cell cycle. *Int. J. Biochem. Cell Biol.* **45**, 1042–1050 (2013). 23500523 10.1016/j.biocel.2013.02.021

[50] Hobbs, C. A. & Gilmour, S. K. High levels of intracellular polyamines promote histone acetyltransferase activity resulting in chromatin hyperacetylation. *J. Cell. Biochem.* **77**, 345–360 (2000). 10760944

[51] Casero, R. A. Jr, Murray Stewart, T. & Pegg, A. E. Polyamine metabolism and cancer: treatments, challenges and opportunities. *Nat. Rev. Cancer* **18**, 681–695 (2018). 30181570 10.1038/s41568-018-0050-3 PMC6487480

[52] Glozak, M. A., Sengupta, N., Zhang, X. & Seto, E. Acetylation and deacetylation of non-histone proteins. *Gene* **363**, 15–23 (2005). 16289629 10.1016/j.gene.2005.09.010

[53] Hwang, R. F. et al. Cancer-associated stromal fibroblasts promote pancreatic tumor progression. *Cancer Res.* **68**, 918–926 (2008). 18245495 10.1158/0008-5472.CAN-07-5714 PMC2519173

[54] Boj, S. F. et al. Organoid models of human and mouse ductal pancreatic cancer. *Cell* **160**, 324–338 (2015). 25557080 10.1016/j.cell.2014.12.021 PMC4334572

[55] Chaika, N. V. et al. MUC1 mucin stabilizes and activates hypoxia-inducible factor 1 alpha to regulate metabolism in pancreatic cancer. *Proc. Natl Acad. Sci. USA* **109**, 13787–13792 (2012). 22869720 10.1073/pnas.1203339109 PMC3427054

[56] Gunda, V., Yu, F. & Singh, P. K. Validation of metabolic alterations in microscale cell culture lysates using hydrophilic interaction liquid chromatography (HILIC)-tandem mass spectrometry-based metabolomics. *PLoS One* **11**, e0154416 (2016). 27120458 10.1371/journal.pone.0154416 PMC4847783

[57] Attri, K. S., Mehla, K., Shukla, S. K. & Singh, P. K. Microscale gene expression analysis of tumor-associated macrophages. *Sci. Rep.* **8**, 2408 (2018). 29402936 10.1038/s41598-018-20820-4 PMC5799305

[58] Comerford, S. A. et al. Acetate dependence of tumors. *Cell* **159**, 1591–1602 (2014). 25525877 10.1016/j.cell.2014.11.020 PMC4272450

[59] Langmead, B. & Salzberg, S. L. Fast gapped-read alignment with Bowtie 2. *Nat. Methods* **9**, 357–359 (2012). 22388286 10.1038/nmeth.1923 PMC3322381

[60] Zhang, Y. et al. Model-based analysis of ChIP-seq (MACS). *Genome Biol.* **9**, R137 (2008). 18798982 10.1186/gb-2008-9-9-r137 PMC2592715

[61] Bailey, T. L. & Machanick, P. Inferring direct DNA binding from ChIP-seq. *Nucleic Acids Res.* **40**, e128 (2012). 22610855 10.1093/nar/gks433 PMC3458523

[62] Fornes, O. et al. JASPAR 2020: update of the open-access database of transcription factor binding profiles. *Nucleic Acids Res.* **48**, D87–D92 (2020). 31701148 10.1093/nar/gkz1001 PMC7145627

[63] Badea, L., Herlea, V., Dima, S. O., Dumitrascu, T. & Popescu, I. Combined gene expression analysis of whole-tissue and microdissected pancreatic ductal adenocarcinoma identifies genes specifically overexpressed in tumor epithelia. *Hepatogastroenterology* **55**, 2016–2027 (2008). 19260470

[64] Yang, J. et al. The I-TASSER Suite: protein structure and function prediction. *Nat. Methods* **12**, 7–8 (2015). 25549265 10.1038/nmeth.3213 PMC4428668

[65] Li, A., Xue, Y., Jin, C., Wang, M. & Yao, X. Prediction of N^ε^-acetylation on internal lysines implemented in Bayesian Discriminant Method. *Biochem. Biophys. Res. Commun.* **350**, 818–824 (2006). 17045240 10.1016/j.bbrc.2006.08.199 PMC2093955

