# Author Correction: Cancer-associated fibroblast-derived acetate promotes pancreatic cancer development by altering polyamine metabolism via the ACSS2–SP1–SAT1 axis

**DOI:** 10.1038/s41556-024-01417-8

**Published:** 2024-04-19

**Authors:** Divya Murthy, Kuldeep S. Attri, Surendra K. Shukla, Ravi Thakur, Nina V. Chaika, Chunbo He, Dezhen Wang, Kanupriya Jha, Aneesha Dasgupta, Ryan J. King, Scott E. Mulder, Joshua Souchek, Teklab Gebregiworgis, Vikant Rai, Rohit Patel, Tuo Hu, Sandeep Rana, Sai Sundeep Kollala, Camila Pacheco, Paul M. Grandgenett, Fang Yu, Vikas Kumar, Audrey J. Lazenby, Adrian R. Black, Susanna Ulhannan, Ajay Jain, Barish H. Edil, David L. Klinkebiel, Robert Powers, Amarnath Natarajan, Michael A. Hollingsworth, Kamiya Mehla, Quan Ly, Sarika Chaudhary, Rosa F. Hwang, Kathryn E. Wellen, Pankaj K. Singh

**Affiliations:** 1 Eppley Institute for Research in Cancer and Allied Diseases, University of Nebraska Medical Center, Omaha, NE USA; 2 Department of Oncology Science, University of Oklahoma Health Sciences Center, Oklahoma City, OK USA; 3 Department of Biotechnology, School of Engineering and Applied Sciences, Bennett University, Greater Noida, Uttar Pradesh India; 4 Department of Biochemistry and Molecular Biology, University of Nebraska Medical Center, Omaha, NE USA; 5 Department of Chemistry, University of Nebraska-Lincoln, Lincoln, NE USA; 6 Department of Biochemistry, Schulich School of Medicine & Dentistry, University of Western Ontario, London, Ontario, Canada; 7 Department of Biostatistics, University of Nebraska Medical Center, Omaha, NE USA; 8 Department of Cell Biology, Genetics and Anatomy, University of Nebraska Medical Center, Omaha, NE USA; 9 Department of Pathology and Microbiology, University of Nebraska Medical Center, Omaha, NE USA; 10 Department of Internal Medicine, University of Oklahoma Health Sciences Center, Oklahoma City, OK USA; 11 Department of Surgery, University of Oklahoma Health Sciences Center, Oklahoma City, OK USA; 12 Nebraska Center for Integrated Biomolecular Communication, University of Nebraska–Lincoln, Lincoln, NE USA; 13 Department of Surgical Oncology, University of Nebraska Medical Center, Omaha, NE USA; 14 Department of Surgical Oncology, Division of Surgery, The University of Texas MD Anderson Cancer Center, Houston, TX USA; 15 Department of Cancer Biology, University of Pennsylvania Perelman School of Medicine, Philadelphia, PA USA; 16 OU Health Stephenson Cancer Center, University of Oklahoma Health Sciences Center, Oklahoma City, OK USA

**Keywords:** Pancreatic cancer, Cancer microenvironment, Cancer metabolism

Correction to: *Nature Cell Biology* 10.1038/s41556-024-01372-4, published online 1 March 2024.

This paper was originally published under a standard Springer Nature license (© The Author(s), under exclusive licence to Springer Nature Limited). It is now available as an open-access paper under a Creative Commons Attribution 4.0 International license, © The Author(s). The error has been corrected in the HTML and PDF versions of the article.

