# Author Correction: Cancer-associated fibroblast-derived acetate promotes pancreatic cancer development by altering polyamine metabolism via the ACSS2–SP1–SAT1 axis

**DOI:** 10.1038/s41556-026-02055-y

**Published:** 2026-08-04

**Authors:** Divya Murthy, Kuldeep S. Attri, Surendra K. Shukla, Ravi Thakur, Nina V. Chaika, Chunbo He, Dezhen Wang, Kanupriya Jha, Aneesha Dasgupta, Ryan J. King, Scott E. Mulder, Joshua Souchek, Teklab Gebregiworgis, Vikant Rai, Rohit Patel, Tuo Hu, Sandeep Rana, Sai Sundeep Kollala, Camila Pacheco, Paul M. Grandgenett, Fang Yu, Vikas Kumar, Audrey J. Lazenby, Adrian R. Black, Susanna Ulahannan, Ajay Jain, Barish H. Edil, David L. Klinkebiel, Robert Powers, Amarnath Natarajan, Michael A. Hollingsworth, Kamiya Mehla, Quan Ly, Sarika Chaudhary, Rosa F. Hwang, Kathryn E. Wellen, Pankaj K. Singh

**Affiliations:** 1 Eppley Institute for Research in Cancer and Allied Diseases, University of Nebraska Medical Center, Omaha, NE USA; 2 Department of Oncology Science, University of Oklahoma Health Sciences Center, Oklahoma City, OK USA; 3 Department of Biotechnology, School of Engineering and Applied Sciences, Bennett University, Greater Noida, Uttar Pradesh India; 4 Department of Biochemistry and Molecular Biology, University of Nebraska Medical Center, Omaha, NE USA; 5 Department of Chemistry, University of Nebraska-Lincoln, Lincoln, NE USA; 6 Department of Biochemistry, Schulich School of Medicine & Dentistry, University of Western Ontario, London, Ontario, Canada; 7 Department of Biostatistics, University of Nebraska Medical Center, Omaha, NE USA; 8 Department of Cell Biology, Genetics and Anatomy, University of Nebraska Medical Center, Omaha, NE USA; 9 Department of Pathology and Microbiology, University of Nebraska Medical Center, Omaha, NE USA; 10 Department of Internal Medicine, University of Oklahoma Health Sciences Center, Oklahoma City, OK USA; 11 Department of Surgery, University of Oklahoma Health Sciences Center, Oklahoma City, OK USA; 12 Nebraska Center for Integrated Biomolecular Communication, University of Nebraska–Lincoln, Lincoln, NE USA; 13 Department of Surgical Oncology, University of Nebraska Medical Center, Omaha, NE USA; 14 Department of Surgical Oncology, Division of Surgery, The University of Texas MD Anderson Cancer Center, Houston, TX USA; 15 Department of Cancer Biology, University of Pennsylvania Perelman School of Medicine, Philadelphia, PA USA; 16 OU Health Stephenson Cancer Center, University of Oklahoma Health Sciences Center, Oklahoma City, OK USA

**Keywords:** Pancreatic cancer, Cancer microenvironment, Cancer metabolism

Correction to: *Nature Cell Biology* https://doi.org/10.1038/s41556-024-01372-4, published online 1 March 2024.

In the version of this article initially published, the surname of Susanna Ulahannan was misspelled (Ulhannan); in Extended Data Fig. 5b, a representative blot used to confirm knockdown efficiency for the CFPAC-1 was also used in Extended Data Fig. 9h; and in Extended Data Fig. 5j, both images in shACSS2-A+HPS and shACSS2-B (without HPS) were from the same tissue; the ACSS2 IHC panel (top panel) has been replaced. The name and figure have been updated in the HTML and PDF versions of the article. For comparison, the original Extended Data Fig. 5 is available as Supplementary Information accompanying this amendment.

## Supplementary information

Original Extended Data Fig. 5

