# Supplementary material for: Cancer-associated fibroblast-derived acetate promotes pancreatic cancer development by altering polyamine metabolism via the ACSS2–SP1–SAT1 axis
Source: Nat Cell Biol. 2024 Mar 1;26(4):613–27. doi: 10.1038/s41556-024-01372-4 (PMC11021164; doi:10.1038/s41556-024-01372-4)
Supplement: Supplementary file 1 — Reporting Summary [file 41556_2024_1372_MOESM1_ESM.pdf]

nature research

Corresponding author(s): Pankaj K. Singh

Last updated by author(s): Jan 31, 2024

Reporting Summary

Nature Research wishes to improve the reproducibility of the work that we publish. This form provides structure for consistency and transparency in reporting. For further information on Nature Research policies, see our Editorial Policies and the Editorial Policy Checklist.

Statistics

For all statistical analyses, confirm that the following items are present in the figure legend, table legend, main text, or Methods section.

n/a	Confirmed	
☐	☒	The exact sample size (*n*) for each experimental group/condition, given as a discrete number and unit of measurement
☐	☒	A statement on whether measurements were taken from distinct samples or whether the same sample was measured repeatedly
☐	☒	The statistical test(s) used AND whether they are one- or two-sided<br>*Only common tests should be described solely by name; describe more complex techniques in the Methods section.*
☐	☒	A description of all covariates tested
☐	☒	A description of any assumptions or corrections, such as tests of normality and adjustment for multiple comparisons
☐	☒	A full description of the statistical parameters including central tendency (e.g. means) or other basic estimates (e.g. regression coefficient) AND variation (e.g. standard deviation) or associated estimates of uncertainty (e.g. confidence intervals)
☐	☒	For null hypothesis testing, the test statistic (e.g. *F*, *t*, *r*) with confidence intervals, effect sizes, degrees of freedom and *P* value noted<br>*Give P values as exact values whenever suitable.*
☒	☐	For Bayesian analysis, information on the choice of priors and Markov chain Monte Carlo settings
☒	☐	For hierarchical and complex designs, identification of the appropriate level for tests and full reporting of outcomes
☒	☐	Estimates of effect sizes (e.g. Cohen's *d*, Pearson's *r*), indicating how they were calculated

Our web collection on statistics for biologists contains articles on many of the points above.

Software and code

Policy information about availability of computer code

Data collection	qRT-PCR data were obtained using ABI QuantStudioS thermocycler (ABI Biosystem).<br>lmmunofluorescence images were obtained using Zeiss LSM 710 or Leica CTR HS DMl6000B inverted microscope<br>For proteomics, eluted peptides were analyzed using Thermo Orbitrap Fusion Lumos Tribrid (Thermo Scientific'") mass spectrometer.<br>ChIP-seq libraries were sequenced using lllumina NextSeq 500 Genome Analyzer.<br>Bruker ICON-NMR software was used to automate the NMR data collection.<br>Seahorse data was collected in Seahorse XF Pro Analyzer (Agilent Technologies, Inc.)
Data analysis	qRT PCR data was analyzed using using QuantStudio Design software (version1.5.2) and further analyzed using Graphpad Prism.<br>Immunofluorescence images were analyzed using Zen Black software (Zeiss LSM 710) or ImageJ/Fiji (Version 2.9.0) (Leica).<br>Statistical analyses performed with Graphpad Prism (Version 5, Version 8).<br>Seahorse experimental design was set up using the WAVE software.<br>Growth assays and microplate based-assays were analyzed in Cytation3 using Gen 3.03 software.<br>For ChIP-Seq data, initial raw sequence files were processed based on the following steps. Adaptor sequences and low quality (Phred score< 20) ends were trimmed from sequences using Trim Galore software package (http://www.bioinformatics.babraham.ac.uk/projects/trim_galore/). Resulting fastq files were aligned to the human genome (GRCh38/hg38) using the sequence aligner Bowtie2 (version 2.2.3) (Langmead and Salzberg, 2012). The software package Picard routine MarkDuplicates (http://broadinstitute.github.io/picard/) was used to remove sequence duplications. Sequencing count of 49.7 million was acquired using samtools (http://samtools.sourceforge.net/) for each sample. For peak calling of ChIP-enriched regions, the MACS2 peak caller software (version 2.1.1) of each ChIP to corresponding input DNA sample was used to determine binding regions based on an FDR adjusted p-value (q-value) <0.25 (Zhang et al., 2008). Bigwig files were generated using the deeptools bamCoverage routine (https://deeptools.readthedocs.io/en/develop/). The alignment of significant peaks to gene-specific regions was accomplished using the bedtools routine intersect (https://bedtools.readthedocs.io/en/latest/). For motif analysis, gene promoters (n=8990) -1500 to +500 bp relative to transcription start sites that contained H3K27 increased enrichment region when

comparing control to 6 hrs of acetate treatment were analyzed using the software Centrimo local option and the Jaspar non-redundant vertebrate database that contains 579 transcription factor binding profiles (Bailey and Machanick, 2012; Fornes et al., 2020).
For RNA-seq data, the log ranked file generated for differentially expressed between different groups was processed through GSEA2 v2.2.3 with 1000 permutations in the classic scoring scheme using the c2.cp.kegg.v6.2.symbols.gmt and c2.tft.v6.l.symbols.gmt geneset database from the Broad Institute.
For proteomics, eluted peptides were analyzed by a Thermo Orbitrap Fusion Lumos Tribrid (Thermo Scientific'") mass spectrometer in a data-dependent acquisition mode. Protein identification was performed by searching MS/MS data against the Swiss-Prat human protein database downloaded on Apr 20, 2019. The analysis was performed using Proteome Discoverer 2.3 software.
Metabolomics data was processed using MassLynx software (version) and analyzed using Metaboanalyst and skyline software (version).
Molecular dynamics simulation was performed using the Desmond module on Schrödinger's Maestro platform.

For manuscripts utilizing custom algorithms or software that are central to the research but not yet described in published literature, software must be made available to editors and reviewers. We strongly encourage code deposition in a community repository (e.g. GitHub). See the Nature Research guidelines for submitting code & software for further information.

Data

Policy information about availability of data

All manuscripts must include a data availability statement. This statement should provide the following information, where applicable:

- Accession codes, unique identifiers, or web links for publicly available datasets
- A list of figures that have associated raw data
- A description of any restrictions on data availability

ChIP–seq data that support the findings of this study have been deposited in the Gene Expression Omnibus (GEO) under accession code GSE160365 (https://www.ncbi.nlm.nih.gov/geo/query/acc.cgi?acc=GSE160365).
Mass spectrometry data have been deposited in ProteomeXchange with the primary accession code PXD046270 [https://proteomecentral.proteomexchange.org/cgi/GetDataset?ID=PXD046270].
The RNA sequencing data was submitted to SRA under the Bioproject ID PRJNA1030046 (https://www.ncbi.nlm.nih.gov/sra/?term=PRJNA1030046).
The human PDAC data were derived from the TCGA Research Network: http://cancergenome.nih.gov/. The data-set derived from this resource that supports the findings of this study is available in Human Protein Atlas (https://www.proteinatlas.org/ENSG00000130066-SAT1/pathology/pancreatic+cancer).
The mRNA levels of SAT1 in pancreatic cancer tissues and the paired adjacent normal tissues were derived from GEO database (GDS4103).

Field-specific reporting

Please select the one below that is the best fit for your research. If you are not sure, read the appropriate sections before making your selection.

☒ Life sciences ☐ Behavioural & social sciences ☐ Ecological, evolutionary & environmental sciences

For a reference copy of the document with all sections, see nature.com/documents/nr-reporting-summary-flat.pdf

Life sciences study design

All studies must disclose on these points even when the disclosure is negative.

Sample size	Based on the preliminary data, a sample of 9 animals per group for mice experiments (Fig 1) and 12 animals per group for Figure 7 was predicted to provide 80% power at α = 0.05 using a two-sided Mann-Whitney test assuming that the actual distribution was normal. In some experiments, some of the mice did not recover from the surgery and hence, we analyzed data from all the remaining mice.
Data exclusions	No data points were excluded from the study.
Replication	In vitro experiments were repeated at least three times, with at least three biological replicates for each experimental point, unless otherwise noted in the legend. Conclusions were drawn only if the results were consistently statistically robust. Precise values of n are stated in the figure legends.
Randomization	Post implantation, mice were randomized to receive mithramycin A treatments. We utilized block randomization method, where the entire cage was randomized for selection for treatment with mithramycin A or vehicle control. There was no allocation into different experimental groups for other experiments and hence, no randomization was performed other than mice experiments in this study.
Blinding	The researcher involved in mice treatments was the only one available and also happened be to the lead author on the study. Hence, blinding was not possible. For human PDAC tissue studies, the researcher running the metabolomics analysis did not have access to the survival data and hence, the metabolomics data was collected in a blinded manner.

Reporting for specific materials, systems and methods

We require information from authors about some types of materials, experimental systems and methods used in many studies. Here, indicate whether each material, system or method listed is relevant to your study. If you are not sure if a list item applies to your research, read the appropriate section before selecting a response.

Materials & experimental systems

n/a	Involved in the study
☐	☒ Antibodies
☐	☒ Eukaryotic cell lines
☒	☐ Palaeontology and archaeology
☐	☒ Animals and other organisms
☐	☒ Human research participants
☒	☐ Clinical data
☒	☐ Dual use research of concern

Methods

n/a	Involved in the study
☐	☒ ChIP-seq
☒	☐ Flow cytometry
☒	☐ MRI-based neuroimaging

Antibodies

Antibodies used

We utilized the following primary antibodies: Anti-ACLY (1:1000, Cell Signaling, 4332), Anti-ACeCS1 (1:1000, Cell Signaling, 3658), Anti-SAT1 (1:500, Thermo Fisher Scientific, PA1-16992), Anti-SP1 (1:1000, Cell Signaling, 9389), Anti-Acetyl Lysine (1:1000, Cell Signaling, 9441), Anti-β-actin (1:5000, DSHB, JLA20), Anti-Acetyl-Histone H3 (Lys9) (1:1000, Cell signaling, 9649), Anti-Acetyl-Histone H3 (Lys27) (1:1000, Cell signaling, 4353), Anti-Acetyl-Histone H3 (Lys18) (1:1000, Cell signaling, 9675), Anti-Histone H3 (1:5000, Abcam, ab1791), Anti-ACLY (1:200, Abcam, ab40793, IF), Anti-H3K27 (Active Motif, 39133), HA antibody (1:1000, Covance, MMS-101R), Vimentin (1:100, Santa Cruz, sc-6260), anti-Ki-67 (1:200, Cel Signaling, 12202), anti-cleaved caspase 3 (1:400, Cell Signaling, 9661), HRP-conjugated anti-rabbit (1:5000, Jackson ImmunoResearch, 111-035-003), HRP conjugated anti-mouse antibody (1:5000, Jackson ImmunoResearch, 115-035-003).

Validation

Antibodies used in the manuscript are well-published commercially available antibodies. Validation statements can be found on manufacturer's website.
Anti-ACLY (https://www.cellsignal.com/products/primary-antibodies/atp-citrate-lyase-antibody/4332),
Anti-ACeCS1 (https://www.cellsignal.com/products/primary-antibodies/acecs1-d19c6-rabbit-mab/3658),
Anti-SAT1 (https://www.thermofisher.com/antibody/product/SAT1-Antibody-Polyclonal/PA1-16992),
Anti-SP1 (https://www.cellsignal.com/products/primary-antibodies/sp1-d4c3-rabbit-mab/9389),
Anti-Acetyl Lysine (https://www.cellsignal.com/products/primary-antibodies/acetylated-lysine-antibody/9441),
Anti-β-actin (https://dshb.biology.uiowa.edu/JLA20),
Anti-Acetyl-Histone H3 (Lys9) (https://cellsignal.com/products/primary-antibodies/acetyl-histone-h3-lys9-c5b11-rabbit-mab/9649,
Anti-Acetyl-Histone H3 (Lys27) (https://www.cellsignal.com/products/primary-antibodies/acetyl-histone-h3-lys27-antibody/4353),
Anti-Acetyl-Histone H3 (Lys18) (https://www.cellsignal.com/products/primary-antibodies/acetyl-histone-h3-lys18-antibody/9675),
Anti-Histone H3 (https://www.abcam.com/products/primary-antibodies/histone-h3-antibody-nuclear-marker-and-chip-grade-ab1791.html),
Anti-ACLY (https://www.abcam.com/products/primary-antibodies/atp-citrate-lyase-antibody-ep704y-ab40793.html#lb),
Anti-H3K27 (https://www.activemotif.com/catalog/details/39133/histone-h3-acetyl-lys27-antibody-pab),
HA antibody (https://www.biolegend.com/nl-be/products/anti-ha-11-epitope-tag-antibody-11071),
Vimentin (https://www.scbt.com/p/vimentin-antibody-v9?gclid=CjwKCAjwp8OpBhAFEiwAG7NaEoJPArQfmOwAFCwuvrWNCcscO1GwpV7B7nPz-LmjaWU3Z1A_pYomSxoCr9kQAvD_BwE).

Eukaryotic cell lines

Policy information about cell lines

Cell line source(s)

CFPAC-1, HPAF-II, HEK293T, and MIAPaCa2 pancreatic cancer cells were obtained from the American Type Culture Collection (Rockville, MD). S2-013 is a cloned sub-line of a human pancreatic tumor cell line SUIT-2 (SUIT2 was derived from 73 year old male patient with pancreatic adenocarcinoma, liver metastasis). S2-013, T3M4 and Phoenix-ECO cell lines were provided by Dr. Michael A. Hollingsworth, Eppley Institute, UNMC, Omaha, NE).

Authentication

The cell lines were confirmed by STR profiling at the University of Arizona Genetics Core (UAGC).

Mycoplasma contamination

The cell lines were routinely tested for mycoplasma contamination by a PCR-based assay and were found to be negative.

Commonly misidentified lines
(See ICLAC register)

We did not use any misidentified lines.

Animals and other organisms

Policy information about studies involving animals; ARRIVE guidelines recommended for reporting animal research

Laboratory animals

These studies utilized male and female congenitally athymic nude mice (NCr-nu/nu mice, 6-8 weeks age), NOD scid gamma mice (NOD.Cg-Prkdcscid Il2rgtm1Wjl/SzJ, 6-8 weeks age), or 20-22 weeks-old C57-BL/6 mice from KrasLSL.G12D/+; p53R172H/+; Pdx1-Cretg/+ (KPC) on C57BL/6 background.

Wild animals

No wild animals were used.

Field-collected samples

No field collected samples were used.

Ethics oversight	All animal experiments were performed with the approval of the University of Nebraska Medical Center Institutional Animal Care and Use Committee (IACUC).

Note that full information on the approval of the study protocol must also be provided in the manuscript.

Human research participants

Policy information about studies involving human research participants

Population characteristics	For quantifying N1-acetylspermidinde levels, we utilized 26 stage IV human PDAC patients comparing patients (male: 16, female: 10; median age: 66; age-range: 36-92) treated with FOLFIRINOX or Gemcitabine therapies or both.
Recruitment	The tissues were harvested from deceased PDAC patients via a RAPID autopsy program. No patients were recruited for these studies.
Ethics oversight	The tissues harvested from deceased PDAC patients were considered IRB exempt by the UNMC IRB.

Note that full information on the approval of the study protocol must also be provided in the manuscript.

ChIP-seq

Data deposition

☒ Confirm that both raw and final processed data have been deposited in a public database such as GEO.

☒ Confirm that you have deposited or provided access to graph files (e.g. BED files) for the called peaks.

Data access links<br>*May remain private before publication.*	(GEO: GSE160365) https://www.ncbi.nlm.nih.gov/geo/query/acc.cgi?acc=GSE160365
Files in database submission	GSM4872297- S2-013 cells, control treatment, ChIP-seq<br>GSM4872298- S2-013 cells, control treatment, input<br>GSM4872299- S2-013 cells, 6 hour acetate treatment, ChIP-seq<br>GSM4872300- S2-013 cells, 6 hour acetate treatment, input
Genome browser session<br>(e.g. UCSC)	(GEO: GSE160365) https://www.ncbi.nlm.nih.gov/geo/query/acc.cgi?acc=GSE160365

Methodology

Replicates	S2-013 cell were cultured in low pH conditions and treated with vehicle or acetate. ChIP-seq was performed for anti-H2K27ac antibody (Active motif; Cat#39133). Single ChIP replicate was performed for each condition.
Sequencing depth	Sequencing count of 49.7 million per sample and input files were obtained
Antibodies	anti-H2K27ac antibody (Active motif; Cat#39133)
Peak calling parameters	For peak calling of ChIP-enriched regions, the MACS2 peak caller software (version 2.1.1) of each ChIP to corresponding input DNA sample was used to determine binding regions based on an FDR adjusted pvalue (q-value) <0.25.
Data quality	Deduplicated sequences, minimum length of 25 bp, Phred Score >20
Software	Trim Galore, Bowtie2, MACS2, Picard/MarkDuplicates, Samtools, Deeptools
