# Supplementary figures and images for: Cancer-associated fibroblast-derived acetate promotes pancreatic cancer development by altering polyamine metabolism via the ACSS2–SP1–SAT1 axis
Source: Nat Cell Biol. 2024 Mar 1;26(4):613–27. doi: 10.1038/s41556-024-01372-4 (PMC11021164; doi:10.1038/s41556-024-01372-4)

Unprocessed western blot for Fig. 1e

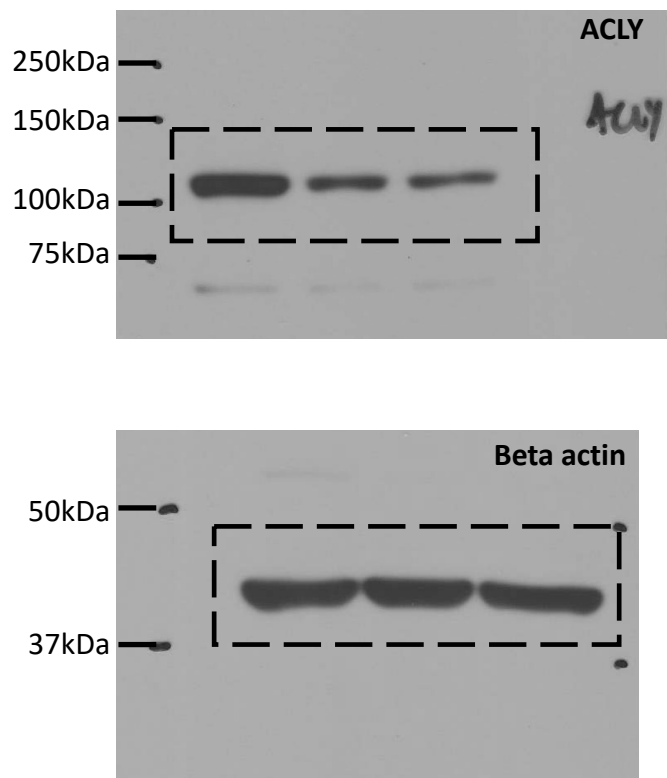

Supplement: Supplementary file 5 — Unprocessed western blots [file 41556_2024_1372_MOESM5_ESM.pdf]

Unprocessed western blot for Fig. 3c

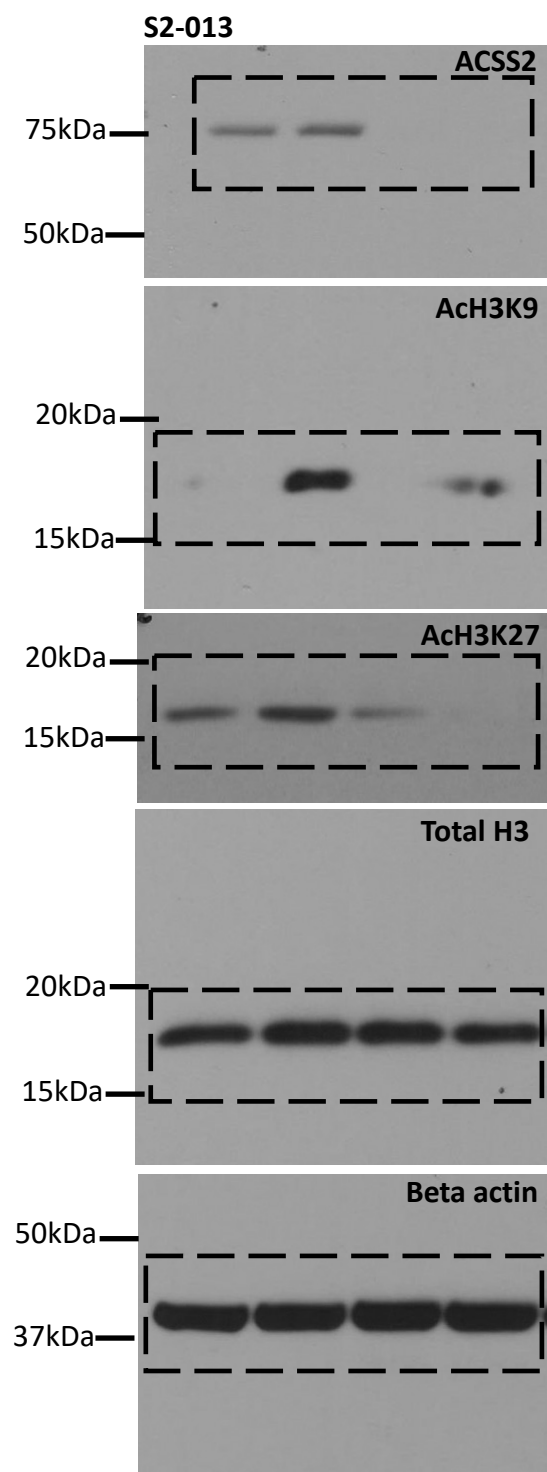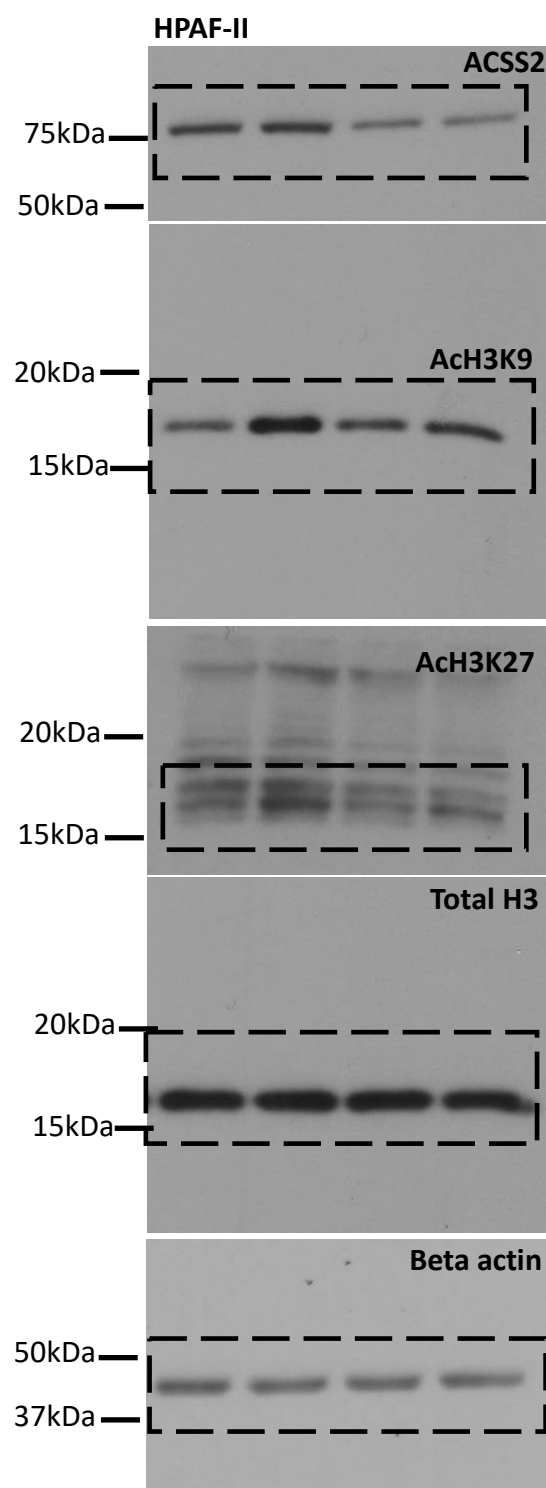

Supplement: Supplementary file 9 — Unprocessed western blots [file 41556_2024_1372_MOESM9_ESM.pdf]

Unprocessed western blot for Fig. 4I

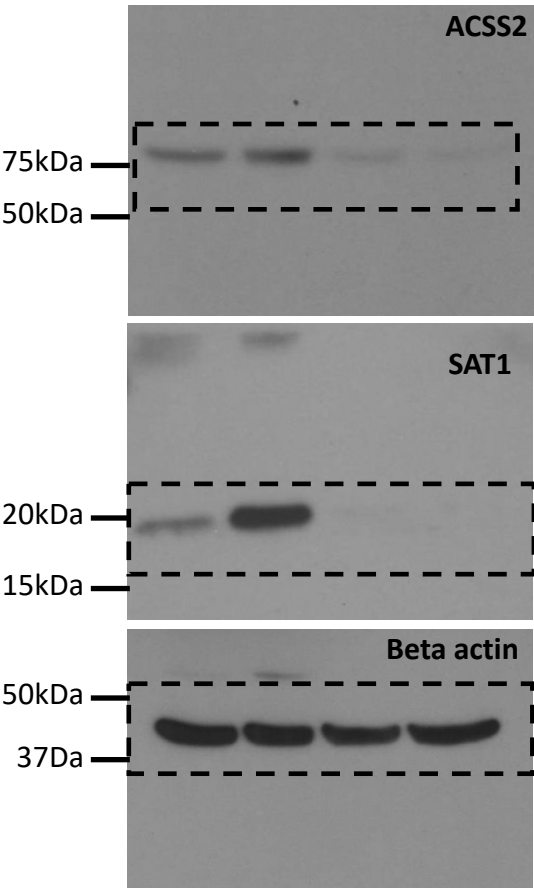

Supplement: Supplementary file 11 — Unprocessed western blots [file 41556_2024_1372_MOESM11_ESM.pdf]

Unprocessed western blot for Fig. 5a

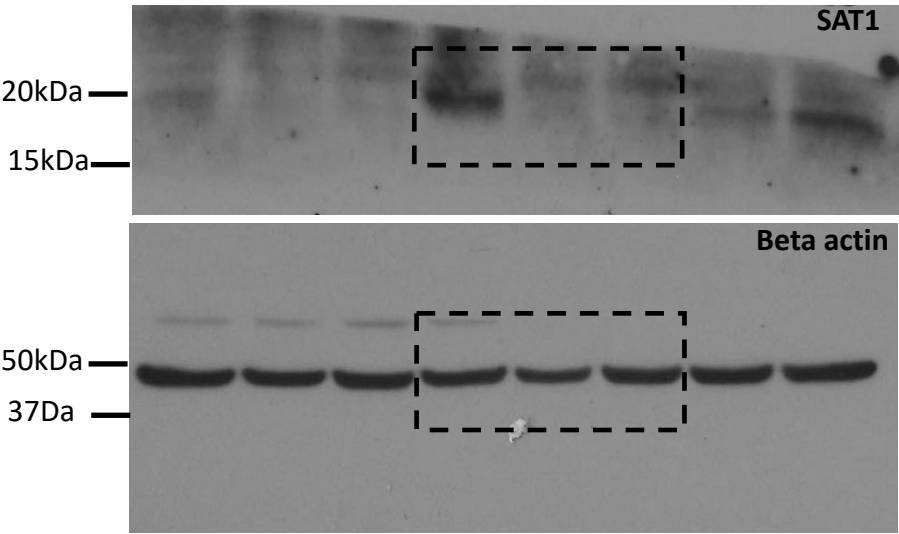

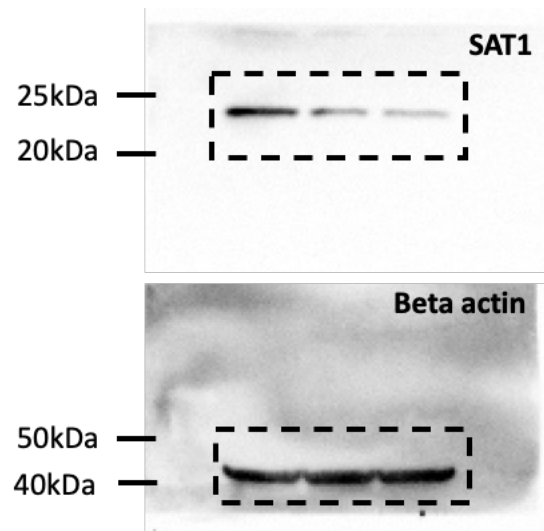

Supplement: Supplementary file 13 — Unprocessed western blots [file 41556_2024_1372_MOESM13_ESM.pdf]

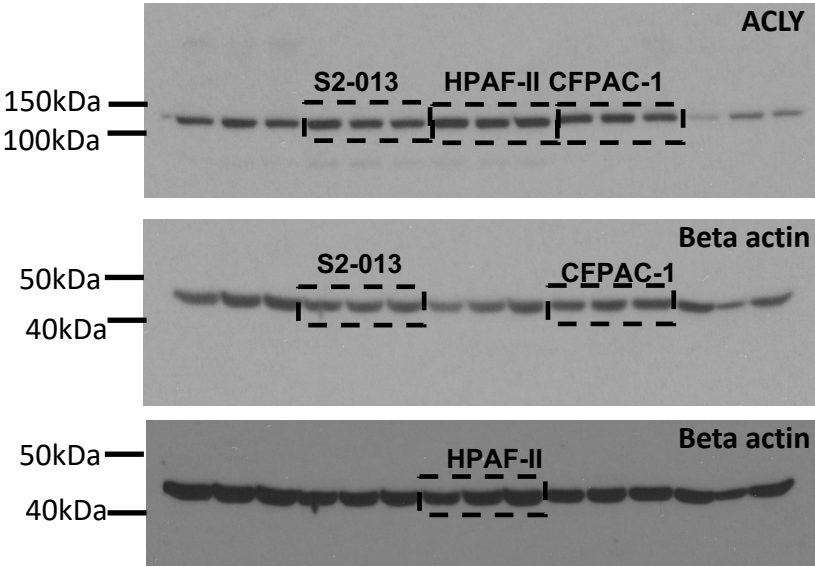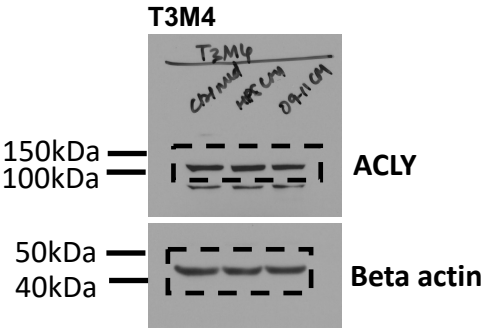

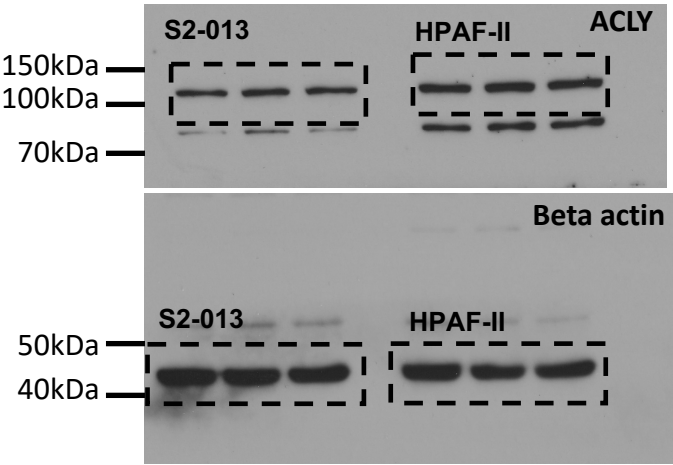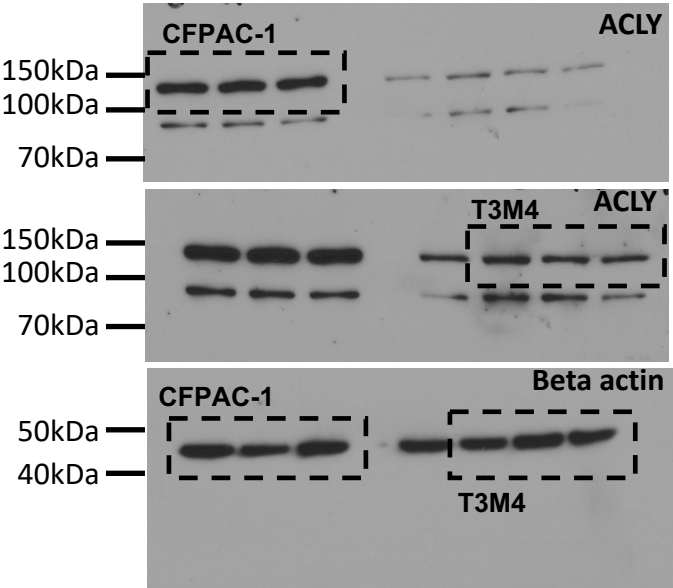

Supplement: Supplementary file 19 — Unprocessed western blots [file 41556_2024_1372_MOESM19_ESM.pdf]

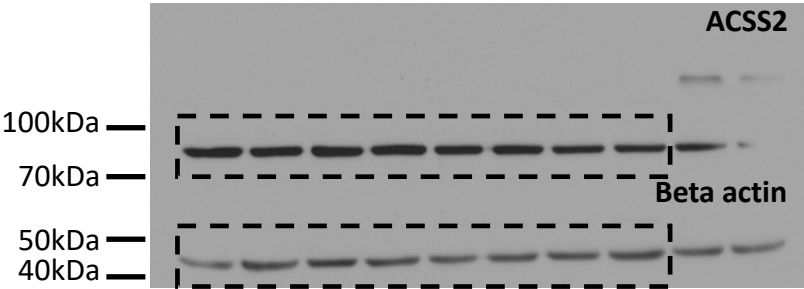

Supplement: Supplementary file 22 — Unprocessed western blots [file 41556_2024_1372_MOESM22_ESM.pdf]

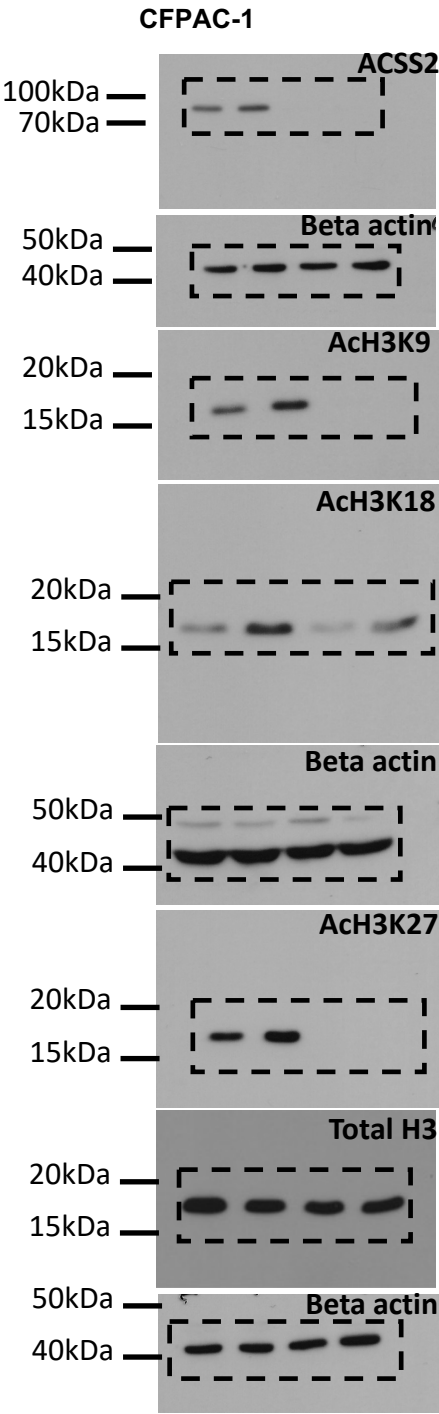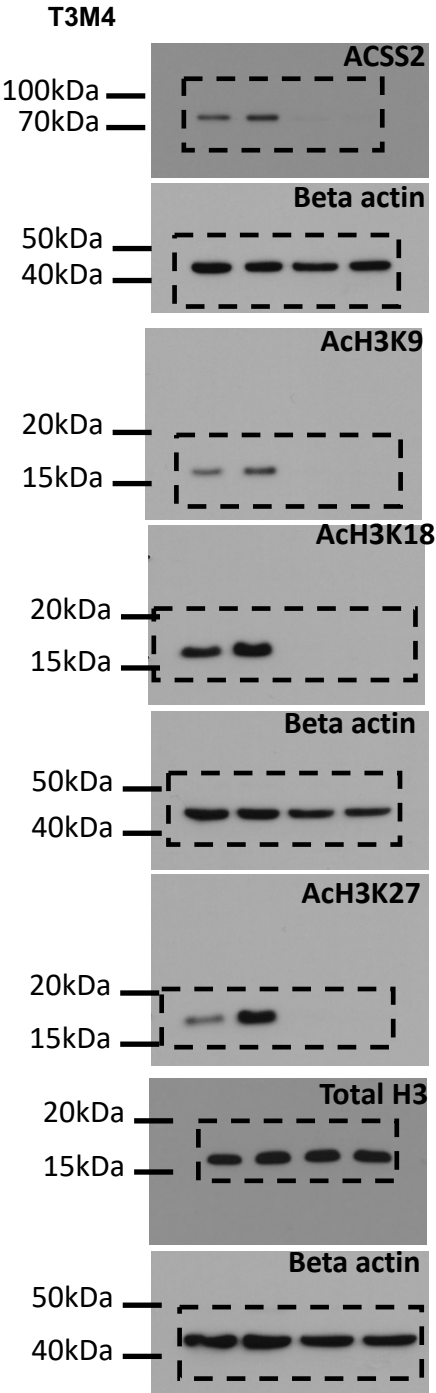

Unprocessed western blot for Extended Data Fig. 5d

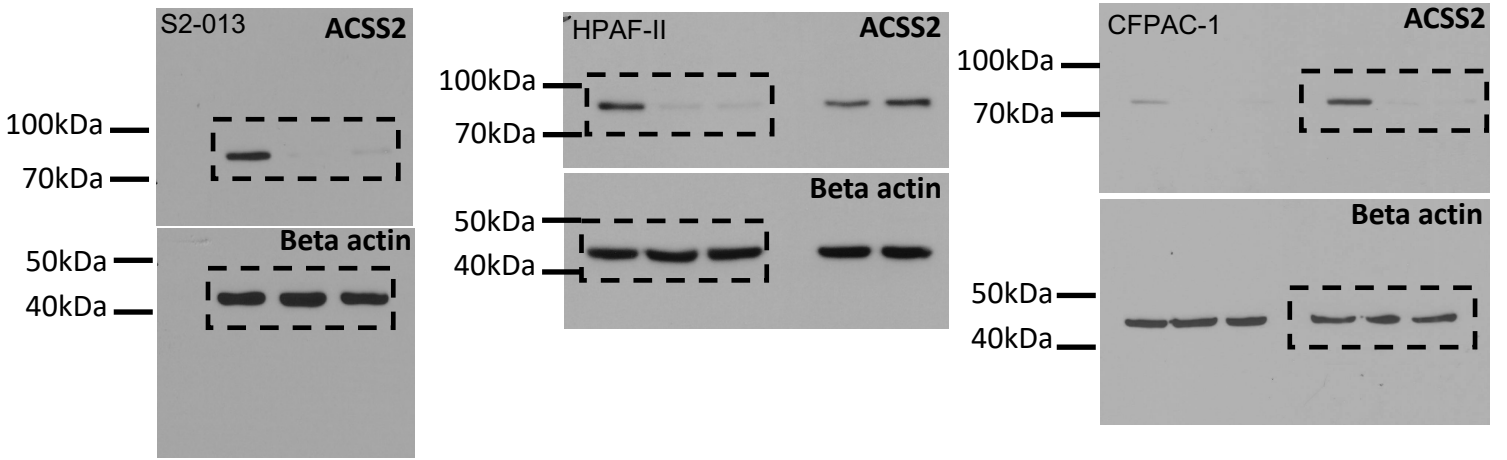

Supplement: Supplementary file 24 — Unprocessed western blots [file 41556_2024_1372_MOESM24_ESM.pdf]

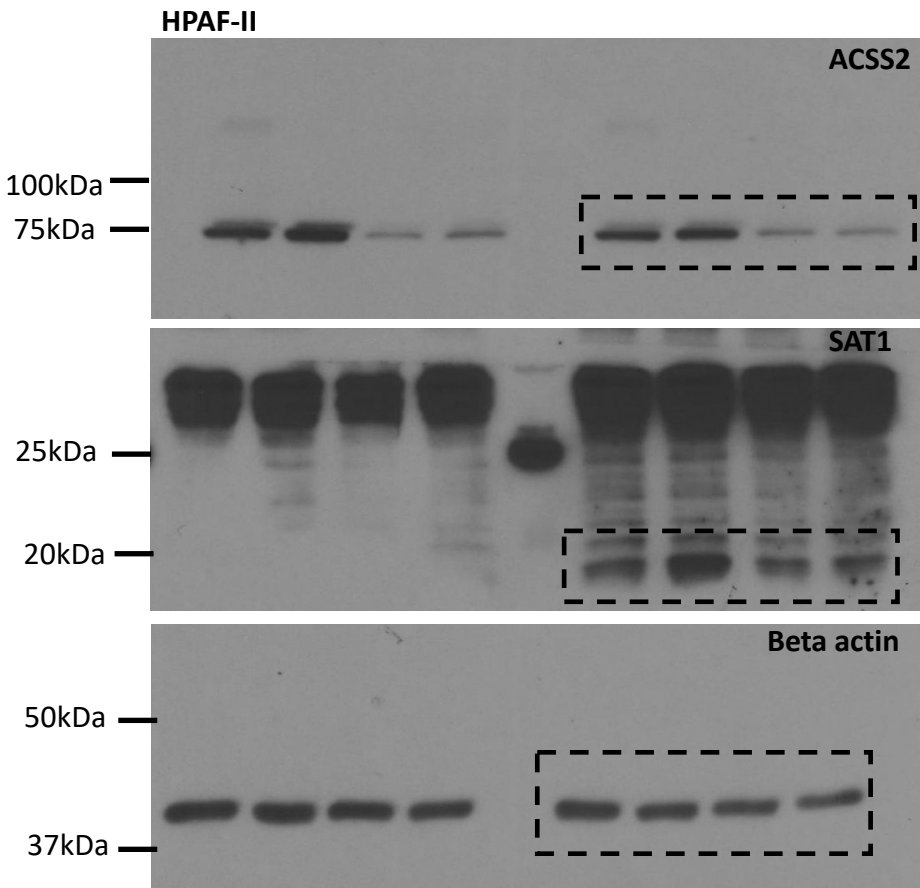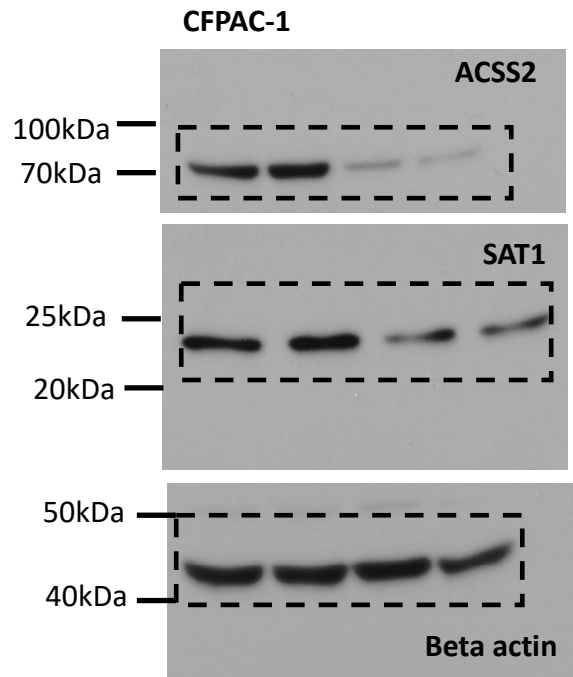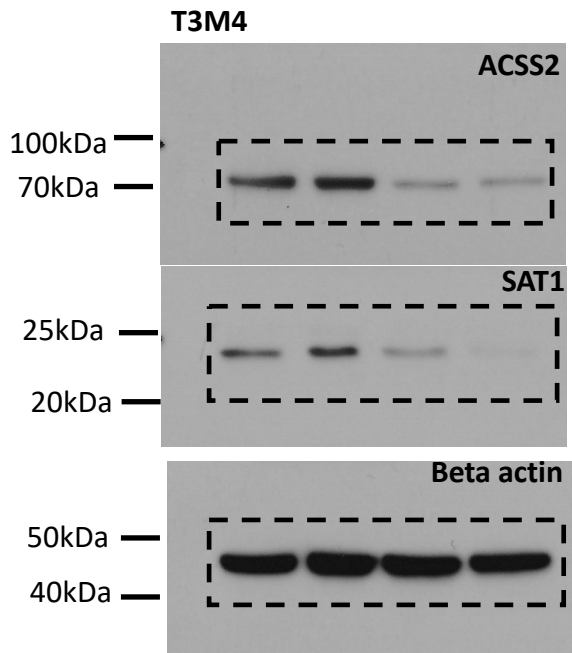

Supplement: Supplementary file 27 — Unprocessed western blots [file 41556_2024_1372_MOESM27_ESM.pdf]

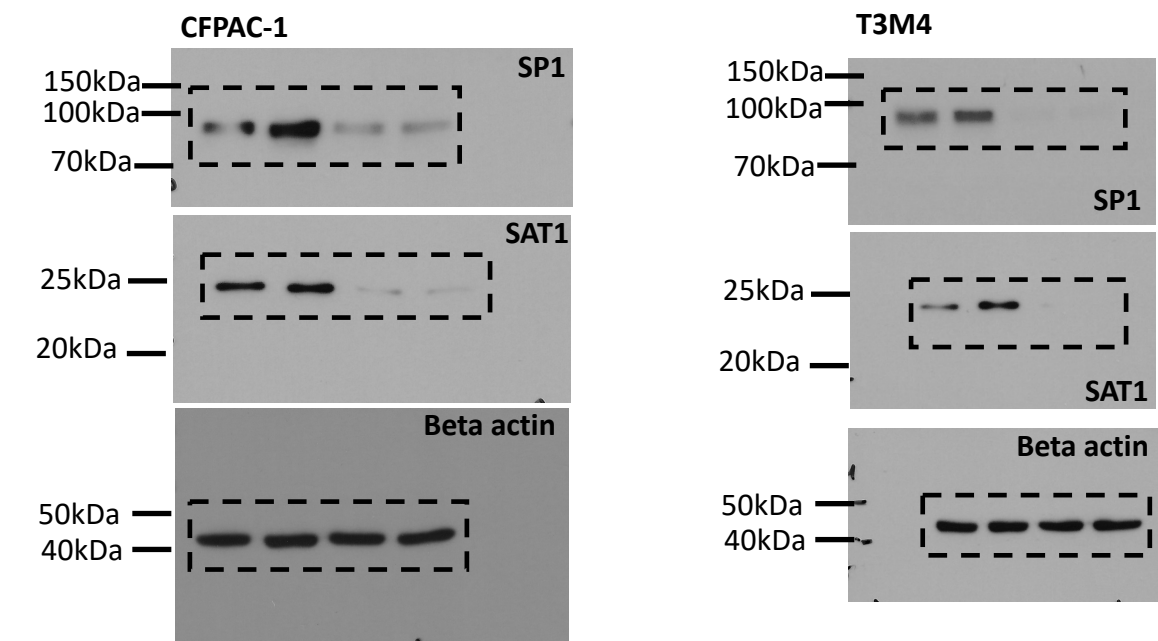

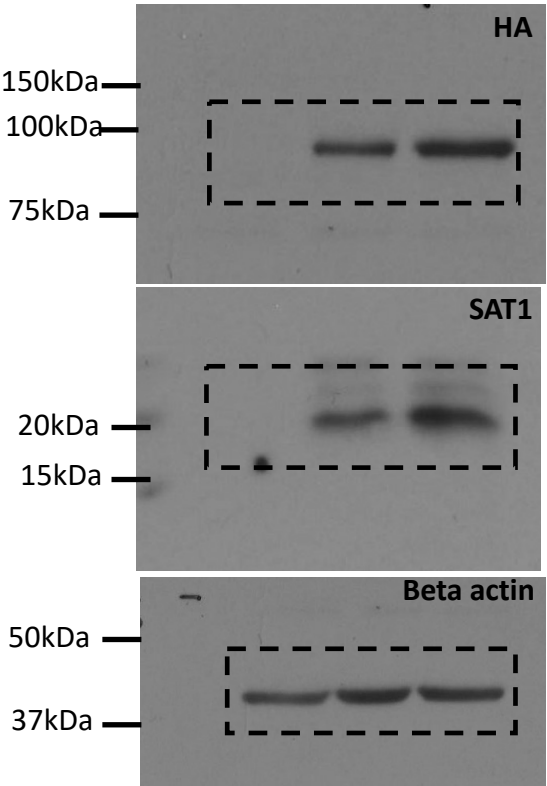

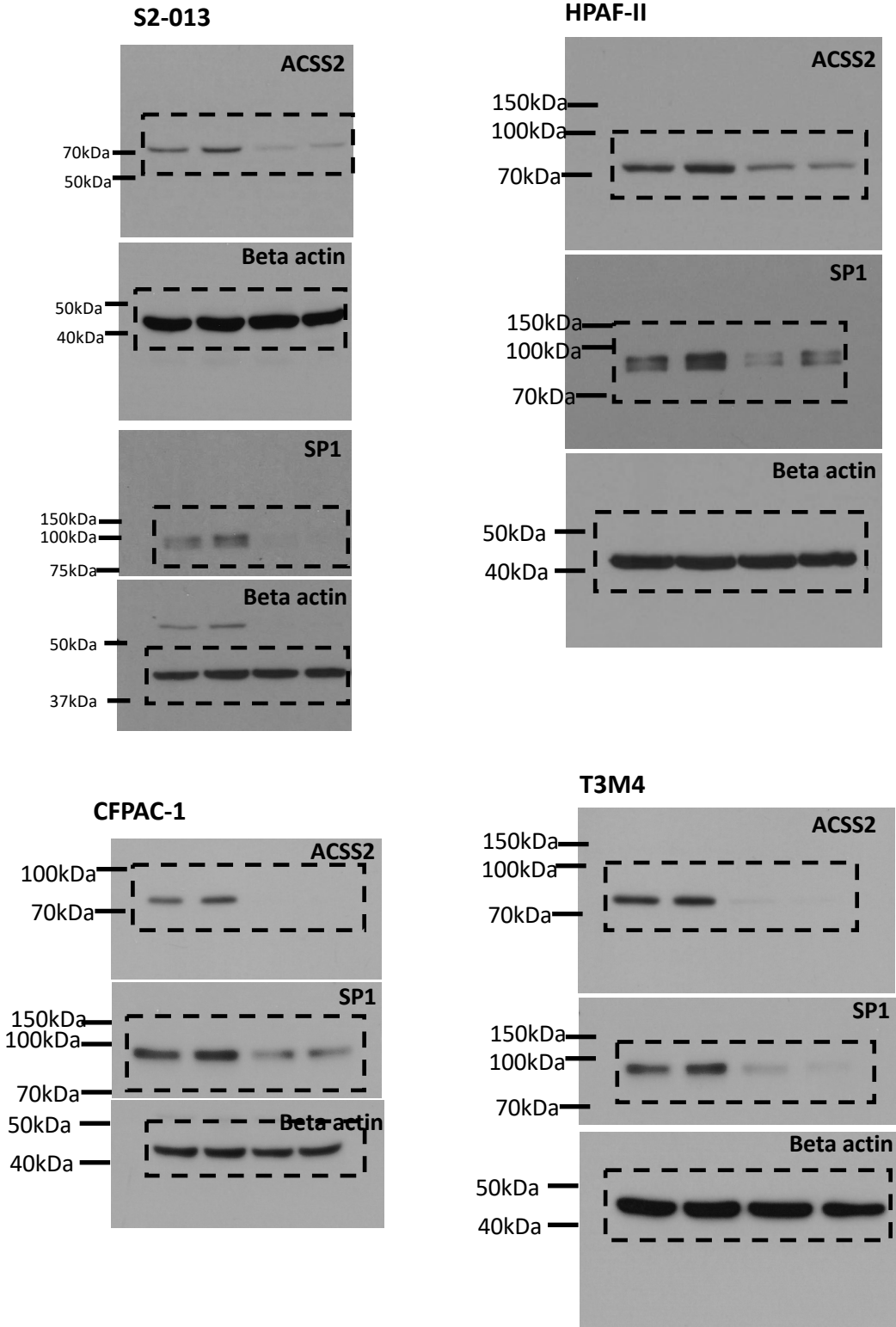

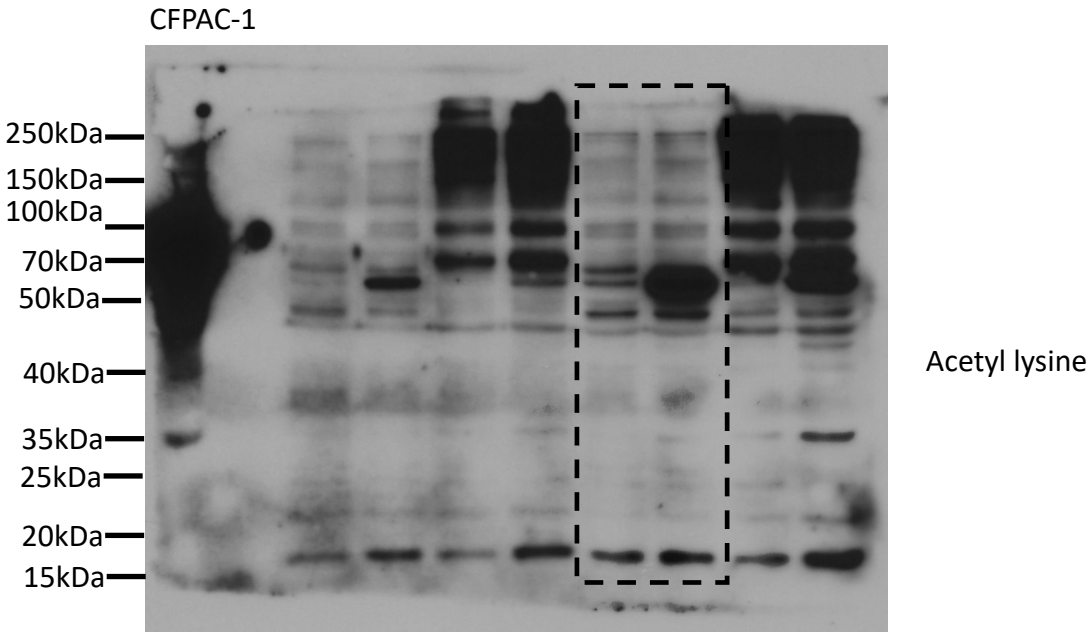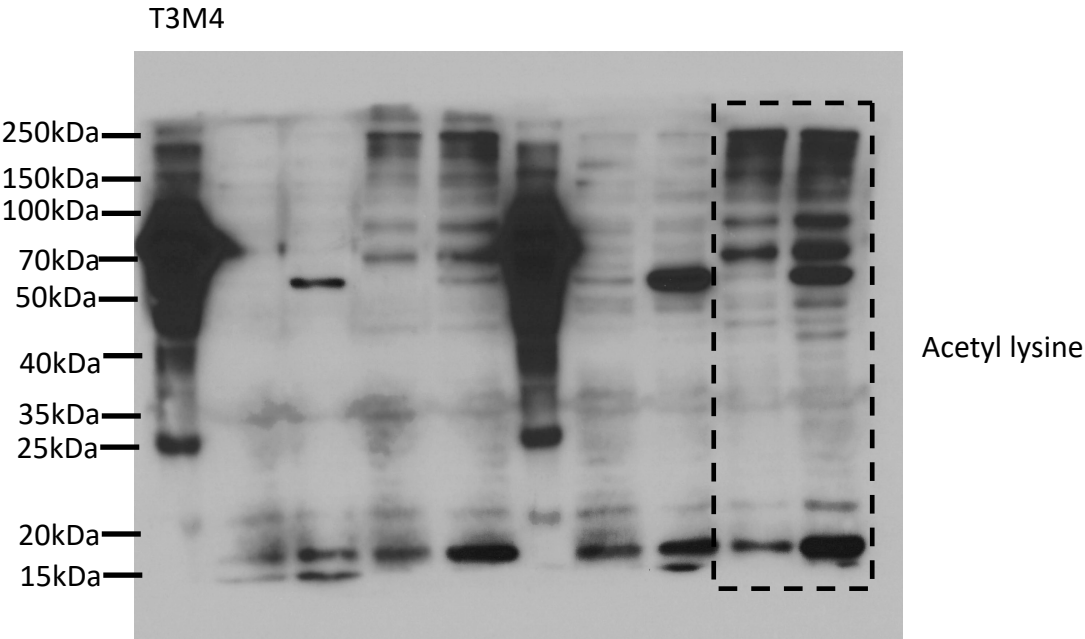

Supplement: Supplementary file 29 — Unprocessed western blots [file 41556_2024_1372_MOESM29_ESM.pdf]
