# Supplementary material for: Cancer-associated fibroblast-derived acetate promotes pancreatic cancer development by altering polyamine metabolism via the ACSS2–SP1–SAT1 axis
Source: Nat Cell Biol. 2024 Mar 1;26(4):613–27. doi: 10.1038/s41556-024-01372-4 (PMC11021164; doi:10.1038/s41556-024-01372-4)

Unprocessed western blot for Fig. 2e

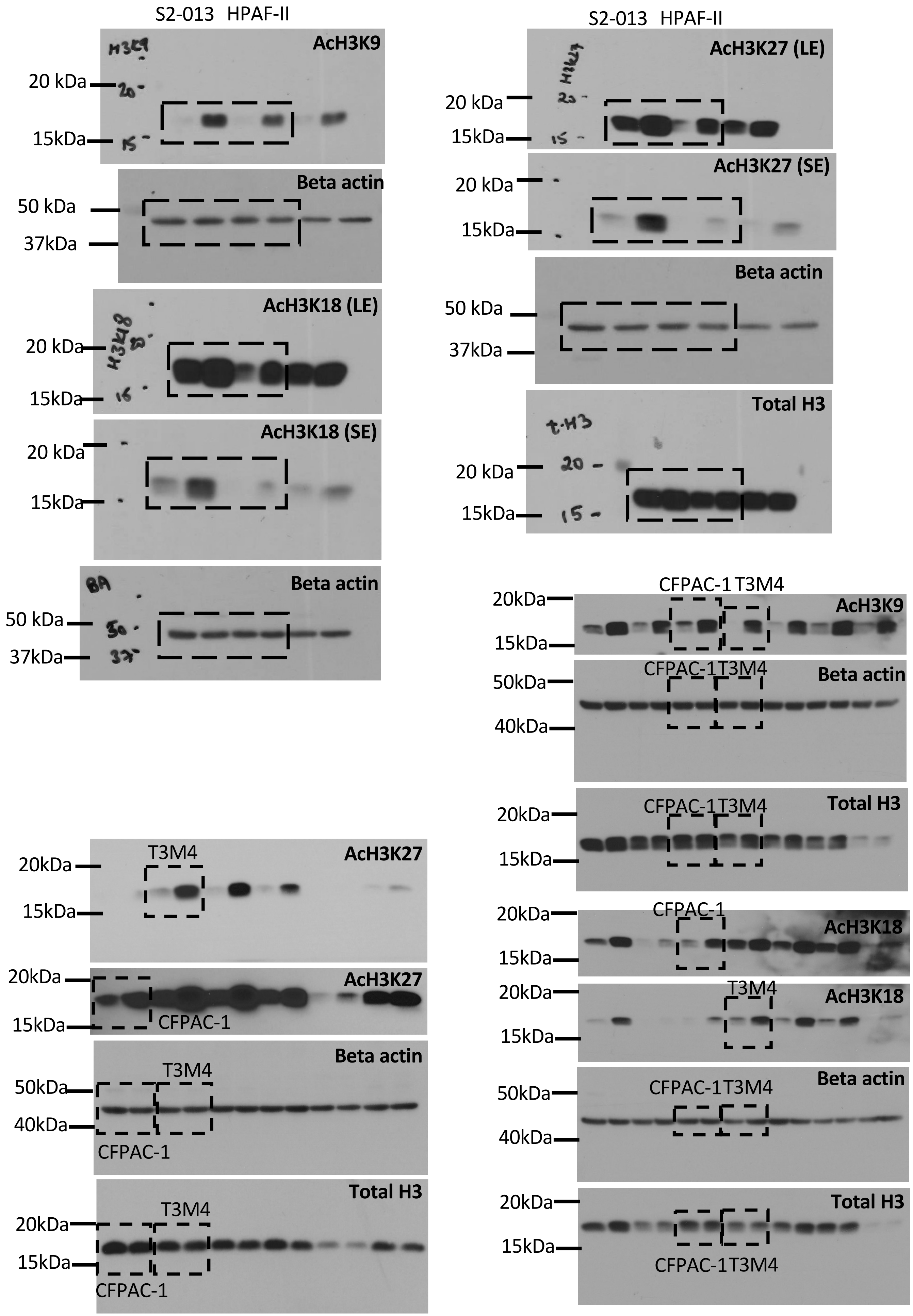

Unprocessed western blot for Fig. 2f

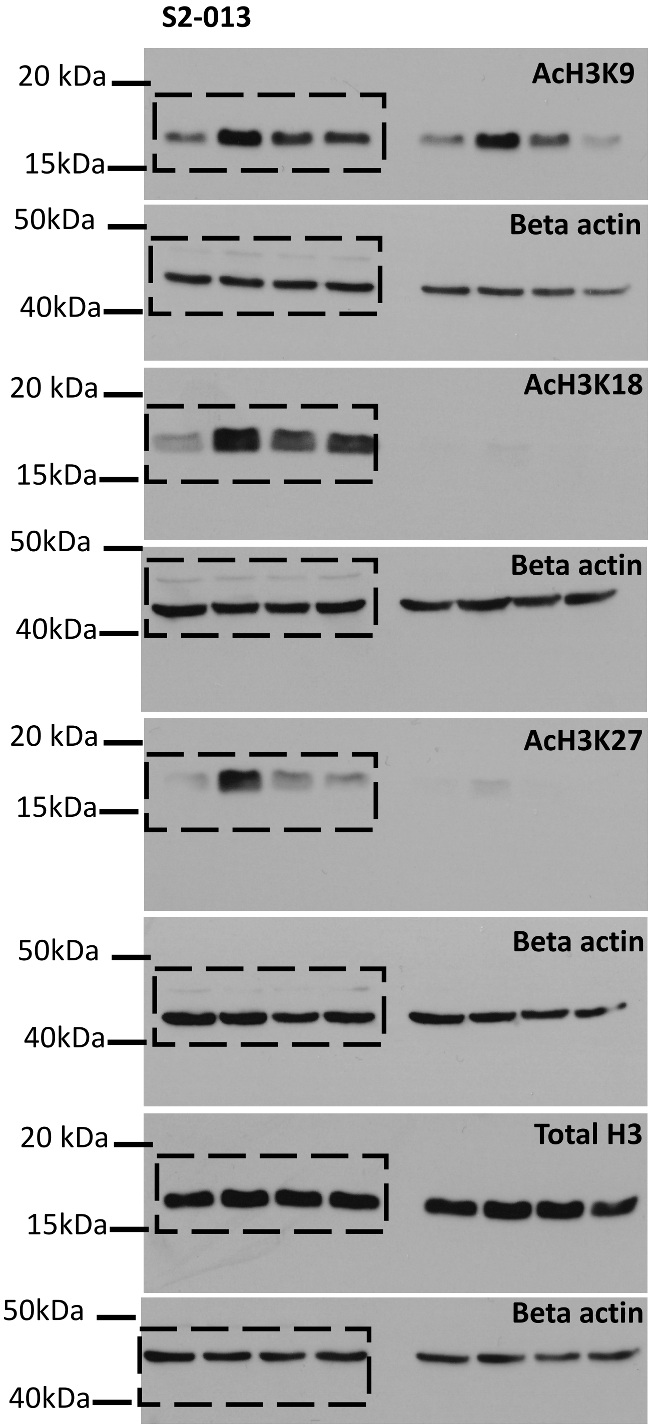

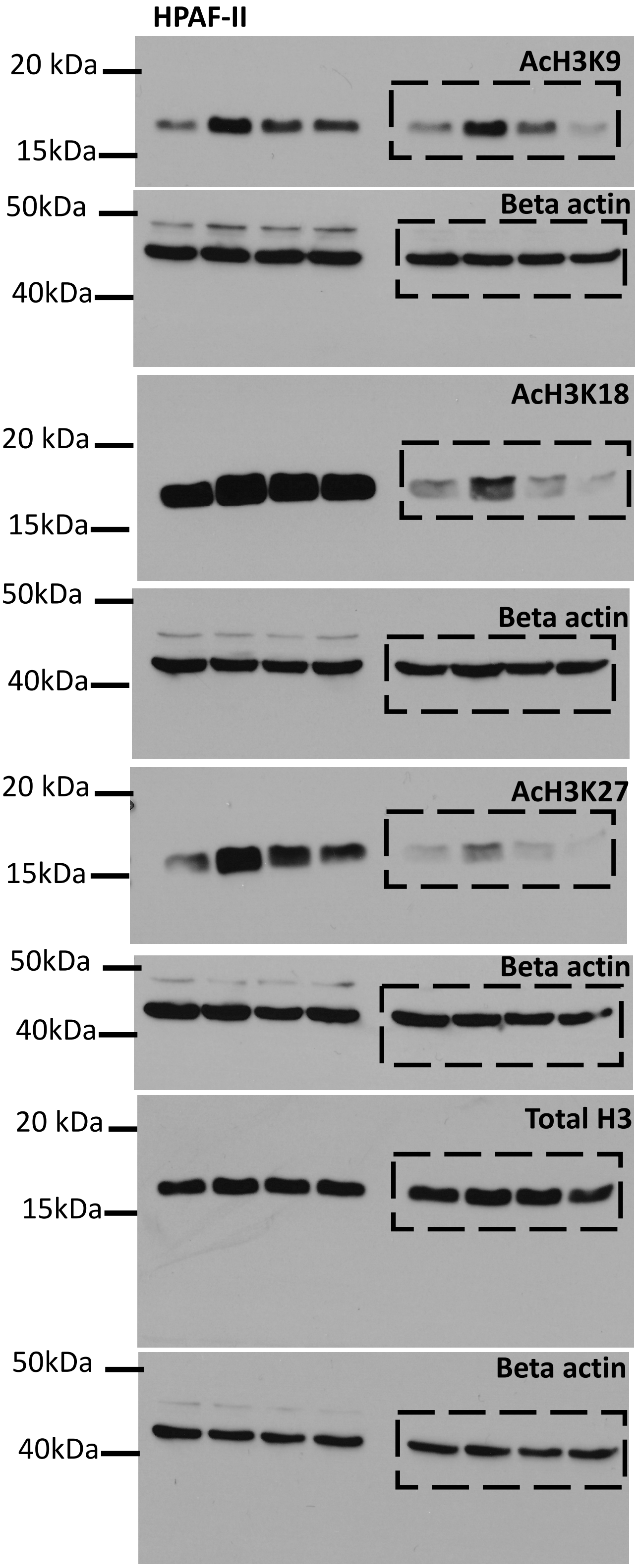

Unprocessed western blot for Fig. 2f (continued)

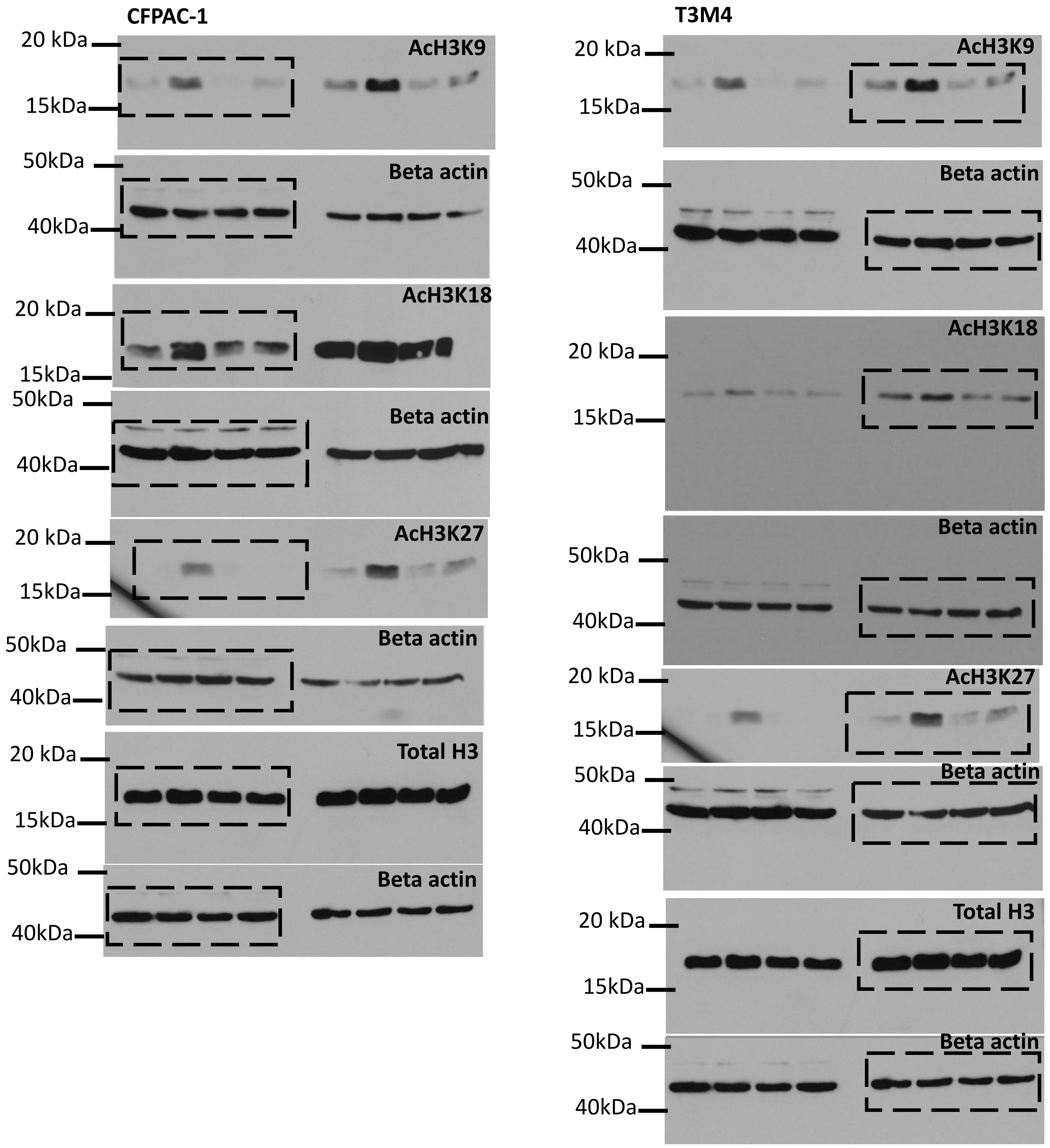

Unprocessed western blot for Fig. 2j

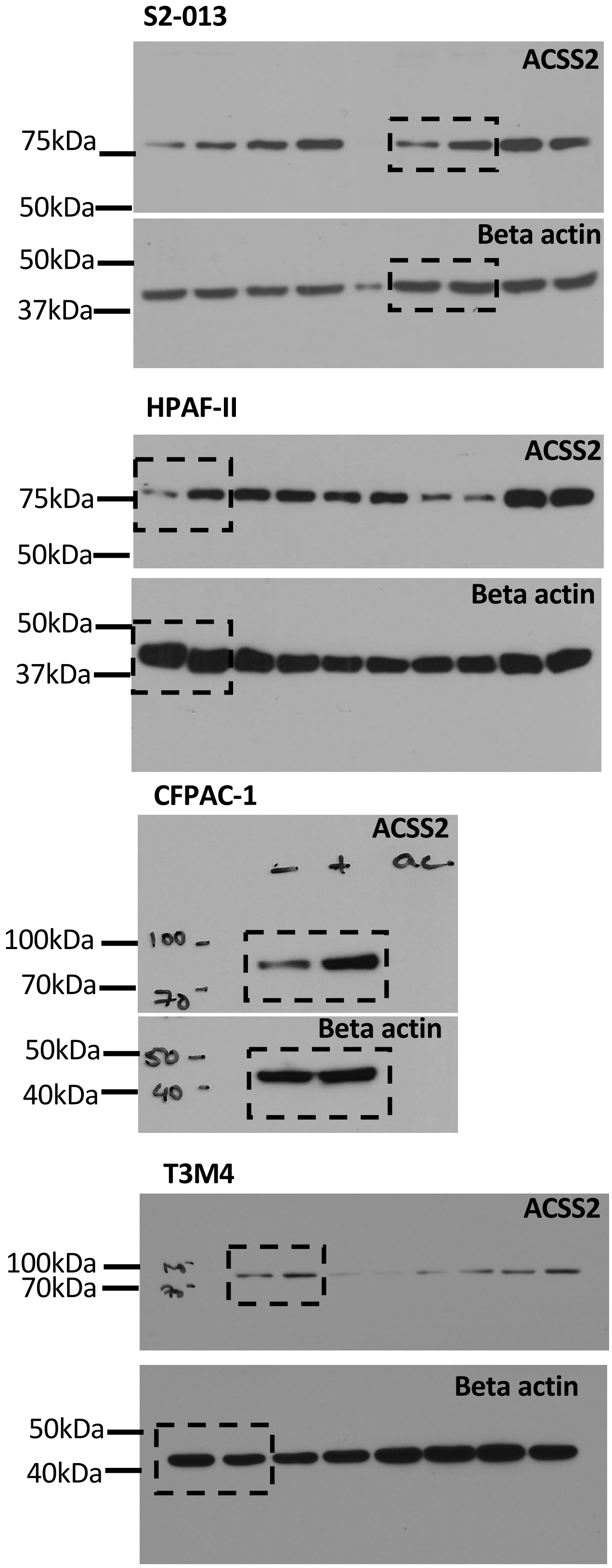

Supplement: Supplementary file 7 — Unprocessed western blots [file 41556_2024_1372_MOESM7_ESM.pdf]
