# Supplementary material for: Author Correction: Cancer-associated fibroblast-derived acetate promotes pancreatic cancer development by altering polyamine metabolism via the ACSS2–SP1–SAT1 axis
Source: Nat Cell Biol. 2026 Aug 4;28(9):1975. doi: 10.1038/s41556-026-02055-y (PMC13561866; doi:10.1038/s41556-026-02055-y)
Supplement: Supplementary file 1 [file 41556_2026_2055_MOESM1_ESM.pdf]

nature cell biology

Supplementary information

Author Correction: Cancer-associated fibroblast-derived acetate promotes pancreatic cancer development by altering polyamine metabolism via the ACSS2–SP1–SAT1 axis

In the format provided by the authors and unedited

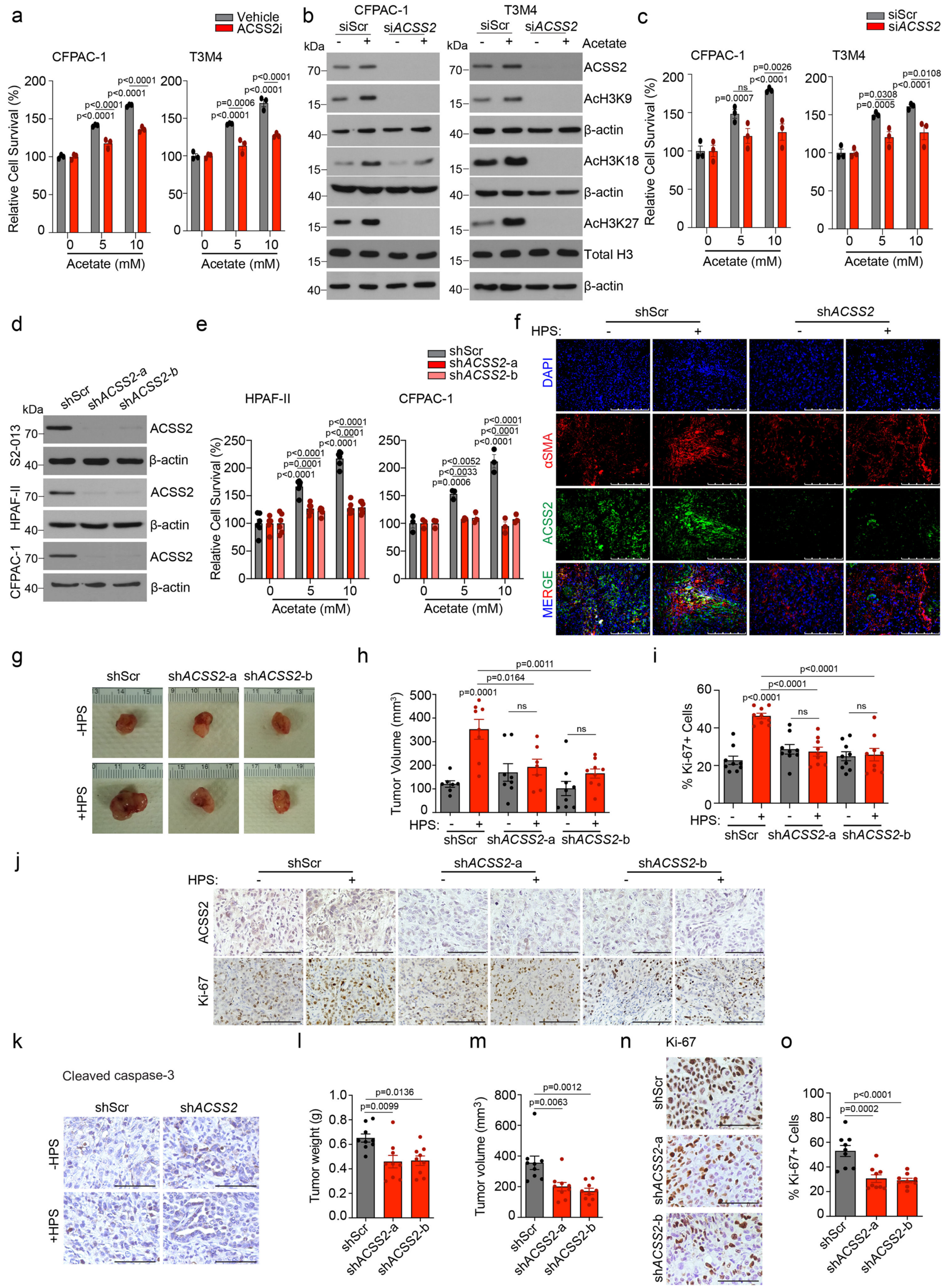

Extended Data Fig. 5
